# Integration of phytochemical profiling and computational approaches to evaluate the neuroprotective potential of *Nardostachys jatamansi* in Alzheimer's disease

**DOI:** 10.1016/j.btre.2025.e00881

**Published:** 2025-02-08

**Authors:** Abdul Jalil Shah, Mohammad Younis Dar, Mohd Adnan, Tanmaykumar Varma, Dhairiya Agarwal, Prabha Garg, Reyaz Hassan Mir, Rampratap Meena, Mubashir Hussain Masoodi

**Affiliations:** a Pharmaceutical Chemistry Division, Department of Pharmaceutical Sciences, University of Kashmir, Hazratbal, Srinagar-190006, Jammu and Kashmir, India; b Drug Standardization Research Unit, Regional Research Institute of Unani medicine (CCRUM), Naseem Bagh campus, University of Kashmir, Srinagar, Jammu and Kashmir, India. 190006; c Department of Biology, College of Science, University of Ha'il, Ha'il, P.O. Box 2440, Saudi Arabia; d National Institute of Pharmaceutical Education and Research, S.A.S. Nagar Mohali, 160062, Punjab India; e Central Council for Research in Unani medicine (CCRUM), 61-65, opp. D-Block, Institutional Area, Janakpuri, New Delhi,110058, India

**Keywords:** *Nardostachys jatamansi*, GCMS, Molecular docking, Network pharmacology, MD simulations

## Abstract

Despite broad spectrum utility of *Nardostachys jatamansi* (D. Don) DC, little is known about the molecular processes that underlie its anti-Alzheimer action. To investigate the molecular targets and therapeutic potential of *N. jatamansi* for Alzheimer's disease (AD), we used Gas Chromatography-Mass Spectrometry (GC-MS), ADMET analysis, network pharmacology, differential gene expression analysis, molecular docking, and molecular dynamics (MD) simulations. The STITCH database was used for network creation and protein-protein interaction analysis, while Cytoscape was used for network visualization and Kyoto Encyclopedia of Genes and Genomes (KEGG) pathway enrichment and Gene Ontology (GO) for term enrichment. Additionally, to investigate the intermolecular interactions between the active chemicals and target proteins, molecular docking experiments were conducted using the Blind docking on the Achilles server. The stability of the PS1 gene complex with Spirojatamol, was further evaluated using MD simulations. With Spirojatamol showing the highest binding energy scores against PS1 (−6.9 kcal/mol), molecular docking confirmed the activity of this metabolite against AD targets PS1 and Spirojatamol formed a stable complex at 100 nanoseconds, according to additional investigation using MD simulations. Significant ligand-protein interactions were verified by binding free energy calculations using the MM/GBSA technique. The PS1-Spirojatamol complex had a binding energy of ΔG: −36.95 ± 5.00 kcal/mol. By focusing on several genes and pathways, involved in AD, this work reveals the molecular underpinnings behind *N. jatamansi* possible use in the treatment of AD.

## Highlights

• *Nardostachys jatamansi* has a broad range of ethnopharmacological applications.
• GC–MS, ADMET, network pharmacology, and gene expression were used for target identification.
• Docking revealed Spirojatamol has high binding affinity with PS1, a potential AD target.
• Molecular dynamics showed a stable PS1-Spirojatamol complex over a 100 ns simulation.
• This study supports the neuroprotective role of N. jatamansi via multiple pathways in AD.

**Abbreviations**
ACE1/Ang II/AT1R: Angiotensin-Converting Enzyme 1/Angiotensin II/Angiotensin II Receptor Type 1
ADMET: Absorption, Distribution, Metabolism, Excretion, and Toxicity
APH1B: Anterior Pharynx Defective 1 Homolog B
APP: Amyloid Precursor Protein
APOE: Apolipoprotein E
BDNF: Brain-Derived Neurotrophic Factor
BSTFA: N,O-Bis(trimethylsilyl)trifluoroacetamide
cAMP/PKA: Cyclic Adenosine Monophosphate/Protein Kinase A Pathway
CREB: cAMP Response Element-Binding Protein:
CSAR1: Cellular Stress-Apoptosis Regulator 1
ESR1: Estrogen Receptor 1
GALR2: Galanin Receptor 2
GO: Gene Ontology
hERG: Human Ether-à-go-go-Related Gene
IL-6: Interleukin 6
KEGG: Kyoto Encyclopedia of Genes and Genomes
NCSTN: Nicastrin
NGF: Nerve Growth Factor
NOS3: Nitric Oxide Synthase 3
NO/cGMP/PKG: Nitric Oxide/Cyclic Guanosine Monophosphate/Protein Kinase G Pathway
NPY1R: Neuropeptide Y Receptor Y1
NR2B: NMDA Receptor Subunit 2B
OMIM: Online Mendelian Inheritance in Man
PPI: Protein-Protein Interaction
PS1: Presenilin-1
PS2: Presenilin-2
PSEN1: Presenilin 1
SiO₂ NPs: Silicon Dioxide Nanoparticles
SLC6A4: Solute Carrier Family 6 Member 4 (Serotonin Transporter)
STRING: Search Tool for the Retrieval of Interacting Genes/Proteins
TNF: Tumor Necrosis Factor
TrkA: Tropomyosin Receptor Kinase A
TrkB: Tropomyosin Receptor Kinase B
TMCS: Trimethylchlorosilane
Notch1: Notch Receptor 1

## 1 Introduction

**Fig. 1:**
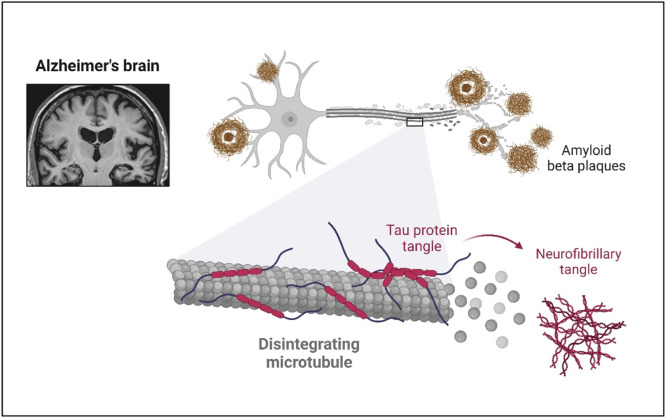
Extracellular deposits of misfolded amyloid-beta proteins disrupt neuronal communication and neurofibrillary tangles causing microtubule disintegration and neuronal dysfunction.

Alzheimer's disease (AD) is the most prevalent form of dementia, characterized by a progressive decline in cognitive functions, including memory, reasoning, and thinking abilities [1]. Initially, individuals may struggle to recall recent events, but as the condition advances, it impairs their capacity to perform everyday tasks, resulting in noticeable behavioral and personality changes [2]. The key pathological markers in the brains of AD patients include beta-amyloid (Aβ) plaques and tau protein tangles [3] (Fig. 1). Additionally, genetic factors such as APOE, APP, PS1, and PS2 are recognized contributors to the disease [4,5]. Currently, around 57 million people worldwide are living with dementia, and this figure is expected to increase many folds by 2050 due to global population aging [6]. The growing number of cases presents serious challenges, including an increased risk of disability, a higher burden of illness, and rising healthcare costs. Existing treatments for AD only manage symptoms, offering limited relief, as no cure has yet been discovered [7]. However, early intervention during the prodromal phase of AD is critical, as it may slow the disease's progression. Therefore, comprehensive research efforts are essential to identify novel protective factors that significantly impact cognitive health.

Notably, approximately 40 % of global dementia cases are linked to modifiable risk factors, suggesting that lifestyle changes could reduce the likelihood of developing the disease [8]. Since 1990, dementia-related mortality rates have shown a persistent rise, increasing from 10.49 deaths per 100,000 people in 1990 to 20.98 deaths per 100,000 in 2019 [9]. Projections indicate that this rate will reach 66.4 deaths per 100,000 by 2040, with most regions expected to experience more deaths. Japan is predicted to have the highest dementia-related mortality rate, estimated at 265 deaths per 100,000 people by 2040 [9]. In response to the growing impact of dementia, the World Health Assembly (WHA) [10] adopted the “Global Action Plan on the Public Health Response to Dementia 2017–2025’’. This initiative emphasizes the need for enhanced efforts in raising awareness, promoting research, and driving innovation to address this escalating public health issue.

Another study estimates that 32 million people globally are living with AD dementia, 69 million with prodromal AD, and 315 million with preclinical AD, totaling 416 million individuals across the AD continuum, which represents 22 % of those aged 50 and above [11]. Most of these individuals are in the preclinical stage, indicating a much larger burden than previously reported. Prevalence varies significantly across regions and age groups, with higher rates observed in high-income Asia-Pacific and North America compared to Sub-Saharan Africa, influenced by factors such as geographic location, education level, and APOE ε4 allele presence. Women are disproportionately affected, constituting 65 % of those with AD dementia and 52 % of the total AD continuum, likely due to their longer life expectancy and higher tau pathology burden [11]. However, significant gaps in data persist, particularly in low- and middle-income countries and during the predementia stages. Large-scale studies and the development of novel methods for early AD detection are critical for advancing diagnostic accuracy.

Recent large-scale studies on medicinal plants have gained significant attention for their potential to treat various human ailments. The chemical diversity found in natural products, including medicinal plants, offers unique opportunities for developing therapeutic agents, particularly anti-Alzheimer compounds [[12], [13], [14]]. These natural remedies are often regarded as safer alternatives to synthetic drugs due to their organic origin and extensive historical use. Numerous preclinical studies have demonstrated that many medicinal plants and their bioactive constituents possess neuroprotective, anti-inflammatory, and antioxidant properties [[14], [15], [16], [17], [18]].

In this study, *Nardostachys jatamansi* (D. Don) DC, a species from the Caprifoliaceae family traditionally used for central nervous system disorders [19], is explored for its anti-Alzheimer's potential. This plant has a wide range of applications, functioning as a stimulant, antispasmodic, tonic, laxative, and antiepileptic agent [20]. It has also demonstrated significant tranquilizing effects and exhibits properties such as hypotensive, hypolipidemic, hepatoprotective, neuroprotective, anti-ischemic, anti-arrhythmic, and anticonvulsant activities [21,22]. The roots and rhizomes of *N. jatamansi* have long been used to treat conditions such as epilepsy, and mental fatigue [23]. Additionally, essential oils extracted from the roots exhibit pharmacological benefits, including antimicrobial, antifungal, hypotensive, anti-arrhythmic, and anticonvulsant activities [24].

We employed an integrated approach combining GC-MS analysis, ADMET profiling, network pharmacology, differential gene expression, molecular docking, and molecular dynamics (MD) simulations to investigate the chemical profile and pharmacological activities of *N. jatamansi* in AD. These methods provide valuable insights into the molecular targets and may ease rational design of inhibitors capable of modulating key pathological processes at the molecular level. Additionally, they offer critical information on the stability and structural dynamics of protein-ligand complexes by simulating physiological conditions.

## 2 Methods

### 2.1 Plant extraction

The rhizomes of *Nardostachys jatamansi* were collected from Uttarakhand, India, and authenticated by Dr. Akhter Malik, a senior taxonomist at the Department of Taxonomy, University of Kashmir. A plant specimen (KU-3237) was deposited in the university's herbarium for reference. A total of 2.0 kg of rhizomes were air-dried and finely ground into powder. The powdered material was extracted using a hydro-ethanol solvent system (70:30), followed by extraction with petroleum ether. The extracts were concentrated under reduced pressure using a rotary evaporator (Buchi R-100)

### 2.2 GC-MS of *Nardostachys jatamansi* petroleum ether fraction

10mL of ethyl acetate and water in the ratio of 1:4 was taken. We added 10µL of sample having concentration of 50mg/mL, and then shaken for few minutes. The top layer was collected and reduced to 0.5mL. To this concentrate, we added 10 µL of pyridine and 50 microlitres of N, O-Bis (trimethylsilyl)trifluoroacetamide and trimethylchlorosilane (9:1) (BSTFA+TMCS). Samples were transferred in GC vial and dried using nitrogen gas. Finally, the samples were reconstituted in methanol for GC-MS analysis.

### 2.3 Prediction of ADMET, BBB permeability and targets of Nerolidol, viridiflorol, spirojatamol and trans-nuciferol against AD

An online tool for predicting the targets of small bioactive compounds is SwissTarget Prediction (www.swisstargetprediction.ch). By importing SMILES and predicting its possible targets, we used this technique to find the relevant targets. The UniProt database was then used to obtain the gene symbols for these targets (http://www.uniprot.org/uniprot/). Additionally, ProTox 3.0 (https://tox.charite.de/protox3/) was used to predict toxicity. We used the term "Alzheimer's disease" to search the GeneCards (https://www.genecards.org/) and OMIM (https://www.omim.org/) databases in order to find genes linked to AD. The UniProt database was used to confirm the names of the identified proteins using official nomenclature and restricting the species source to ‘‘Homo sapiens’’.

### 2.4 Targets intersection, network construction and analysis

Using an online Venn diagram tool (http://bioinformatics.psb.ugent.be/webtools/Venn/), we intersected the possible targets of Nerolidol, viridiflorol, spirojatamol, and trans-nuciferol with AD-related targets to illustrate their interactions. Nerolidol, viridiflorol, spirojatamol, and trans-nuciferol related anti-AD targets were then protein classified using the Panther Classification System (http://www.pantherdb.org/). These possible AD treatment targets were added to the STRING database (https://string-db.org) with the species restriction set to "Homo sapiens" in order to get the Protein-protein interaction (PPI) network interaction. Confidence ratings > 0.4 (medium + high confidence) were found for every interaction within each network. After then, Cytoscape (Version 3.10.2) was used to visualise this network. For each active component, we then identified the top 10 target with the highest scores. In the PPI network, a node with a higher degree value indicated potential important targets for these drugs. The top ten targets were chosen as core targets based on degree.

### 2.5 GO, KEGG enrichment analysis and Differential gene expression

For every test, the significance threshold was set at P < 0.05. To visualize the most relevant enriched KEGG and GO terms, an online tool (http://www.bioinformatics.com.cn/) was used. Initially, gene symbols representing the selected compounds target proteins in AD were entered into the AlzData database (http://www.alzdata.org/) in order to do a correlation study with AD pathologies, such as tau and Aβ. Similarly, for further analyses including GO and KEGG pathway enrichment [25], targets linked to nerolidol, viridiflorol, spirojatamol, and trans-nuciferol that are relevant to AD pathophysiology were employed. Furthermore, utilizing the extensive AlzData database, we performed differential expression analysis to assess the expression levels of risk genes in AD patient's vs healthy controls.

### 2.6 Molecular docking

Computational analyses were performed on a Lenovo THINKSTATION P520 with an Intel Xeon W-2155 processor, Ubuntu 22.04 LTS, 128 GB RAM, an 8 TB hard drive, and an NVIDIA Quadro RTX 6000 GPU.

#### 2.6.1 Protein preparation

Three-dimensional structures of target proteins-Amyloid-beta (Aβ) (PDB ID: 1IYT)1, Tau (PDB ID: 5O3L)2, Amyloid precursor protein (APP) (PDB ID: 1AAP, 2LP1)3,4, Presenilin-1 (PDB ID: 6IYC)5, and Apolipoprotein E (ApoE) (PDB ID: 1GS9, 7FCR) were obtained from the Protein Data Bank. For NMR-derived proteins, the first model was used. Structures were prepared using Schrödinger's Protein Preparation wizard at pH 7.4, including addition of hydrogen atoms, assignment of charges and bond orders, removal of water and interfering ligands, and filling missing loops with Prime. Proteins were minimized using the OPLS4 force field (RMSD ≤ 0.30 Å), then saved in PDBQT format for docking [[26], [27], [28]].

#### 2.6.2 Ligand preparation

Ligands nerolidol, viridiflorol, spirojatamol, and trans-Nuciferol were sourced from PubChem and processed in LigPrep (Schrödinger-2024–1) with protonation states optimized at pH 7.4 using Epik. Ligands were desalted, tautomerized, and stereoisomers generated to yield low-energy 3D structures in MOL2 format, then converted to PDBQT format.

#### 2.6.3 Achilles blind docking

We utilized the Achilles blind docking server due to its widespread recognition in blind docking methodologies [29,30]. The server employs AutoDock Vina as the docking algorithm and executes each docking simulation 100 times, thereby enhancing the reliability and robustness of the results. Blind docking was conducted on the Achilles server, where protein and ligand files were uploaded in PDBQT format. Complexes with the highest docking scores were selected for subsequent molecular dynamics analysis.

### 2.7 Molecular Dynamics simulation

Molecular Dynamics (MD) simulations were conducted to assess the stability and dynamics of receptor-ligand complexes under physiological conditions. The highest-ranked ligand-protein complex, Spirojatamol with PS1, was selected for MD simulations using Schrödinger's Desmond module. The protocol included three steps: system setup, minimization, and simulation. The System Builder module applied the OPLS4 force field, and SPC water molecules were added in a 10 Å × 10 Å × 10 Å orthorhombic box. Na^+^ counterions neutralized the system, with 0.15 M NaCl added. Energy minimization and equilibration were performed under the NPT ensemble at 310 K and 1.01325 bar, followed by a 100 ns MD simulation using the OPLS4 force field. The system was relaxed with default minimization and MD steps before production. Trajectories were analyzed with Desmond's Simulation Interactions Diagram to generate C-alpha RMSD plots [27].

#### 2.7.1 Prime MM-GBSA energy calculations

Binding free energies of receptor-ligand complexes were estimated using Prime MM-GBSA (Molecular Mechanics Generalized Born Surface Area) calculations, which combine molecular mechanics energy, polar solvation (SGB), and non-polar solvation (GNP). The binding free energy (ΔG_bind) was calculated as: ΔG_bind = G_Complex - (G_Ligand + G_Receptor) Where, G = MME + G_SGB + G_NP. Calculations were conducted on MD simulation trajectories using Schrödinger Prime scripts and the VSGB 2.0 solvation model. We conducted MM/GBSA analysis to evaluate the binding free energy profile of the protein-ligand complex. The calculations were performed over the entire 100 ns trajectory, sampling every 10th frame, starting from the 0th frame (0 ns) to the 1000th frame (100 ns). This approach ensured a comprehensive assessment of the binding energy fluctuations throughout the simulation.

## 3 Results

### 3.1 Plant extraction

The coarsely powdered drug (2.0 kg) was extracted with Hydro ethanol (70:30) (%Yield =2.8 w/w) followed by petroleum ether (%Yield = 0.81 w/w).

### 3.2 GC-MS analysis of petroleum ether extract

**Fig. 2:**
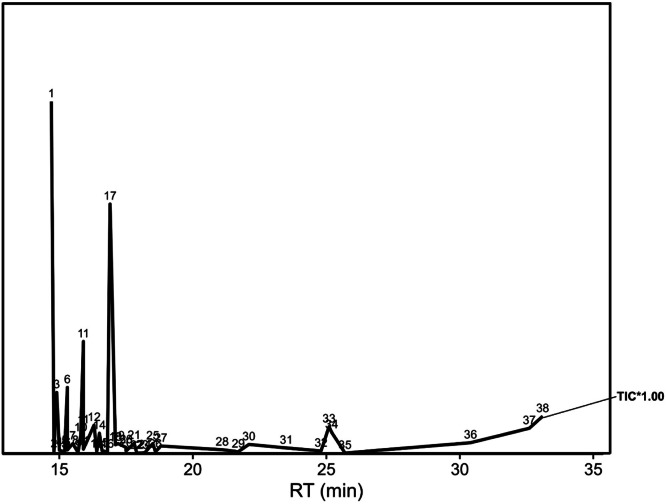
GC-MS chromatogram of *N. Jatamansi* petroleum ether extract.

**Fig. 3:**
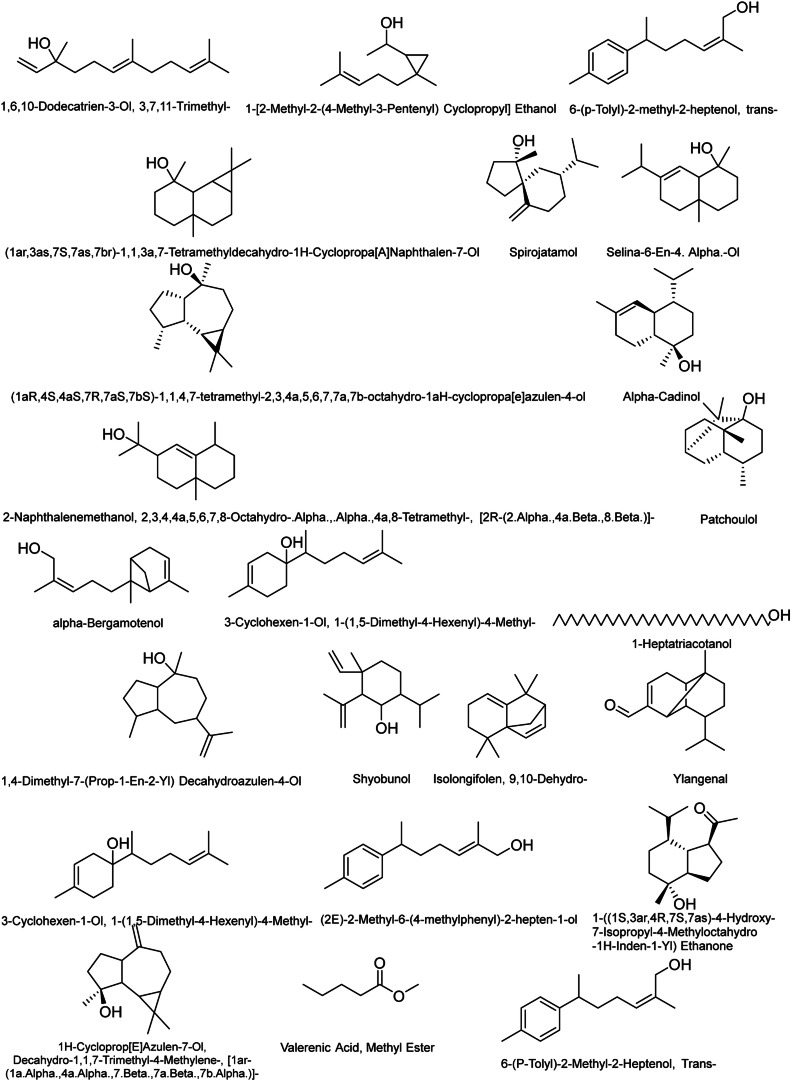

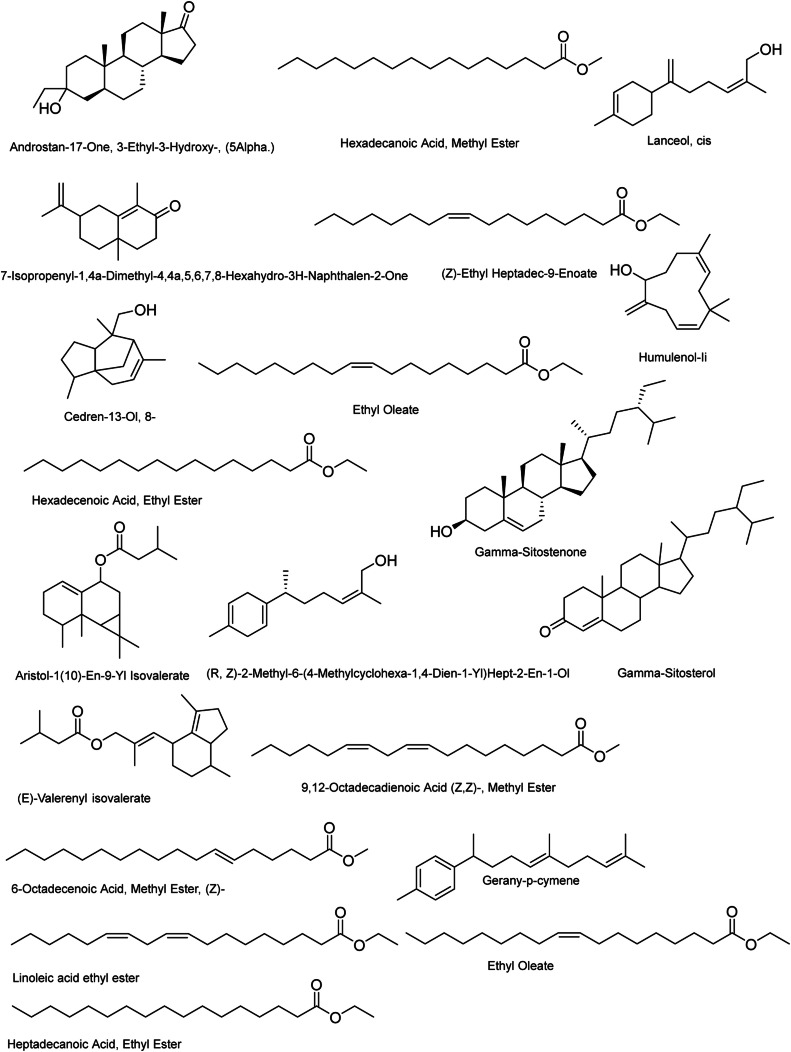
Chemical structure of identified molecules in the petroleum ether extract of *N. jatamansi* rhizomes.

GC-MS chromatogram (Fig. 2) of *N. jatamansi* (rhizome) shows the presence of 43 different bioactive compounds (**Supplementary material S1**). The chemical compounds identified are presented in Table 1. The major constituents present in the petroleum ether extract sample were as; 1,6,10-Dodecatrien-3-Ol, 3,7,11-Trimethyl- (29.08 %), (1ar,3as,7S,7as,7br)-1,1,3a,7-Tetramethyldecahydro-1H Cyclopropa [A]Naphthalen-7-Ol (5.1 %), Spirojatamol (5.51 %), Patchouli alcohol (9.21 %), 1,1,4,7-Tetramethyldecahydro-1H-cyclopropa [e]azulen-4-ol (20.61 %). The details other minor and trace compounds are shown in Table 1, and their respective structures are presented in Fig. 3.

**Table 1:** GCMS analysis with retention time, % peak area, name of compound, molecular weight and their respective SMILES.

Peak No.	RT	Area%	Name	Molecular Weight (g/mol)	SMILES
1	14.650	29.08	1,6,10-Dodecatrien-3-Ol, 3,7,11-Trimethyl- (Nerolidol)	222.37	CC(=CCCC(=CCCC(C)(C=C)O)C)C
2	14.773	0.23	1- [2-Methyl-2-(4-Methyl-3-Pentenyl) Cyclopropyl] Ethanol	182.3	CC(C1CC1(C)CCC=C(C)C)O
3	14.876	5.1	(1ar,3as,7S,7as,7br)-1,1,3a,7-Tetramethyldecahydro-1H-Cyclopropa [A]Naphthalen-7-Ol	222.37	CC1(C2C1C3C(CCCC3(C)O)(CC2)C)C
4	15.010	0.33	(1aR,4S,4aS,7R,7aS,7bS)-1,1,4,7-tetramethyl-2,3,4a,5,6,7,7a,7b-octahydro-1aH-cyclopropa [e]azulen-4-ol (Viridiflorol)	222.37	C [C@@H]1CC [C@H]2 [C@@H]1 [C@H]3 [C@H](C3(C)C)CC [C@]2(C)O
5	15.178	0.19	6-(p-Tolyl)-2-methyl-2-heptenol, trans-	218.33	CC1=CC=C(C=C1)C(C)CC/C=C(/C)\CO
6	15.227	0.50	2-Naphthalenemethanol, 2,3,4,4a,5,6,7,8-Octahydro-.Alpha.,.Alpha.,4a,8-Tetramethyl-, [2R-(2.Alpha.,4a.Beta.,8.Beta.)]-	222.37	CC1CCCC2(C1=CC(CC2)C(C)(C)O)C
7	15.308	5.51	Spirojatamol	222.37	CC(C) [C@@H]1CCC(=C) [C@@]2(C1)CCC [C@]2(C)O
8	15.347	0.35	Selina-6-En-4. Alpha.-Ol	222.36	CC(C)C1=CC2C(CCCC2(C)O)(CC1)C
9	15.503	0.87	Alpha-Cadinol	222.37	CC1=C [C@H]2 [C@@H](CC [C@@]( [C@@H]2CC1)(C)O)C(C)C
10	15.580	0.51	Bergamotol, Z-.Alpha.-Trans-	220.35	CC1=CCC2CC1C2(C)CC/C=C(/C)\CO
11	15.725	0.16	1,4-Dimethyl-7-(Prop-1-En-2-Yl) Decahydroazulen-4-Ol	222.37	CC1CCC2C1CC(CCC2(C)O)C(=C)C
12	15.807	1.56	3-Cyclohexen-1-Ol, 1-(1,5-Dimethyl-4-Hexenyl)-4-Methyl-	222.37	CC1=CCC(CC1)(C(C)CCC=C(C)C)O
13	15.865	9.30	Patchouli alcohol	222.37	C [C@H]1CC [C@@]2( [C@@]3( [C@H]1C [C@H](C2(C)C)CC3)C)O
14	15.899	2.20	Shyobunol	222.37	CC(C)C1CCC(C(C1O)C(=C)C)(C)C=C
15	15.975	0.42	1-Heptatriacotanol	537	CCCCCCCCCCCCCCCCCCCCCCCCCCCCCCCCCCCCCO
16	16.265	2.40	Isolongifolen, 9,10-Dehydro-	202.33	CC1(C)CCC=C2C(C)(C)C3CC12C=C3
17	16.391	0.21	1-((1S,3ar,4R,7S,7as)-4-Hydroxy-7-Isopropyl-4-Methyloctahydro-1H-Inden-1-Yl) Ethanone	238.37	CC(C) [C@@H]1CC [C@@]( [C@H]2 [C@H]1 [C@H](CC2)C(=O)C)(C)O
18	16.469	1.73	(2E)-2-Methyl-6-(4-methylphenyl)-2-hepten-1-ol	218.33	CC1=CC=C(C=C1)C(C)CC/C=C(\C)/CO
19	16.629	0.29	Ylangenal	218.33	CC(C)C1CCC2(C3C1C2C(=CC3)C=O)C
20	16.792	0.19	1H-Cycloprop [E]Azulen-7-Ol, Decahydro-1,1,7-Trimethyl-4-Methylene-, [1ar-(1a.Alpha.,4a.Alpha.,7.Beta.,7a.Beta.,7b.Alpha.)]-	220.35	C [C@@]1(CCC2C1C3C(C3(C)C)CCC2=C)O
21	16.986	20.63	1,1,4,7-Tetramethyldecahydro-1H-cyclopropa [e]azulen-4-ol	222.37	CC1CCC2C1C3C(C3(C)C)CCC2(C)O
22	17.144	0.78	Valerenic Acid, Methyl Ester	116.15	CCCCC(=O)OC
23	17.210	0.88	6-(P-Tolyl)-2-Methyl-2-Heptenol, Trans-	218.33	CC1=CC=C(C=C1)C(C)CC/C=C(/C)\CO
24	17.515	0.56	Androstan-17-One, 3-Ethyl-3-Hydroxy-, (5Alpha.)	318.5	CCC1(CC [C@]2( [C@H](C1)CC [C@@H]3 [C@@H]2CC [C@]4( [C@H]3CCC4=O)C)C)O
25	17.549	0.27	Hexadecanoic Acid, Methyl Ester	270.5	CCCCCCCCCCCCCCCC(=O)OC
26	17.776	0.91	7-Isopropenyl-1,4a-Dimethyl-4,4a,5,6,7,8-Hexahydro-3H-Naphthalen-2-One	218.33	CC1=C2CC(CCC2(CCC1=O)C)C(=C)C
27	17.873	0.10	(Z)-Ethyl Heptadec-9-Enoate	296.5	CCCCCCC/C=C\CCCCCCCC(=O)OCC
28	18.058	0.11	Cedren-13-Ol, 8-	220.35	CC1CCC2C13CC=C(C(C3)C2(C)CO)C
29	18.186	0.14	Ethyl Oleate	310.5	CCCCCCCC/C=C\CCCCCCCC(=O)OCC
30	18.515	0.88	Hexadecenoic Acid, Ethyl Ester	282.5	O=C(OCC)CCCCCCCCCCCCCCC
31	18.582	0.20	Lanceol, cis	220.35	CC1=CCC(CC1)C(=C)CC/C=C(/C)\CO
32	18.846	0.68	Aristol-1 [10]-En-9-Yl Isovalerate	304.5	CC1CCC=C2C1(C3C(C3(C)C)CC2OC(=O)CC(C)C)C
33	21.099	0.37	(R, Z)-2-Methyl-6-(4-Methylcyclohexa-1,4-Dien-1-Yl)Hept-2-En-1-Ol	220.35	CC1=CCC(=CC1) [C@H](C)CC/C=C(/C)\CO
34	21.696	0.21	(E)-Valerenyl isovalerate	304.5	CC1CCC(C2=C(CCC12)C)/C=C(\C)/COC(=O)CC(C)C
35	22.107	0.81	9,12-Octadecadienoic Acid (Z,Z)-, Methyl Ester	294.5	CCCCC/C=C\C/C=C\CCCCCCCC(=O)OC
36	23.463	0.52	6-Octadecenoic Acid, Methyl Ester, (Z)-	296.5	C(=CCCCCCCCCCCC)CCCCC(OC)=O
37	24.805	0.27	Gerany-p-cymene	270.5	CC1=CC=C(C=C1)C(C)CC/C=C(\C)/CCC=C(C)C
38	25.005	2.27	Linoleic acid ethyl ester	308.5	CCCCC/C=C\C/C=C\CCCCCCCC(=O)OCC
39	25.166	1.80	Ethyl Oleate	310.5	CCCCCCCC/C=C\CCCCCCCC(=O)OCC
40	25.742	0.10	Heptadecanoic Acid, Ethyl Ester	298.5	O=C(OCC)CCCCCCCCCCCCCCCC
41	30.410	0.95	Humulenol-Ii	220.35	C/C/1=C/CC(/C=C\CC(=C)C(CC1)O)(C)C
42	32.639	2.14	Gamma-Sitosterol	414.7	CC [C@@H](CC [C@@H](C) [C@H]1CC [C@@H]2 [C@@]1(CC [C@H]3 [C@H]2CC=C4 [C@@]3(CC [C@@H](C4)O)C)C)C(C)C
43	33.045	3.13	Gamma-Sitostenone	412.7	CCC(CCC(C)C1CCC2C1(CCC3C2CCC4=CC(=O)CCC34C)C)C(C)C

### 3.3 Prediction of pharmacokinetics, toxicology and BBB permeability

SwissADME was used to predict the ADME-related characteristics of phytochemicals from *N. jatamansi*, and ProTox 3.0, a database that curates toxicology associated qualities from a large body of literature, was used to create toxicological profiles. Lipinski's rule of five was used to assess their drug-likeness. It is important to keep in mind, nevertheless, that many effective medications do not follow these rules exactly, and natural items or their derivatives frequently show two or three violations. According to Table 2, the majority of the phytochemicals met the rule of five, exhibiting good physicochemical and drug-like characteristics that suggested their potential as therapeutic agents.

**Table 2:** Pharmacokinetic and drug likeness properties of identified molecules.

S.No	TPSA	GI absorption	BBB permeant	Pgp substrate	CYP1A2 inhibitor	CYP2C19 inhibitor	CYP2C9 inhibitor	CYP2D6 inhibitor	CYP3A4 inhibitor	Lipinski violations	Bioavailability Score	Lead likeness violations
1	20.23	High	Yes	No	Yes	No	Yes	No	No	0	0.55	2
2	20.23	High	Yes	No	No	No	No	No	No	0	0.55	2
3	20.23	High	Yes	No	No	Yes	Yes	No	No	0	0.55	2
4	20.23	High	Yes	No	No	Yes	No	No	No	0	0.55	2
5	20.23	High	Yes	No	No	No	No	Yes	No	0	0.55	2
6	20.23	High	Yes	No	No	No	Yes	No	No	0	0.55	2
7	20.23	High	Yes	No	No	No	Yes	No	No	0	0.55	2
8	20.23	High	Yes	No	No	No	Yes	No	No	0	0.55	2
9	20.23	High	Yes	No	No	Yes	No	No	No	0	0.55	1
10	20.23	High	Yes	No	No	Yes	Yes	No	No	0	0.55	2
11	20.23	High	Yes	No	No	Yes	Yes	No	No	0	0.55	2
12	20.23	High	Yes	No	No	No	Yes	No	No	0	0.55	2
13	20.23	High	Yes	No	No	No	Yes	No	No	0	0.55	2
14	20.23	High	Yes	No	No	Yes	Yes	No	No	0	0.55	2
15	20.23	Low	No	Yes	No	No	No	No	No	2	0.17	3
16	0	Low	No	No	No	Yes	Yes	No	No	1	0.55	2
17	37.3	High	Yes	No	No	No	No	No	No	0	0.55	1
18	20.23	High	Yes	No	No	No	No	Yes	No	0	0.55	2
19	17.07	High	Yes	No	No	Yes	Yes	No	No	0	0.55	2
20	20.23	High	Yes	No	No	Yes	No	No	No	0	0.55	1
21	20.23	High	Yes	No	No	Yes	No	No	No	0	0.55	2
22	26.3	High	Yes	No	No	No	No	No	No	0	0.55	1
23	20.23	High	Yes	No	No	No	No	Yes	No	0	0.55	2
24	37.3	High	Yes	No	No	No	No	No	No	1	0.55	1
25	26.3	High	Yes	No	Yes	No	No	No	No	1	0.55	2
26	17.07	High	Yes	No	No	Yes	Yes	No	No	0	0.55	2
27	26.3	High	No	No	Yes	No	No	No	No	1	0.55	2
28	20.23	High	Yes	No	No	No	Yes	No	No	0	0.55	1
29	26.3	Low	No	No	Yes	No	No	No	No	1	0.55	2
30	26.3	High	No	No	Yes	No	No	No	No	1	0.55	2
31	20.23	High	Yes	No	No	No	Yes	No	No	0	0.55	2
32	26.3	High	Yes	No	No	Yes	Yes	Yes	No	1	0.55	1
33	20.23	High	Yes	No	No	No	No	No	No	0	0.55	1
34	26.3	High	Yes	No	No	Yes	Yes	Yes	Yes	1	0.55	1
35	26.3	High	Yes	No	No	Yes	Yes	Yes	Yes	1	0.55	1
36	26.3	High	No	No	Yes	No	No	No	No	1	0.55	2
37	0	Low	No	Yes	No	Yes	Yes	No	No	1	0.55	1
38	26.3	Low	No	Yes	No	Yes	Yes	No	No	1	0.55	1
39	26.3	Low	No	No	Yes	No	No	No	No	1	0.55	2
40	26.3	High	No	No	Yes	No	No	No	No	1	0.55	2
41	20.23	High	Yes	No	No	No	Yes	No	No	0	0.55	1
42	20.23	Low	No	No	No	No	No	No	No	1	0.55	2
43	17.07	Low	No	No	No	No	No	No	No	1	0.55	2

**Fig. 4:**
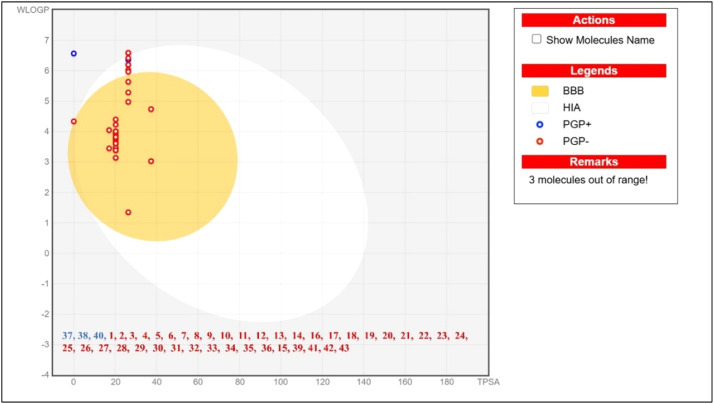
Brain or intestinal estimated permeation plot (BOILED-Egg plot) of identified molecules.

Topological polar surface area (TPSA), gastrointestinal absorption (GIA), blood–brain barrier (BBB) permeability, P-glycoprotein (P-gp), Cytochromes P450 (CYPs) and bioavailability score were among the ADME-related characteristics that were displayed in Table 2. When a drug candidate's TPSA exceeds 140 Å^2^, it may indicate poor absorption. Most of the phytochemicals from *N. jatamansi* had satisfactory TPSA values and GIA. The phytochemicals have a significant bioavailability and effectively pass the cell membrane, as shown by bioavailability score of more than zero. BBB permeability (Fig. 4) and GIA are crucial characteristics of a medication meant for use in central nervous disorders. Many xenobiotics have ADMET features that are influenced by permeability glycoprotein (P-gp), thus it's critical to look into how the transporter protein interacts with the drug molecule. By functioning as a unidirectional efflux pump to expel drug molecules from within to outside of cells, it restricts the absorption and metabolism of substances by cells. Table 2 shows that all of the phytochemicals, with few violations, were not P-glycoprotein substrates, indicating their potential as therapeutic agents. Toxicological parameters were evaluated in silico using the ProTox 3.0. Nerolidol, viridiflorol, spirojatamol, and trans-nuciferol showed no discernible toxicity. They were also able to pass the blood-brain barrier and had a low toxicity level (LD_50_ 2000 mg/kg or above), which made them good drug candidates for further study Table 3.

**Table 3:** Comparative pharmacokinetic and drug likeness properties of spirojatamol with donepezil, rivastigmine and memantine.

Property	Donepezil	Rivastigmine	Memantine	Spirojatamol
Molecular Formula	C_24_H_29_NO_3_	C_14_H_22_N_2_O_2_	C_12_ H_21_ N	C_15_ H_26_ O
Molecular Weight	379.21	250.17	179.17	222.20
LogP	4.46	1.86	3.35	3.41
LogS	-4.31	-2.17	-3.11	-2.85
HBA / HBD	4 / 0	3 / 0	1/2	1/1
MolPSA (Å²)	32.39	25.09	20.01 A^2^	16.04 A^2^
MolVol (Å³)	406.83	277.27	241.40 A^3^	291.45 A^3^
pKa (Basic/Acidic)	8.75 / 18.70	9.23 / 22.98	9.72 / 20.54	<0. / 15.70
BBB Score	5.29	5.38	4.86	4.39
Drug-likeness Score	1.56	1.62	-0.75	-0.49

### 3.4 Screening of targets

**Fig. 5:**
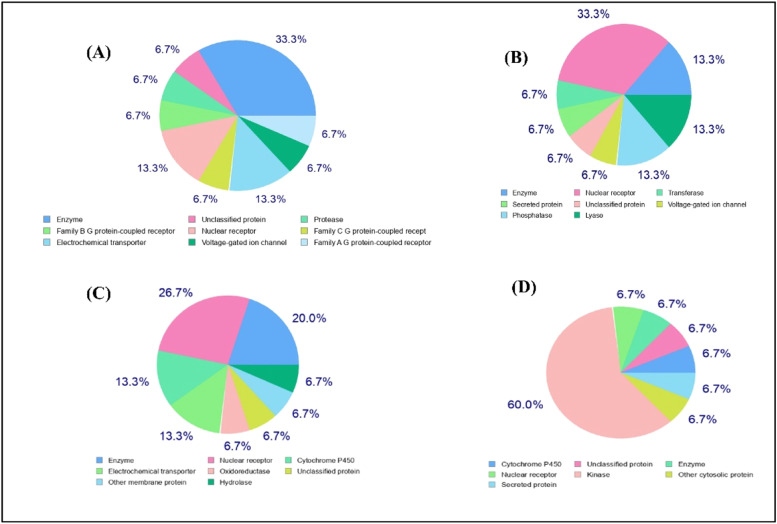
The diverse targets of a) Nerolidol b) viridiflorol c) Spirojatamol d) Trans-nuciferol using the PANTHER classification system.

**Fig. 6:**
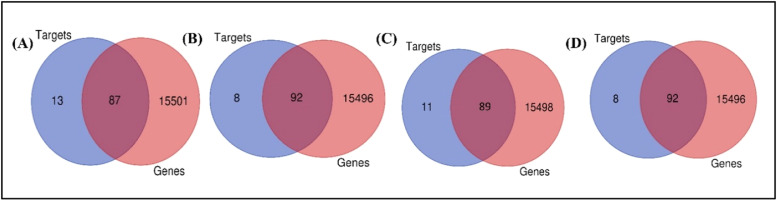
Venn diagram showing intersection of targets of a) Nerolidol b) Viridiflorol c) Spirojatamol d) Trans-nuciferol.

**Fig. 7. a):**
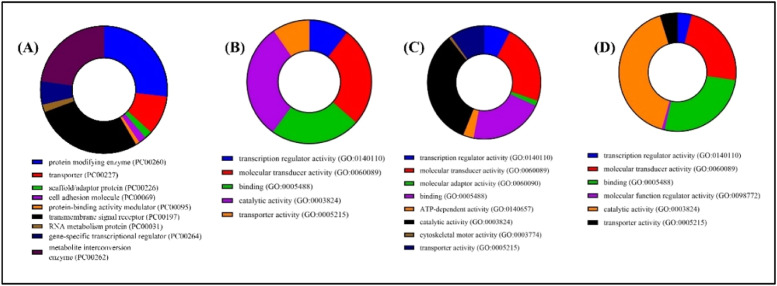
Nerolidol b) viridiflorol c) Spirojatamol d) Trans-nuciferol targets relevant to AD using the PANTHER classification system.

To identify the Nerolidol, Viridiflorol, Spirojatamol and Trans-nuciferol associated targets, 100 targets for each were collected from SwissTargets. The top 15 targets were classified into different classes (Fig. 5) Additionally, OMIM and GeneCard provided disease-related targets. By combining these targets with 15,588 disease-related targets, we were able to identify 87 Nerolidol targets, 92 viridiflorol and trans-nuciferol targets, and 90 spirojatamol targets against AD (Fig. 6). Furthermore, using the Panther Classification System, each of these genes associated with AD was categorized into a unique class (Fig. 7). The top 10 protein classes were transmembrane signal receptor (25.70 %), protein modifying enzyme (24.80 %), metabolite interconversion enzyme (21.10 %), transporter (9.20 %), gene-specific transcriptional regulator (5.50 %), scaffold/adaptor protein (1.80 %), cell adhesion molecule (1.80 %), RNA metabolism protein (1.80 %), protein-binding activity modulator (0.90 %) for Nerolidol. Catalytic activity (30.40 %), molecular transducer activity (26.10 %), binding (23.50 %), transcription regulator activity (10.40 %), transporter activity (9.60 %) for Viridiflorol. Catalytic activity (33.30 %), molecular transducer activity (22.70 %), binding (21.20 %), transporter activity (9.80 %), transcription regulator activity (7.60 %), molecular adaptor activity (1.50 %), ATP-dependent activity (3.00 %), cytoskeletal motor activity (0.80 %) for Spirojatamol. Transcription regulator activity (4.00 %), molecular transducer activity (23.20 %), binding (26.40 %), molecular function regulator activity (0.80 %), catalytic activity (40.80 %), transporter activity (4.80 %) for Trans-nuciferol.

### 3.5 Network construction and screening

**Fig. 8:**
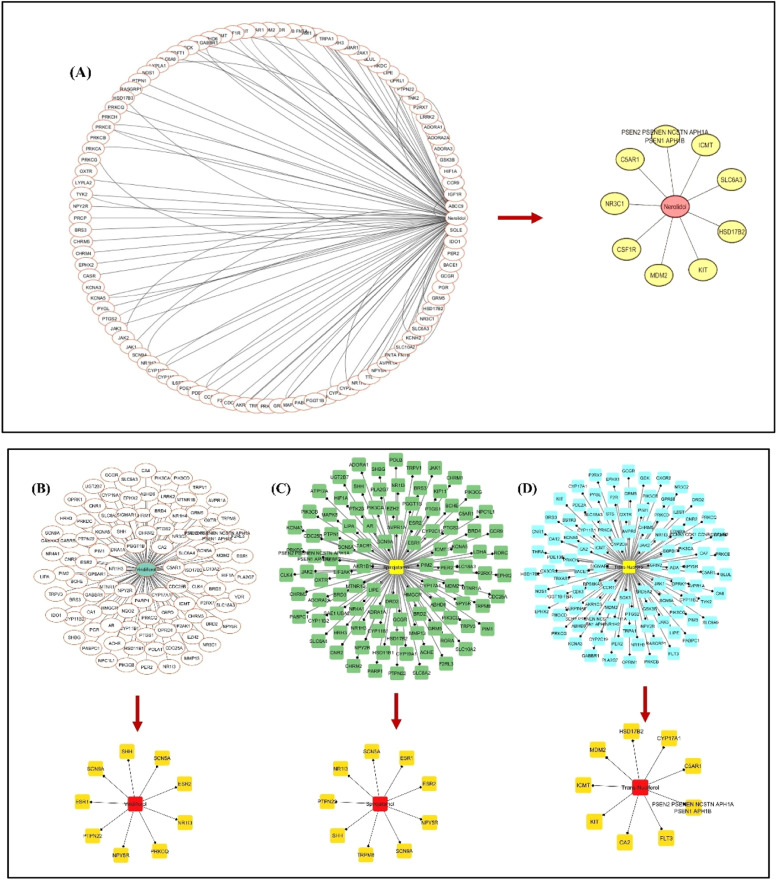
The intersection and core targets of a) Nerolidol b) viridiflorol c) Spirojatamol d) Trans-nuciferol against AD.

Based on the constructed PPI interactions, the PPI network was constructed using Cytoscape (Version 3.10.2) (Fig. 8). The top ten targets were chosen as core targets after the Network Analyzer determined their degree. The top 10 targets were; (FSCN5A, NPY5R, SHH, PTPN22, NR1I3, PRKCQ, ESR1, SCN9A, NCSTN ESR2 for viridiflorol. ICMT, CA2, MDM2, PSEN2, PSENEN, NCSTN, APH1A, PSEN1, APH1B, CYP17A1, HSD17B2, KIT, FLT3, C5AR1 for trans-nuciferol. SCN5A, NPY5R, TRPM8, SHH, PTPN22, NR1I3, ESR1, SCN9A, ESR2, APH1B for Spirojatamol. ICMT, MDM2, PSEN2, PSENEN, NCSTN, APH1A, PSEN1, APH1B, CSF1R, HSD17B2, KIT, NR3C1, C5AR1, SLC6A for Nerolidol. Taken together, the above results imply these molecules possess multidimensional interactions with important AD targets.

### 3.6 GO and KEGG analysis

In this study, 4 compounds were selected from a pool of 43 molecules and successfully mapped to 92 targets associated with AD. The 92 targets were enriched across the top 10 KEGG pathways. Pathways with an adjusted P-value ≤ 0.05 were shortlisted.

**Fig. 9:**
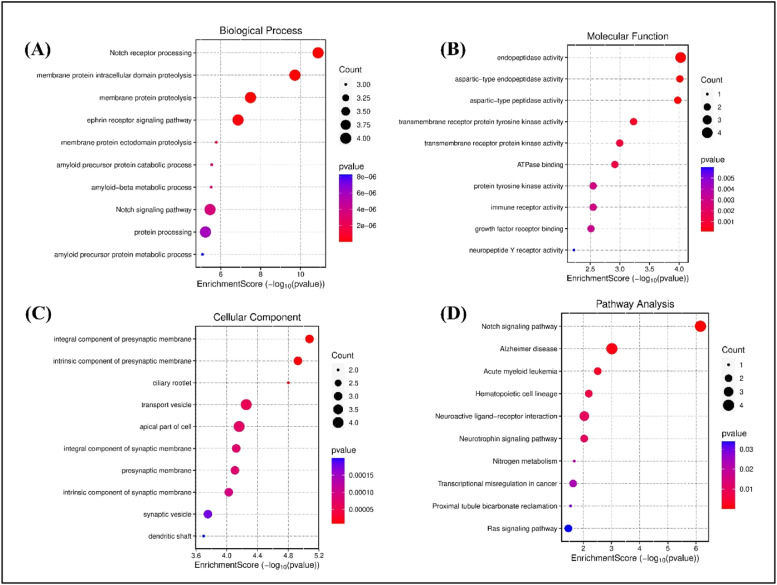
Top 10 GO enrichment analysis a) biological process b) molecular function c) cellular component d) the top 10 enriched KEGG pathways. The node size represents the number of target genes enriched, and the node colour from red to blue represents the p value from large to small.

**Fig. 10:**
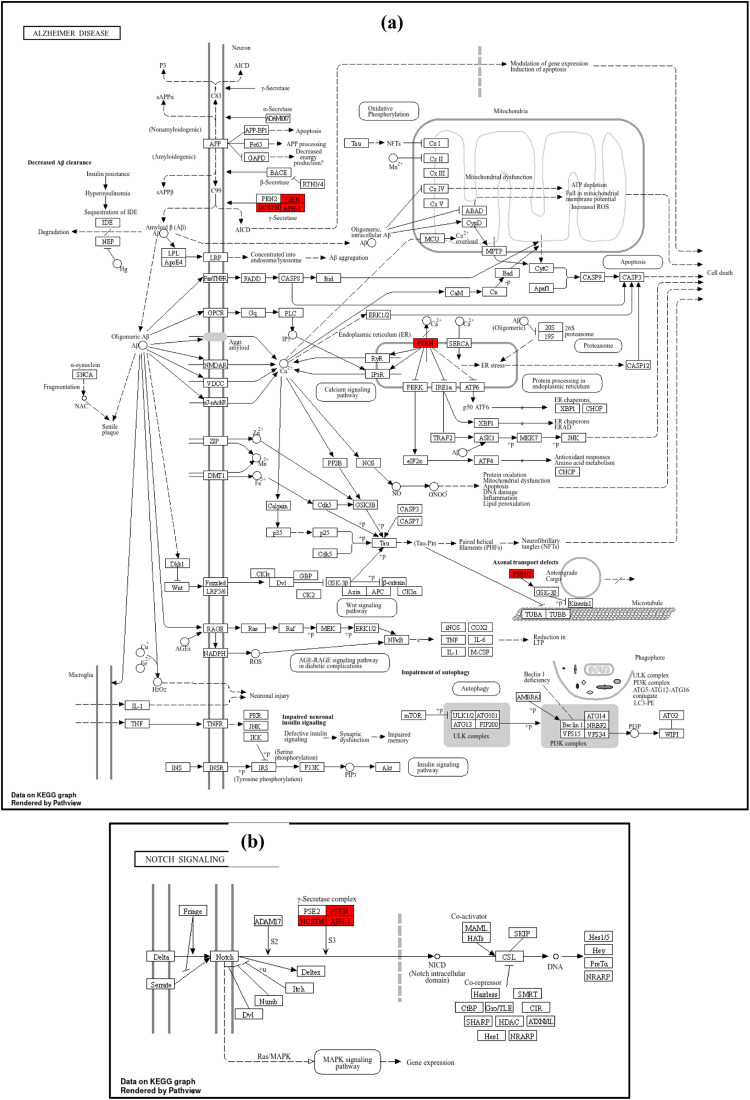

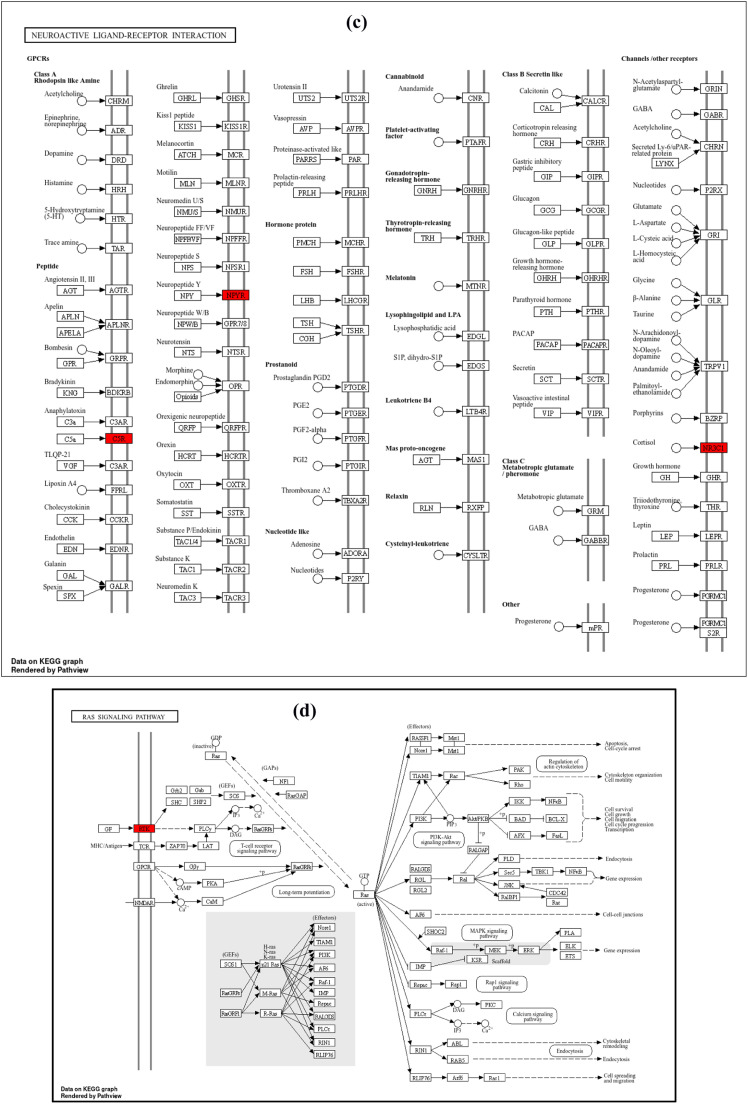

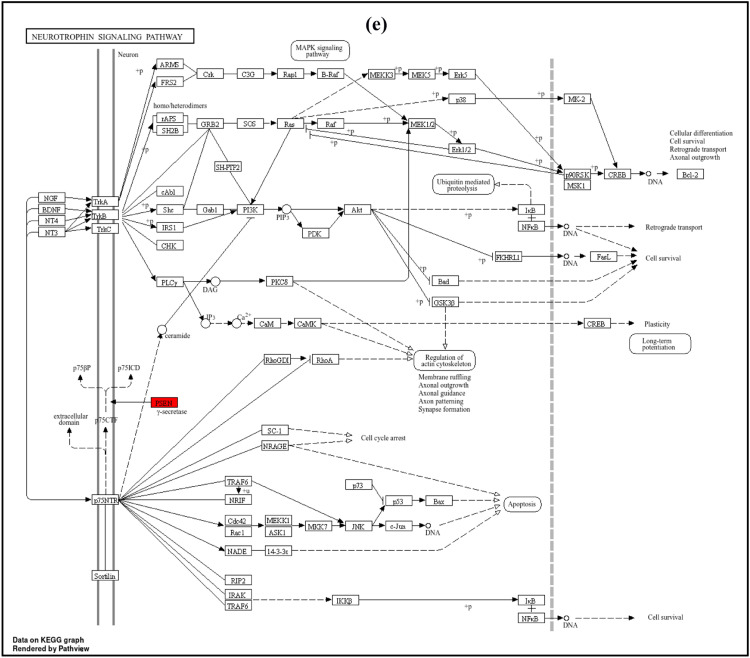
KEGG pathway enrichment analyses of screened compounds in the treatment of AD. a) Alzheimer's disease b) Notch Signaling pathway c) RAS signaling pathway d) Neuroactive ligand receptor interaction pathway e) Neurotrophin signaling pathway.

The GO enrichment analysis for biological processes (BP) highlights the top 10 entries. Among these, the dominant terms include Notch receptor processing (GO:0007220), intracellular domain proteolysis of membrane proteins (GO:0031293), ectodomain proteolysis (GO:0006509), ephrin receptor signaling pathway (GO:0048013), amyloid precursor protein catabolic process (GO:0036503), metabolic processes (GO:0042982), and regulation of amyloid-beta metabolic process (GO:0050435), as shown in Fig. 9a Within the molecular function (MF) categories (Fig. 9b), the dominant terms include endopeptidase activity (GO:0004175), aspartic-type endopeptidase activity (GO:0070001), aspartic-type peptidase activity (GO:0004190), transmembrane receptor protein tyrosine kinase activity (GO:0004714), transmembrane receptor protein kinase activity (GO:0019199), ATPase binding (GO:0051117), protein tyrosine kinase activity (GO:0004713), immune receptor activity (GO:0140375), growth factor receptor binding (GO:0070851), and neuropeptide Y receptor activity (GO:0004984). Within the cellular component (CC) categories (Fig. 9c), key terms include the integral component of the presynaptic membrane (GO:0099056), intrinsic component of the presynaptic membrane (GO:0099057), ciliary rootlet (GO:0035869), transport vesicle (GO:0030133), apical part of the cell (GO:0045177), integral component of the synaptic membrane (GO:0099249), presynaptic membrane (GO:0042734), intrinsic component of the synaptic membrane (GO:0099248), synaptic vesicle (GO:0008021), dendritic shaft (GO:0035633), and exocytic vesicle (GO:0070382). These findings suggest that the selected molecules target various components involved in AD through multiple biological processes, molecular functions, and cellular components. Following KEGG analysis, 32 pathways were found. Fig. 9d highlights the top 10 enriched KEGG pathways. Alzheimer's disease (hsa05010) (Fig. 10a), Notch signaling pathway (hsa04330) (Fig. 10b), leukemia (hsa05221), hematopoietic cell lineage (hsa04640), neurotrophin signaling pathway (hsa04722) (Fig. 10c), neuroactive ligand-receptor interaction (hsa04080) (Fig. 10d), nitrogen metabolism (hsa00910), cancer transcriptional dysregulation (hsa05202), reclamation of proximal tubule bicarbonate (hsa04964), and RAS signaling pathway (hsa04014) (Fig. 10e) are some of these pathways.

**Fig. 11:**
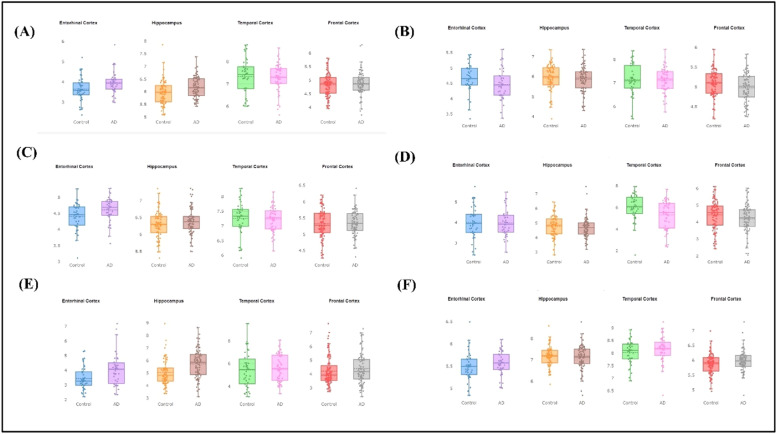
Differential gene expression compared to the control group a) PSEN1 b) PSEN2 c) APH1B d) NPYS1R e) CSAR1 f) NCSTN in the Entorhinal cortex (Control, 39; AD 40). Hippocampus (Control 66; AD 75). Temporal cortex (control 39, AD 53). Frontal cortex (Control 128, AD, 105). Data are presented as means ± SEM.

Using the "Differential Expression" module of the AlzData database, we conducted an in-depth analysis to determine if genes associated with AD pathology show differential expression in AD patients compared to healthy controls (**Supplementary material S2**). Variations in target gene expression levels could potentially influence AD risk. In the entorhinal cortex, we observed that AD patients exhibited significantly lower levels of PSEN2 compared to controls, whereas PSEN1, APH1B, and CSAR1 levels were notably higher . Among the Hippocampus PSEN1 and CSAR1 were upregulated considerably, and a single gene PSEN 2 was significantly downregulated in AD patients compared to controls. CSAR1 was upregulated and PSEN2 was downregulated but only NPYS1R exhibited significant downregulation in the temporal cortex. NCSTN and CSAR1 both showed upregulation, however it was significant for NCSTN. PSEN2 and NPYS1R showed downregulation. These findings suggest that the pathophysiology of AD may be significantly influenced by the use of these compounds Fig. 11.

### 3.7 Molecular docking

**Fig. 12:**
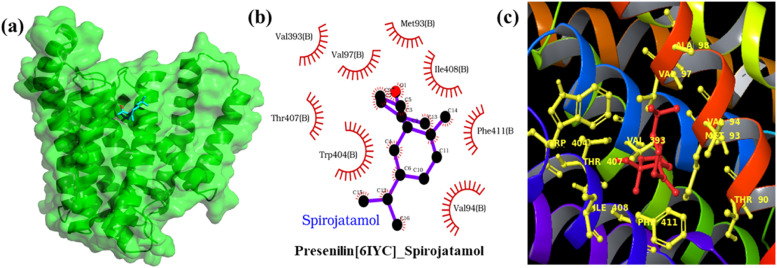
(a) Docking pose of Spirojatamol on Presenilin-1, with the ligand shown in cyan stick representation and the protein in a green cartoon with semi-transparent surface. (b) 2D interaction map of Spirojatamol with Presenilin-1, and (c) 3D interaction map of Spirojatamol with Presenilin-1, highlighting key ligand-protein interactions.

To evaluate the interaction profiles of selected ligands with their targets, we conducted molecular docking and dynamics studies. Given the promiscuous and poorly defined docking sites of the selected targets, blind docking was performed using the Achilles Online Blind Docking server. This server employs a repetitive blind docking approach with the Vina algorithm, yielding more reliable results than a single run. Docking scores (Table 4) indicated that all ligands showed stronger interactions with Presenilin-1 compared to other targets, with Tau and APP also displaying good interactions, particularly with Trans-nuciferol. The Presenilin-1 complex demonstrated the most favorable binding energy of -6.9 kcal/mol with Spirojatamol, primarily attributed to substantial van der Waals interactions (Fig. 12**b and c**). Specifically, Spirojatamol engages in van der Waals interactions with the Met93, Val94, Val393, Trp404, Trp407, Ile408, and Phe411 residues of Presenilin-1. A molecular dynamics simulation of the Presenilin-1 and Spirojatamol complex was conducted to further investigate the interaction behavior and the ligand's impact on the protein.

**Table 4:** Table showing the ligands and their best docking scores obtained from blind docking with each protein and their corresponding PDB IDs of the proteins are also provided.

Protein	PDB ID	Ligand	Best Pose	Docking Score (Kcal/mol)
Presenilin1	6IYC	Spirojatamol	27	-6.9
Presenilin1	6IYC	TransNuciferol	111	-6.8
Presenilin1	6IYC	Viridiflorol	260	-6.4
Presenilin1	6IYC	Nerolidol	104	-6.3
Tau	5O3L	TransNuciferol	304	-6.2
APP	1APP	TransNuciferol	65	-6.1
Tau	5O3L	Nerolidol	43	-6
ApoE	7FCR	TransNuciferol	25	-5.9
APP	1APP	Nerolidol	43	-5.9
APP	1APP	Spirojatamol	84	-5.8
Aβ	2M4J	Viridiflorol	19	-5.7
Tau	5O3L	Viridiflorol	193	-5.7
Aβ	2M4J	TransNuciferol	257	-5.5
ApoE	7FCR	Nerolidol	14	-5.5
ApoE	7FCR	Viridiflorol	25	-5.5
Tau	5O3L	Spirojatamol	625	-5.5
APP	1APP	Viridiflorol	6	-5.4
ApoE	7FCR	Spirojatamol	88	-5.3
APP	2LP1	Viridiflorol	32	-5.3
Aβ	1IYT	TransNuciferol	17	-5.1
APP	2LP1	Spirojatamol	39	-5
APP	2LP1	TransNuciferol	25	-5
Aβ	2M4J	Nerolidol	229	-4.9
Aβ	2M4J	Spirojatamol	160	-4.9
Aβ	1IYT	Spirojatamol	10	-4.7
APP	2LP1	Nerolidol	22	-4.7
Aβ	1IYT	Viridiflorol	14	-4.6
Aβ	1IYT	Nerolidol	-	-

### 3.8 MD simulation

**Fig. 13:**
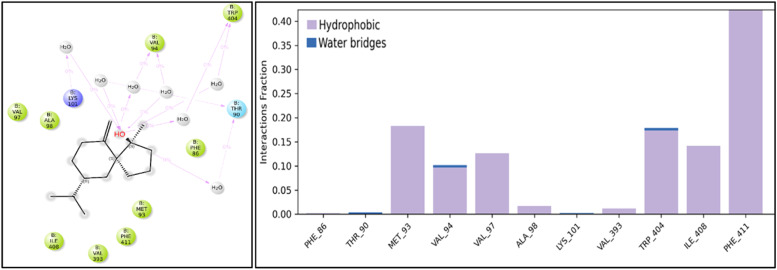
(a) 2D interaction diagram of a target protein with Spirojatamol from the 100 ns molecular simulation, highlighting hydrogen bond interactions facilitated by bridge water molecules. (b) Bar plot illustrating the types of interactions between Spirojatamol and the amino acid residues of chain B in presenilin (PDB Id: 6IYC). The majority of interacting amino acids exhibit hydrophobic interactions, while the remaining residues interact via water bridge molecules. The hydrophobic interacting residues include PHE86, MET93, VAL97, ALA98, VAL393, ILE408, and PHE411. Additionally, THR90 and LYS101, along with two other residues, participate in water bridge interactions. Notably, VAL94 and TRP404 exhibit a combined interaction effect, involving both hydrophobic and water bridge interactions, as observed during a 100 ns molecular simulation.

Molecular dynamics trajectories were visually analyzed using the Maestro interface, revealing that the ligand remained close to the protein throughout the simulation, indicating a strong affinity. We analyzed the interactions between the amino acids of Presenilin-1 and the ligand throughout the 100 ns simulation. Spirojatamol consistently formed water bridges and hydrophobic interactions, primarily involving Phe93, Val97, Trp404, and Ile408 (shown in Fig. 13). A comparative analysis of molecular dynamics (MD) simulations and blind docking revealed that most of these interacting residues were common across both methodologies.

**Fig. 14:**
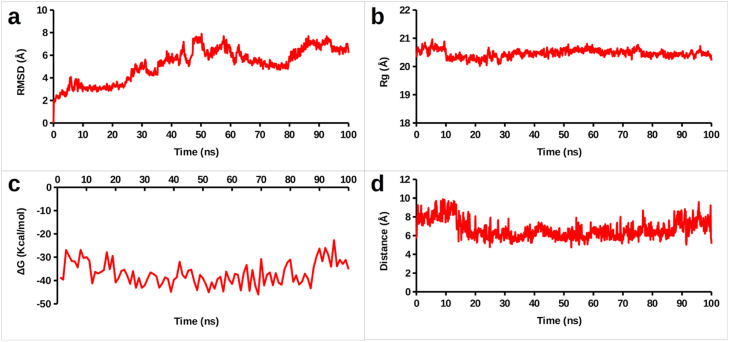
Analysis of Presenilin-1 and Spirojatamol (a) Root mean square deviation (RMSD); (b) Radius of gyration; (c) Binding free energy calculated using MM-GBSA; (d) Distance between protein and ligand.

**Fig. 15:**
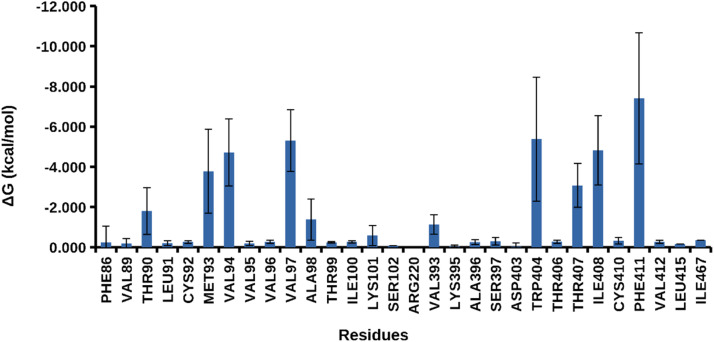
Illustration of binding free energy per amino acid residues.

Additionally, average root mean square deviation (RMSD) and radius of gyration were calculated to assess changes in Presenilin-1, yielding values of 5.19 ± 1.48 Å and 20.46 ± 0.14 Å, respectively (Fig. 14**a and b**). The binding free energy was calculated to monitor energy changes during the trajectory, with an average energy of -36.95 ± 5.00 kcal/mol (Fig. 14c). Per-residue contribution analysis revealed that the primary contributors to the binding free energy were Phe411, Val97, Thr94, Trp404, Ile405, Met93, and Thr407 (Fig. 15). These results align with previously observed interactions from molecular docking and molecular simulation analyses. The distance between the carbonyl carbon of MET93 and the ligand was measured throughout the simulation (Fig. 14d), consistently showing that the ligand remained within 7 Å of the protein. These findings confirm that Spirojatamol exhibits a strong affinity for Presenilin-1.

## 4 Discussion

Since ancient times, plants have been a significant source of traditional medicine for the majority of population. Today, they are also important sources for the novel pharmaceutical molecules [31,32]. The various plant parts e.g., leaves, stem, root, flower, and bulb have been utilized for ages to cure a variety of human ailments. In India use of *N. jatamansi* stretch back to the Vedic era (500–1000 BCE). These accounts are extensively documented in classic ayurvedic texts like Sushruta Samhita, Nighantus Chikitas Granthas, and Charak Samhita [33]. Since past decade lots of studies have confirmed its potential against multiple brain disorders including AD [34].

Number of pathological markers have been identified as important elements in the pathophysiology of AD, including Aβ, h-tau, APOE, AChE, PS1 and PS2 [35]. As a result, current therapies have been developed based on these target factors. The microtubule-associated protein tau, the cholesterol-transporting apolipoprotein E isoform 4 (ApoE4), and the amyloid precursor protein (APP) are the three main pathways involved in the pathophysiology of AD [36]. Familial AD (FAD) is caused by abnormal processing or overexpression of APP, whereas frontotemporal dementia with parkinsonism related to chromosome 17 (FTDP-17) is caused by tau mutations that result in AD-like hyperphosphorylation and neurofibrillary tangles (NFTs) [37,38]. One of the main risk factors for sporadic AD is ApoE4 [39].

*N. jatamansi* has been shown in earlier research to improve memory and reduce cognitive impairments, suggesting that it may be used as a therapy for AD [[40], [41], [42], [43]]. Numerous studies have underscored the pharmacotherapeutic potential of *N. jatamansi* in addressing AD, showcasing its neuroprotective, antioxidant, and memory-enhancing effects. Research by Anupuma et al. demonstrated that jatamansinol, a key bioactive compound derived from N. jatamansi, exhibits multifaceted neuroprotective properties [44]. In a Drosophila model of Alzheimer's disease, jatamansinol mitigates human tau-induced neurotoxicity by reducing reactive oxygen species (ROS) levels and boosting antioxidant enzyme activity in Tau protein-expressing cells. In mammalian models, jatamansinol significantly enhances learning, memory, and locomotion in mice with tau-induced AD. These effects are linked to its ability to inhibit cholinesterase activity, downregulate tau protein expression, and prevent tau-induced ocular degeneration. Joshi et al. investigated the memory-boosting effects of N. jatamansi in both young and aged mice using behavioral models like the elevated plus maze and passive avoidance tests [45]. Mice administered an ethanolic extract of *N. jatamansi* at 200 mg/kg exhibited significant improvements in learning and memory. Additionally, the extract reversed amnesia induced by diazepam and scopolamine, with the latter suggesting enhanced cholinergic neurotransmission as a key mechanism underlying memory restoration. Jatanolides, another class of compounds in *N. jatamansi*, have shown promise in countering dementia by restoring memory, likely through their antioxidant activity. Liu et al. explored the molecular pathways through which N. jatamansi exerts its neuroprotective effects. Using an ethanol extract of N. jatamansi roots, their study highlighted its protective role against amyloid beta (Aβ) toxicity [46]. In vitro experiments with SH-SY5Y cells demonstrated the extract's ability to prevent Aβ-induced cell death. In an Aβ42-expressing Drosophila AD model, the extract reversed neurological deficits and abnormalities. These effects were attributed to its richness in nardosinone and chlorogenic acid, both known for their neuroprotective and anti-inflammatory properties. These findings position *N. jatamansi* as a promising candidate for Alzheimer's treatment. Its diverse mechanisms of action ranging from antioxidant and anti-inflammatory effects to modulation of cholinergic transmission and inhibition of tau pathology, make it a versatile therapeutic agent. Future clinical investigations are warranted to further validate its efficacy and potential application in treating neurodegenerative diseases like AD. Research into the anti-neuroinflammatory properties of *N. jatamansi* has provided valuable insights into its therapeutic potential, particularly in managing inflammation-related neurological disorders. Kim et al. conducted a comprehensive study to evaluate the chemical composition and bioactivity of a 20 % aqueous ethanol extract of N. jatamansi (NJ20) [47]. Their investigation utilized both in vitro and in vivo models of lipopolysaccharide (LPS)-induced inflammation. Through quantitative analysis, they identified desoxo-narchinol A as the predominant compound among the nine secondary metabolites detected in NJ20. Yoon et al. explored a variety of sesquiterpenoids and terpenoids isolated from the rhizomes and roots of *N. jatamansi*, focusing on their anti-neuroinflammatory effects [48]. Among the compounds studied, 7-methoxydesoxo-narchinol, kanshone N, and narchinol A demonstrated significant potential. These compounds exhibited dose-dependent inhibition of LPS-induced NO production in BV2 microglial cells. They reduced the expression of inflammatory mediators such as PGE2, iNOS, COX-2, and cytokines including IL-1β, IL-12, and TNF-α in LPS-stimulated BV2 cells. Pandey et al. investigated the phenolic composition of *N. jatamansi* sourced from high-altitude regions in India [49]. They identified protocatechuic acid and syringic acid as the primary phenolic acids, both known for their potent antioxidant properties. Despite the strong antioxidant activity observed, variability in results suggested the presence of other phenolic derivatives, warranting further research into its full phytochemical profile. Lyle et al. examined the effects of a hydroethanolic extract of *N. jatamansi* (NJE) on stress-induced oxidative damage in Wistar rats [50]. NJE pretreatment significantly reversed stress-induced increases in lipid peroxidation (LPO) and nitric oxide (NO) levels in the brain. Stress-induced suppression of catalase activity was restored with NJE treatment, highlighting its enzymatic antioxidant potential. NJE reduced stress-induced ulcer formation and improved metabolic markers of gastric health. Both doses of 200 mg/kg and 500 mg/kg showed protective effects against chronic fatigue syndrome, with evidence suggesting that NJE modulates the hypothalamic-pituitary-adrenal (HPA) axis to alleviate stress. Sharma et al. assessed the antioxidant potential of *N. jatamansi* rhizome extract using multiple assays. The extract exhibited strong activity in DPPH and superoxide anion scavenging assays, indicating its effectiveness against reactive oxygen species (ROS). Moderate NO scavenging ability was observed, suggesting potential benefits in reducing oxidative stress [51]. The extract also showed significant metal chelation and reducing power activities. Quantification of total phenolic and flavonoid content revealed high levels of bioactive compounds, further supporting its free radical scavenging capabilities. Salim et al. evaluated the neuroprotective efficacy of *N. jatamansi* (NJ) in a model of acute ischemic stroke induced by middle cerebral artery (MCA) occlusion in rats [52]. The study reported the following outcomes after administering NJ (250 mg/kg) for 15 days post-occlusion improved neurobehavioral functions, oxidative stress modulation, restoration of cellular functions. Another study aimed to investigate the therapeutic effects of nerolidol in an amyloid-beta (Aβ)-induced AD rat model [53]. Treatment with nerolidol or donepezil ameliorated these effects, reducing Aβ plaque accumulation and enhancing BDNF and CREB-1 expression. Furthermore, nerolidol administration prior to Aβ exposure proved more effective, suggesting its potential as a preventive strategy against AD. These findings indicate that nerolidol offers neuroprotection via modulation of BDNF-CREB-1 signaling and restoration of cholinergic function, positioning it as a promising therapeutic approach for AD. Furthermore, nerolidol attenuates neuroinflammation through inhibition of TLR-4/NF-κB and COX-2/NF-κB pathways [54], and modulates BDNF/TrkB/CREB signaling to promote neuronal health. These studies highlight *N. jatamansi* as a powerful neuroprotective agent with the potential to mitigate cognitive decline and ischemic damage. Its ability to reduce oxidative stress, restore antioxidant defences, and protect neuronal integrity makes it a promising candidate for therapeutic interventions in conditions such as AD and cerebral ischemia.

Thus, in our study we used multidimensional in silico techniques to investigate *N. jatamansi* anti-Alzheimer potential, which revealed that a number of bioactive chemicals may target multiple genes and pathways to provide neuroprotective benefits (**Supplementary material S3**). In particular, we investigated four important bioactive compounds that are sesquiterpenes: nerolidol, viridiflorol, spirojatamol, and trans-nuciferol. These bioactive molecules target 28 genes linked to AD. Their pharmacokinetic profiling demonstrated a potent capacity to cross the blood-brain barrier (BBB), indicating that *Nardostachys jatamansi* may have anti-Alzheimer benefits as a result of direct brain action. Our study also demonstrated that the neuroprotective properties of Viridiflorol, Spirojatamol, and Trans-nuciferol, as well as the anti-Alzheimer impact of Nerolidol via its interaction with Aβ, APP, Tau, APOE and PS1. These results imply that a *Nardostachys jatamansi,* which contains these bioactive chemicals, may be useful in the treatment of AD.

Potential modes of action for NJ in AD were identified by Gene Ontology (GO) enrichment analysis. It mainly targeted the control of Notch receptor processing intracellular domain proteolysis of membrane proteins, amyloid precursor protein catabolic process and regulation of amyloid-beta metabolic process. AD is strongly affected by amyloid-beta (Aβ) and tau proteins, which disrupt important molecular pathways needed for memory and synaptic plasticity [55]. These disruptions happen at both pre- and post-synaptic sites, where Aβ and tau interfere with the activity of a key protein, CREB (cAMP response element-binding protein), which is essential for memory through its phosphorylation at Ser133 [56]. Aβ prevents the normal increase in CREB phosphorylation during long-term potentiation (LTP), which leads to reduced activity in the NO/cGMP/PKG and cAMP/PKA pathways—two main pathways that control CREB [57]. Similarly, too much tau protein and its hyperphosphorylation also lower CREB phosphorylation at Ser133, partly due to reduced NR2B phosphorylation. These effects on CREB directly contribute to the synaptic and memory impairments seen in AD, and such findings have been validated in human studies, where decreased levels of CREB and phospho-CREB were observed in the hippocampus of AD patients [58]. In addition to affecting molecular pathways, amyloid-beta (Aβ) and tau proteins also lower the levels of brain-derived neurotrophic factor (BDNF), which is essential for neuron survival, synaptic plasticity, and memory [59]. Lower BDNF levels in the blood and brains of AD patients are associated with cognitive decline. This decrease in BDNF is linked to both Aβ-related impairments in long-term potentiation (LTP) and tau-related issues in AD models [60]. These findings highlight the multifaceted role of Aβ and tau in undermining memory and synaptic function, contributing to the progressive cognitive decline in AD.

At the genetic level, AD is influenced by both familial and sporadic forms. Familial AD is often caused by pathogenic variants in the presenilin 1 (PSEN1) and presenilin 2 (PSEN2) genes, which are integral to the γ-secretase complex responsible for cleaving amyloid precursor protein (APP) and generating Aβ [61]. Recent research has shown that alternative splicing of PSEN1 and PSEN2 is a feature of sporadic AD, resulting in the production of truncated PSEN2 proteins in affected individuals [62]. This contrasts with familial AD, where full-length but variant PSEN1 and PSEN2 proteins contribute to disease onset [63]. These splicing patterns provide new insights into the differential mechanisms of AD progression in familial versus sporadic cases.

Nicastrin (NCSTN), another component of the γ-secretase complex, also plays a critical role in the pathogenesis of AD by facilitating the production of Aβ [64]. Although a genetic association between NCSTN variants and AD has not been definitively established, its biological relevance suggests that further investigation is necessary. Interestingly, recent studies highlight the potential of targeting other receptor systems, such as GALR2 and NPY1R, to enhance memory and promote neuronal survival [65]. The co-administration of agonists for these receptors has shown promise in animal models, suggesting a novel therapeutic strategy for addressing cognitive deficits and promoting neurogenesis in AD [66,67]. Finally, the APH1B gene, which encodes a subunit of γ-secretase, has been linked to the production of longer, more pathogenic Aβ peptides, particularly those with a higher Aβ1–42/1–40 ratio [68]. This shift in peptide production may exacerbate AD pathology. Elevated APH1B expression in the blood of AD patients has also been associated with deficits in the insulin-like growth factor 1 (IGF-1) signaling pathway, which is known to play a role in neuroinflammation, tau phosphorylation, and Aβ accumulation [69]. This connection suggests a broader systemic involvement of growth hormone signaling in AD, further complicating its molecular landscape.

In summary, AD is a complex neurodegenerative disorder driven by the interplay of Aβ, tau, APP and various genetic and signaling factors. The disruption of key molecules along with altered gene expression and receptor signaling, underpins the progressive cognitive decline characteristic of the disease. Ongoing research into these pathways offers potential avenues for therapeutic intervention, particularly in modulating the activity of γ-secretase components and receptor systems that affect synaptic plasticity and neurogenesis. Understanding these intricate molecular interactions will be essential for developing effective treatments for AD. Drugs that target the Renin-Angiotensin System (RAS), commonly used to treat high blood pressure, have shown promise in slowing down AD [70]. The ACE1/Ang II/AT1R axis is thought to be more active, leading to increased oxidative stress, inflammation, blood-brain barrier problems, and reduced brain blood flow in AD [71]. Studies have found that people taking angiotensin receptor blockers (ARBs) and angiotensin-converting enzyme inhibitors (ACEIs) may have a lower risk of developing AD, suggesting these drugs could help in AD treatment [72]. Neurotrophins, such as NGF and BDNF, are vital proteins involved in neuronal survival and function, particularly in the hippocampus and cholinergic basal forebrain neurons [73]. NGF binds to TrkA receptors on cholinergic neurons, supporting synaptic connections with the hippocampus and cortex, while BDNF is crucial for synaptic plasticity and memory formation through its action on TrkB receptors [74]. Disruptions in the expression of NGF, BDNF, or their receptors can impair memory and contribute to neurodegeneration in AD. These alterations occur early in the disease process, highlighting the potential for neurotrophin-based therapies in AD [75]. Additionally, neuroactive ligand-receptor interaction signaling, essential for neuronal communication, may also play a role in AD [76]. Disruptions in this pathway can lead to memory deficits and cognitive impairment. These findings suggest that disturbances in neuroactive signaling may exacerbate AD pathogenesis. In summary, the altered expression of Notch1, the dysregulation of the RAS system, and disruptions in neurotrophin and neuroactive ligand-receptor pathways all appear to contribute to the development and progression of AD. These pathways represent potential therapeutic targets, offering new avenues for intervention in the treatment of AD. These results suggest that N. jatamansi modulate several signaling pathways to produce its anti-Alzheimer's effects via significant interactions among target proteins and ligand molecules as shown by molecular docking and molecular dynamic simulation.

## 5 Conclusion

Our findings suggest *N. jatamansi* phytoconstituents can influence key signaling pathways involved in Alzheimer's disease. Computational analysis reveals a strong association between the studied molecules and their potential roles in AD prevention. Their effective binding to key targets (such as Aβ, APP, Tau, APOE, and PS1) implicated in AD pathogenesis makes them promising candidates for further study and development. However, additional in vitro and in vivo studies are necessary to confirm their therapeutic potential and translate these findings into clinical applications for AD management. The primary compounds identified in the study were spirojatamol, viridiflorol, nerolidol, and trans-Nuciferol, with key genes including PSEN1, PSEN2, APH1B, NPYS1R, CSAR1, and NCSTN. Further molecular docking and molecular dynamics simulations revealed that spirojatamol and PS1 showed the most favorable results due to low binding energy and high structural stability for AD treatment. This research provides a theoretical foundation for a deeper understanding of the pharmacological effects of *N. jatamansi* in treating AD and elucidates its mechanism of action.

## Ethical statement

This study does not involve human or animal models

## Funding

This project did not receive any specific grant from funding agencies in the public, commercial, or not-for profit sectors.

## CRediT authorship contribution statement

**Abdul Jalil Shah:** Conceptualization, Writing – original draft, Investigation, Formal analysis. **Mohammad Younis Dar:** Conceptualization, Formal analysis, Writing – review & editing. **Mohd Adnan:** Conceptualization. **Tanmaykumar Varma:** Software, Visualization, Data curation. **Dhairiya Agarwal:** Software, Visualization, Data curation. **Prabha Garg:** Software, Data curation, Writing – review & editing. **Reyaz Hassan Mir:** Writing – review & editing. **Rampratap Meena:** Formal analysis, Writing – review & editing. **Mubashir Hussain Masoodi:** Supervision, Conceptualization, Writing – review & editing.

## Declaration of competing interest

The authors declare that they have no known competing financial interests or personal relationships that could have appeared to influence the work reported in this paper.

## Data Availability

Data will be made available on request.

## Appendix Supplementary materials

Image, application 1

Image, application 2

Image, application 3
