# Supplementary material for: Integration of phytochemical profiling and computational approaches to evaluate the neuroprotective potential of Nardostachys jatamansi in Alzheimer's disease
Source: Biotechnol Rep (Amst). 2025 Feb 8;45:e00881. doi: 10.1016/j.btre.2025.e00881 (PMC11872466; doi:10.1016/j.btre.2025.e00881)

***Supplementary information 3***

**Integration of Phytochemical Profiling and Computational Approaches to Evaluate the Neuroprotective Potential of *Nardostachys jatamansi* in Alzheimer’s Disease**

Abdul Jalil Shah^a*^, Mohammad Younis Dar^b^, Mohd Adnan^c^, Tanmaykumar Varma^d^, Dhairiya Agarwal^d^, Prabha Garg^d^, Reyaz Hassan Mir^a^, Rampratap Meena^e^, Mubashir Hussain Masoodi ^a*^

^a^Pharmaceutical Chemistry Division, Department of Pharmaceutical Sciences, University of Kashmir, Hazratbal, Srinagar-190006, Jammu and Kashmir, India.

^b^Drug Standardization Research Unit, Regional Research Institute of Unani medicine (CCRUM), Naseem Bagh campus, University of Kashmir, Srinagar, Jammu and Kashmir, India. 190006.

^c^Department of Biology, College of Science, University of Ha’il, Ha’il, P.O. Box 2440, Saudi Arabia

^d^National Institute of Pharmaceutical Education and Research, S.A.S. Nagar Mohali-160062, Punjab India

^e^Central Council for Research in Unani medicine (CCRUM), 61-65, opp. D-Block, Institutional Area, Janakpuri, New Delhi,110058, India.

***Correspondence**

Prof. Mubashir Hussain Masoodi & Abdul Jalil Shah

Pharmaceutical Chemistry Division, Department of Pharmaceutical Sciences, University of Kashmir, Hazratbal, Srinagar-190006, Kashmir, India.

**Email:** [**mubashir@kashmiruniversity.ac.in , shahwyl@gmail.com**](mailto:mubashir@kashmiruniversity.ac.in%20%20shahwyl@gmail.com)

**Fig.S1:** Cnet plot showed the relationship between the DEGs and KEGG pathways. Category = the enriched KEGG pathway. Size = the number of differentially expressed genes which belong to the enriched KEGG pathway. Fold change = the fold change difference


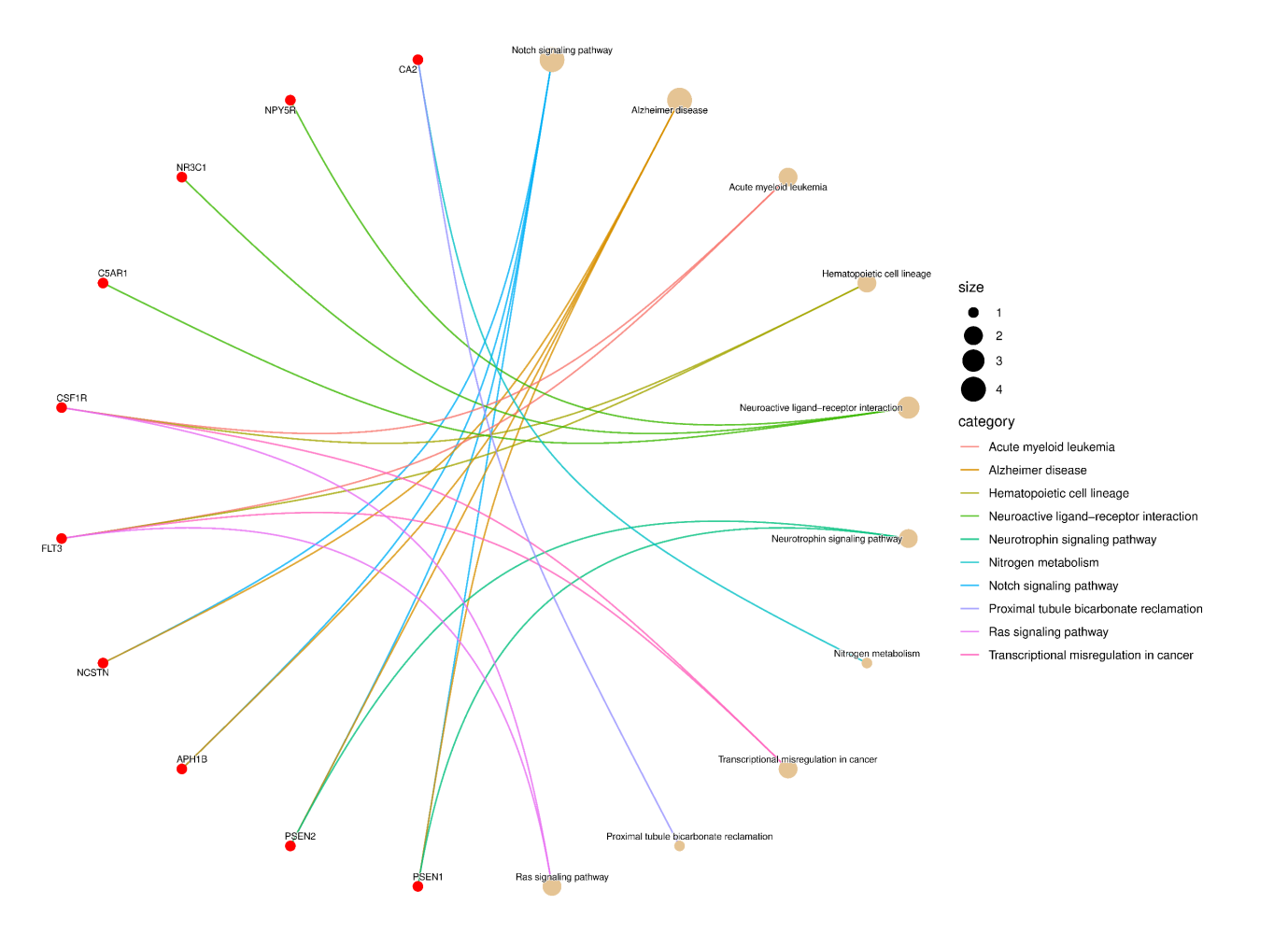

Supplement: Supplementary file 1 [file mmc1.docx]
