# Supplementary material for: Integration of phytochemical profiling and computational approaches to evaluate the neuroprotective potential of Nardostachys jatamansi in Alzheimer's disease
Source: Biotechnol Rep (Amst). 2025 Feb 8;45:e00881. doi: 10.1016/j.btre.2025.e00881 (PMC11872466; doi:10.1016/j.btre.2025.e00881)
Supplement: Supplementary file 2 [file mmc2.pdf]

Supplementary information 1

Integration of Phytochemical Profiling and Computational Approaches to Evaluate the Neuroprotective Potential of *Nardostachys jatamansi* in Alzheimer's Disease

Abdul Jalil Shah[a*], Mohammad Younis Dar[b], Mohd Adnan[c], Tanmaykumar Varma[d], Dhairiya Agarwal[d], Prabha Garg[d], Reyaz Hassan Mir[a], Rampratap Meena[e], Mubashir Hussain Masoodi [a*]

[a]Pharmaceutical Chemistry Division, Department of Pharmaceutical Sciences, University of Kashmir, Hazratbal, Srinagar-190006, Jammu and Kashmir, India.

[b]Drug Standardization Research Unit, Regional Research Institute of Unani medicine (CCRUM), Naseem Bagh campus, University of Kashmir, Srinagar, Jammu and Kashmir, India. 190006.

[c]Department of Biology, College of Science, University of Ha'il, Ha'il, P.O. Box 2440, Saudi Arabia

[d]National Institute of Pharmaceutical Education and Research, S.A.S. Nagar Mohali-160062, Punjab India

[e]Central Council for Research in Unani medicine (CCRUM), 61-65, opp. D-Block, Institutional Area, Janakpuri, New Delhi,110058, India.

***Correspondence**

Prof. Mubashir Hussain Masoodi & Abdul Jalil Shah

Pharmaceutical Chemistry Division, Department of Pharmaceutical Sciences, University of Kashmir, Hazratbal, Srinagar-190006, Kashmir, India.

Email: mubashir@kashmiruniversity.ac.in shahwyl@gmail.com

GC/MS library of *N.jatamansi* rhizome using Pet. ether extract

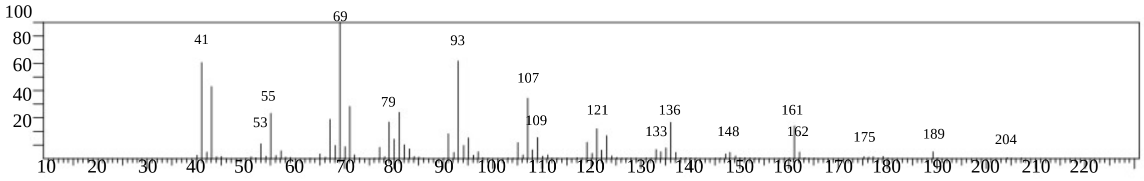

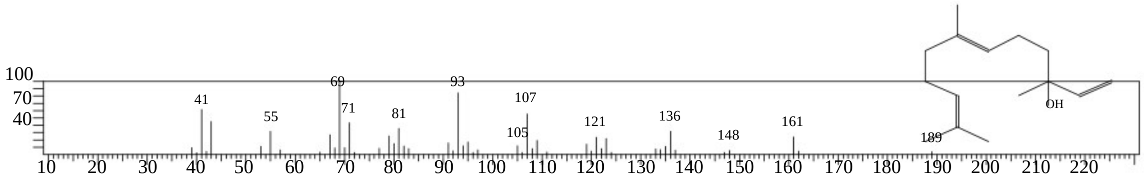

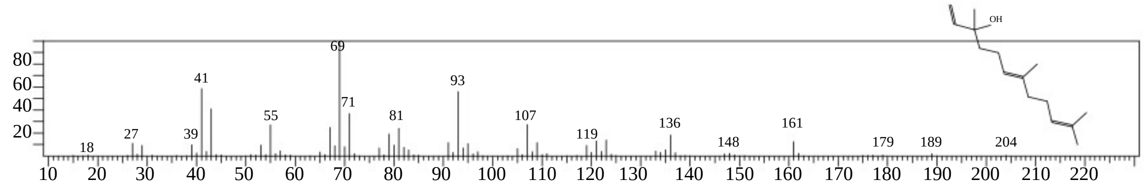

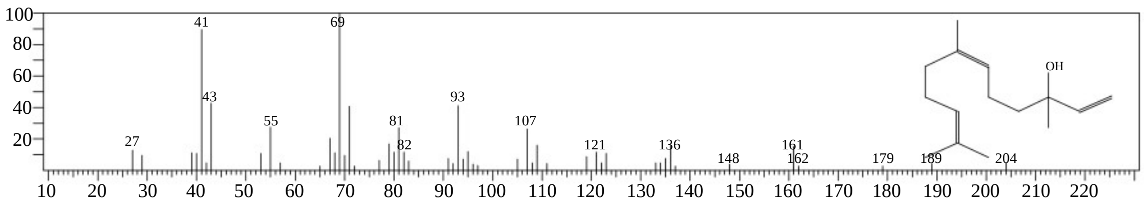

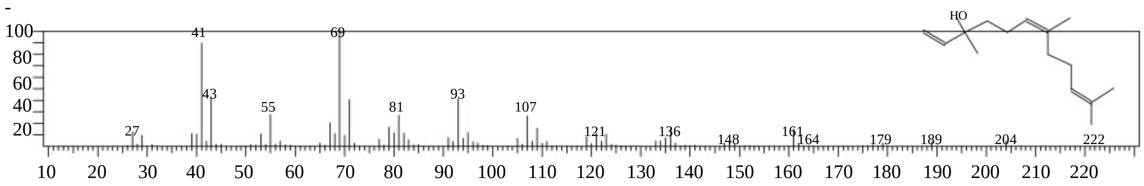

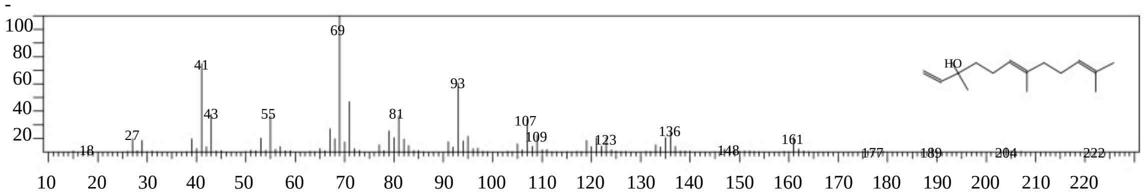

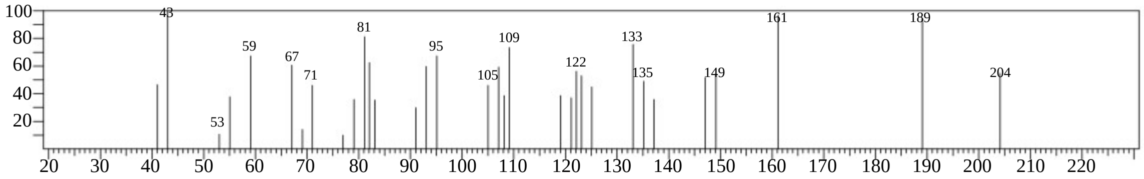
43
53
59
67
71
81
95
105
109
122
133
135
149
161
189
204

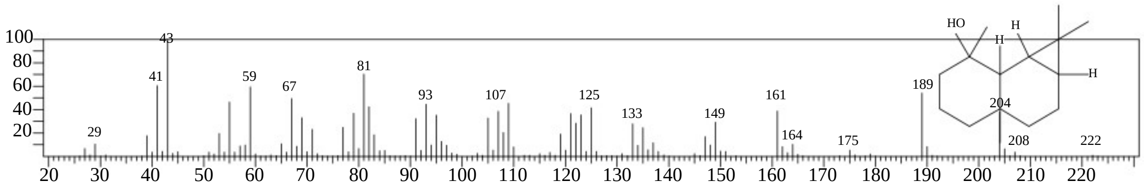
29
41
43
59
67
81
93
107
125
133
149
161
164
175
189
204
208
222
HO
H
H
H

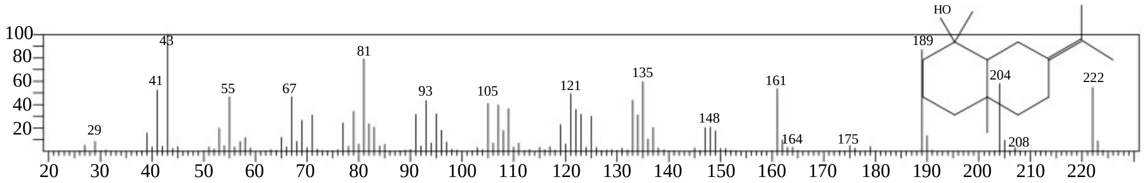
29
41
43
55
67
81
93
105
121
135
148
161
164
175
189
204
208
222
HO

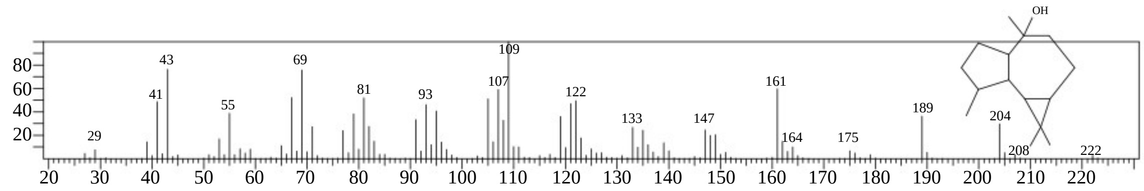
29
41
43
55
69
81
93
107
109
122
133
147
161
164
175
189
204
208
222
OH

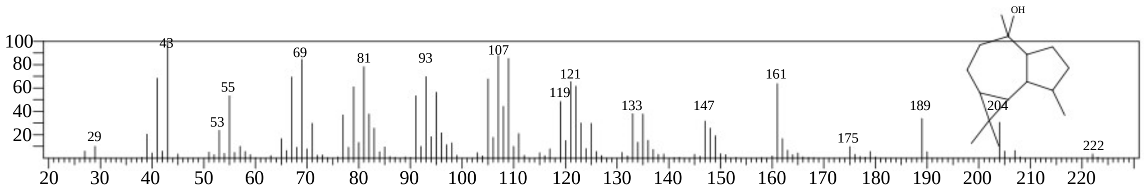
29
43
53
55
69
81
93
107
119
121
133
147
161
175
189
204
222
OH

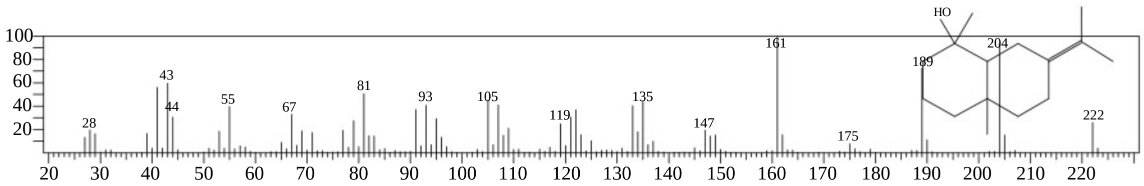
28
43
44
55
67
81
93
105
119
135
147
161
175
189
204
222
HO

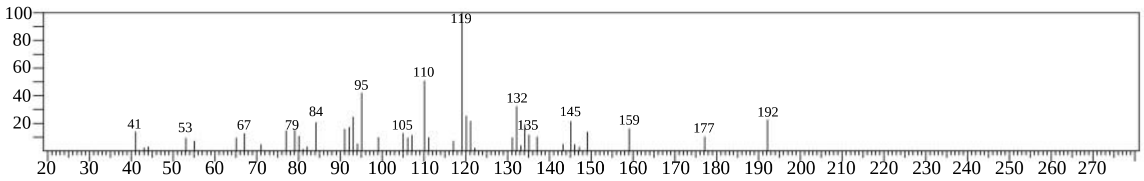
41 53 67 79 84 95 105 110 119 132 135 145 159 177 192

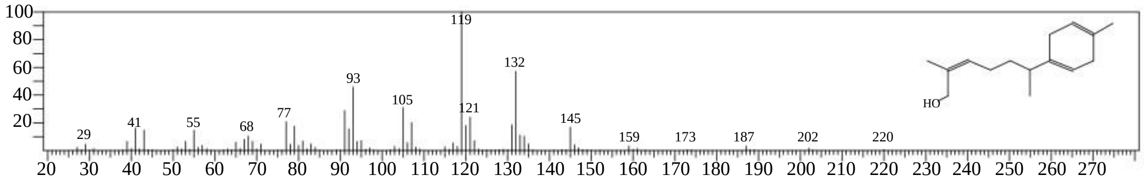
29 41 55 68 77 93 105 119 121 132 145 159 173 187 202 220
HO

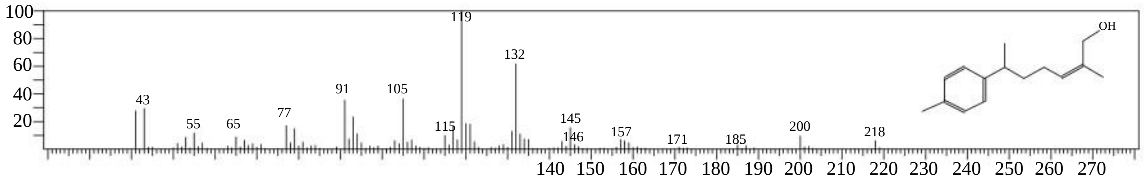
43 55 65 77 91 105 115 119 132 145 146 157 171 185 200 218
OH

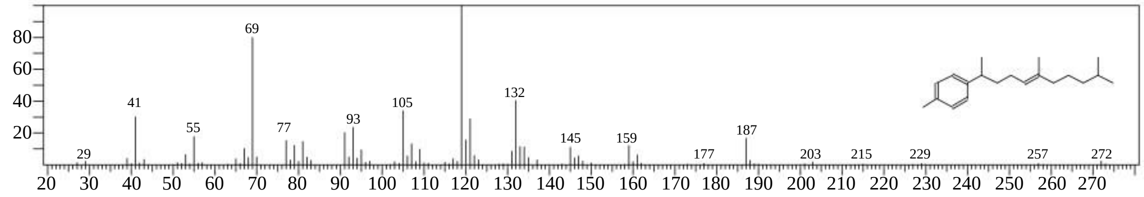
29 41 55 69 77 93 105 132 145 159 177 187 203 215 229 257 272

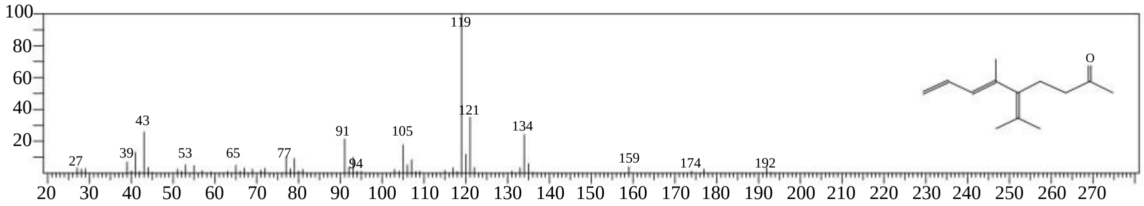
27 39 43 53 65 77 91 94 105 119 121 134 159 174 192
O

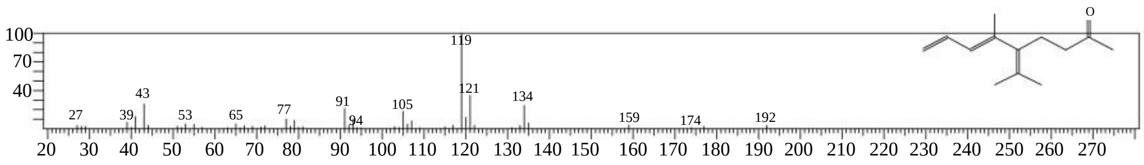
27 39 43 53 65 77 91 94 105 119 121 134 159 174 192
O

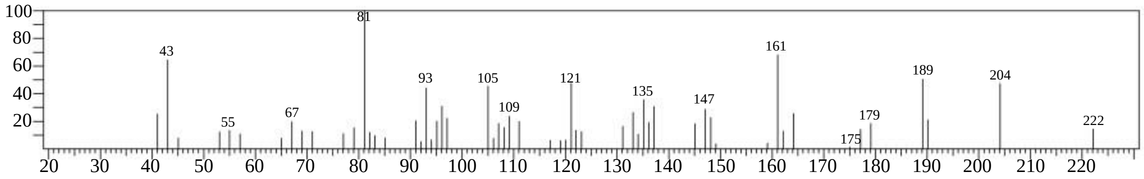
81
43
93
105
121
161
189
204
55
67
109
135
147
179
175
222

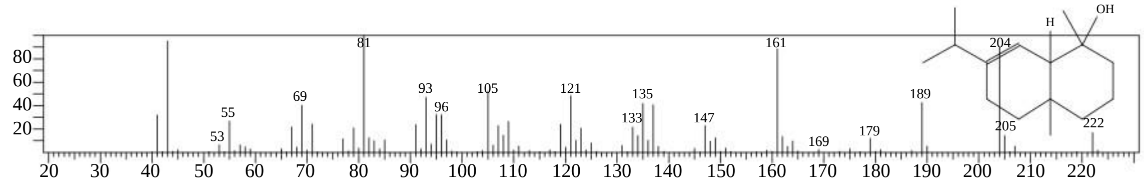
OH
H
81
161
204
93
96
105
121
135
55
69
53
133
147
189
169
179
205
222

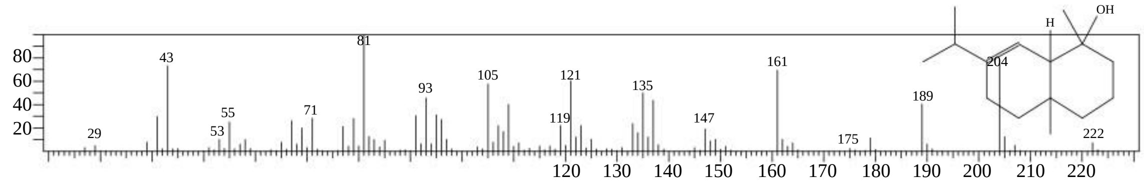
OH
H
81
43
161
204
93
105
121
135
55
71
53
29
119
147
189
175
222

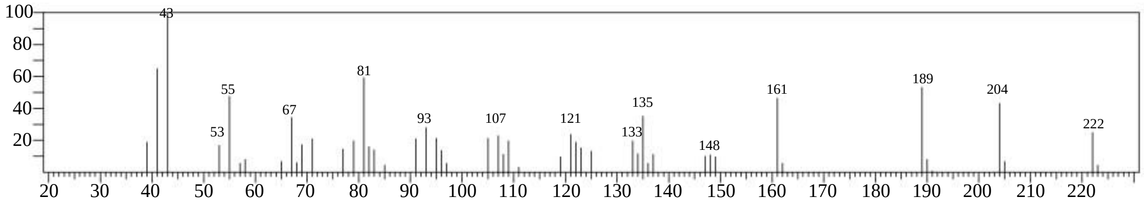
43
81
55
67
53
93
107
121
133
135
148
161
189
204
222

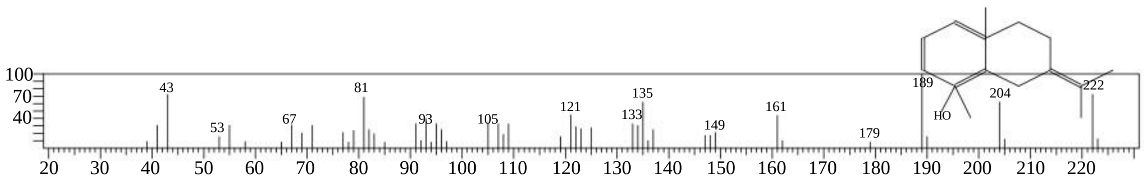
HO
43
81
53
67
93
105
121
133
135
149
161
179
189
204
222

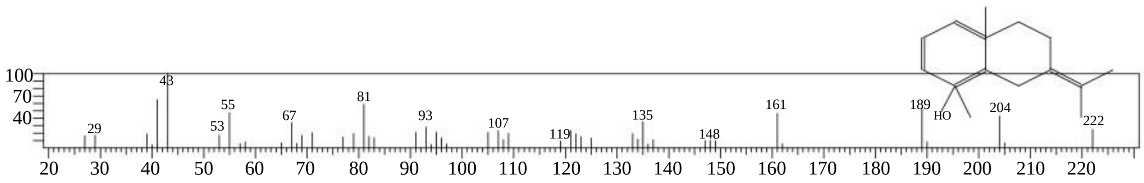
HO
43
29
53
55
67
81
93
107
119
135
148
161
189
204
222

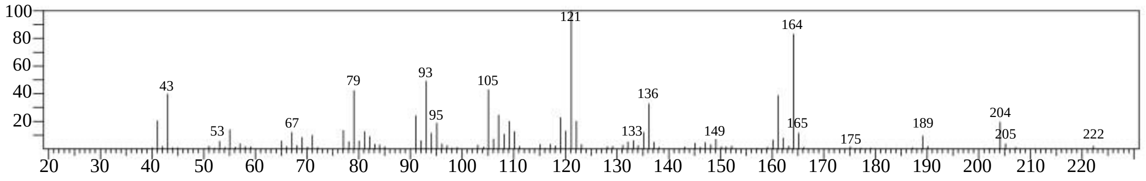
43
53
67
79
93
95
105
121
133
136
149
164
165
175
189
204
205
222

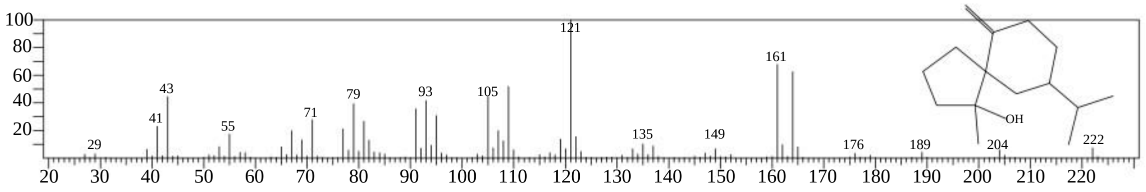
29
41
43
55
71
79
93
105
121
135
149
161
176
189
204
222
OH

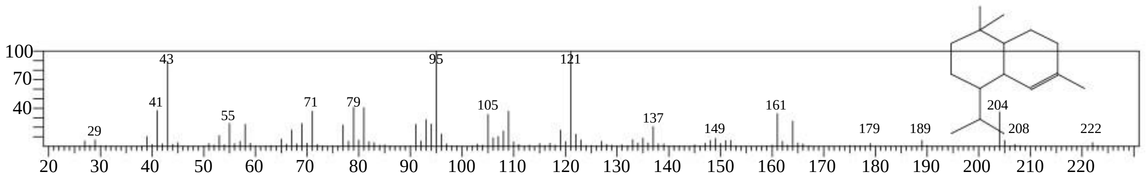
29
41
43
55
71
79
95
105
121
137
149
161
179
189
204
208
222

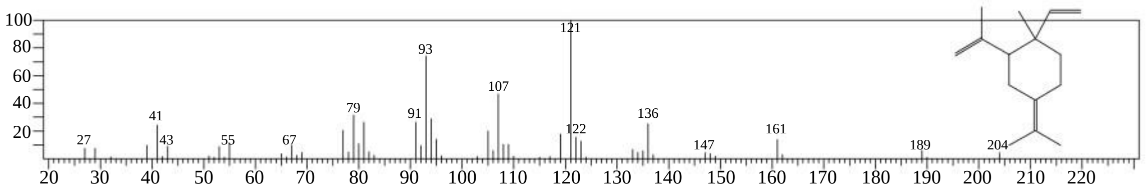
27
41
43
55
67
79
91
93
107
121
122
136
147
161
189
204

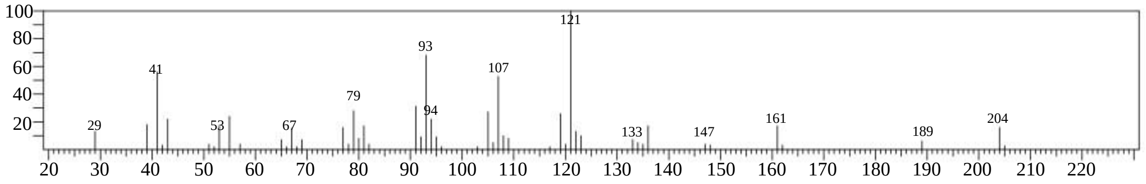
29
41
53
67
79
93
94
107
121
133
147
161
189
204

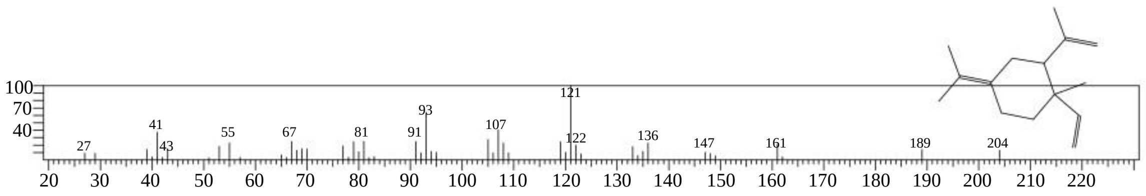
27
41
43
55
67
81
91
93
107
121
122
136
147
161
189
204

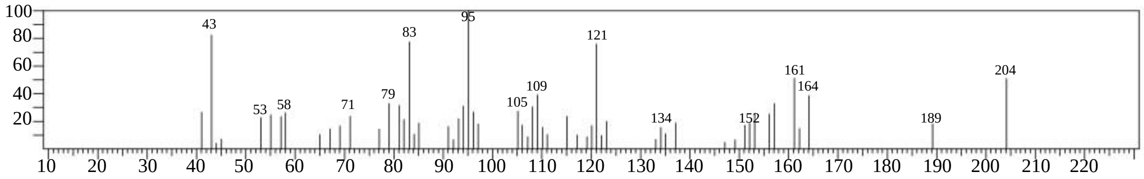
43
53
58
71
79
83
95
105
109
121
134
152
161
164
189
204

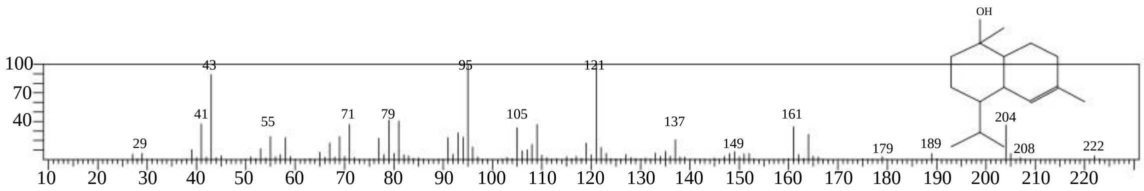
OH
29
41
43
55
71
79
95
105
121
137
149
161
179
189
204
208
222

OL

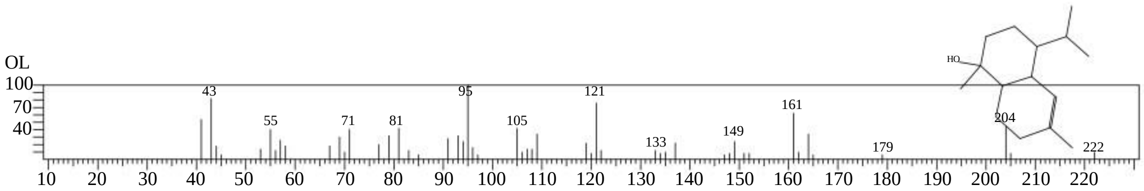
HO
43
55
71
81
95
105
121
133
149
161
179
204
222

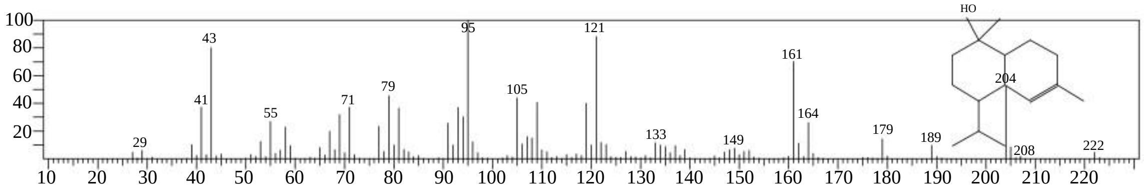
HO
29
41
43
55
71
79
95
105
121
133
149
161
164
179
189
204
208
222

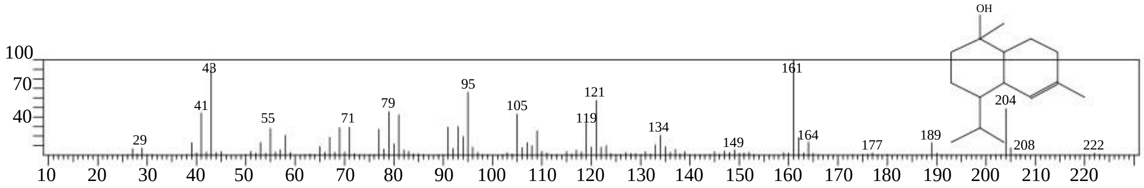
OH
29
41
43
55
71
79
95
105
119
121
134
149
161
164
177
189
204
208
222

OL

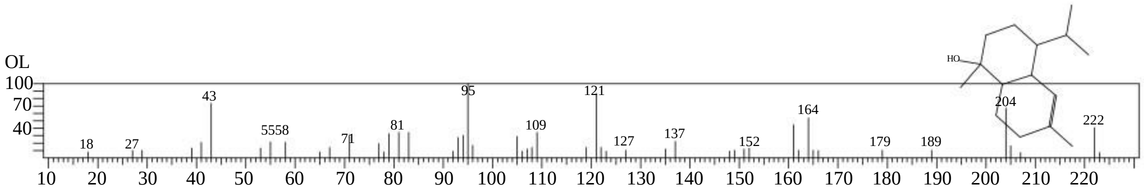
HO
18
27
43
5558
71
81
95
109
121
127
137
152
164
179
189
204
222

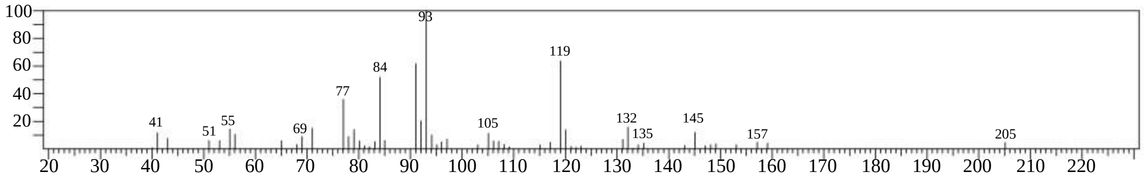
100 80 60 40 20
93 119 84 77 41 51 55 69 105 132 135 145 157 205
20 30 40 50 60 70 80 90 100 110 120 130 140 150 160 170 180 190 200 210 220

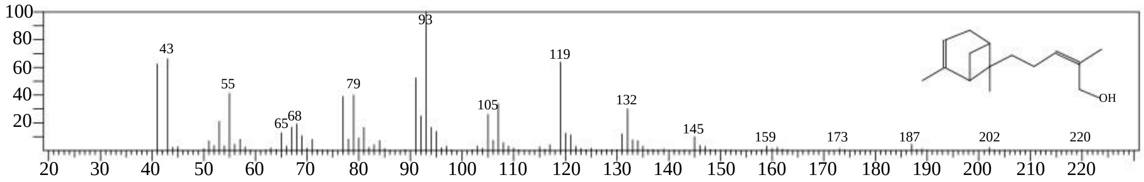
100 80 60 40 20
93 43 119 55 79 105 132 65 68 145 159 173 187 202 220
OH
20 30 40 50 60 70 80 90 100 110 120 130 140 150 160 170 180 190 200 210 220

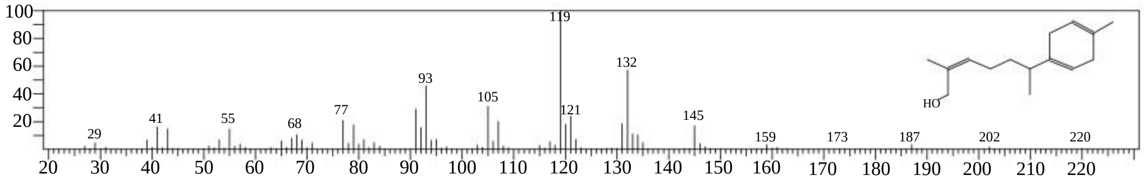
100 80 60 40 20
119 132 93 105 77 121 41 55 68 145 29 159 173 187 202 220
HO
20 30 40 50 60 70 80 90 100 110 120 130 140 150 160 170 180 190 200 210 220

:

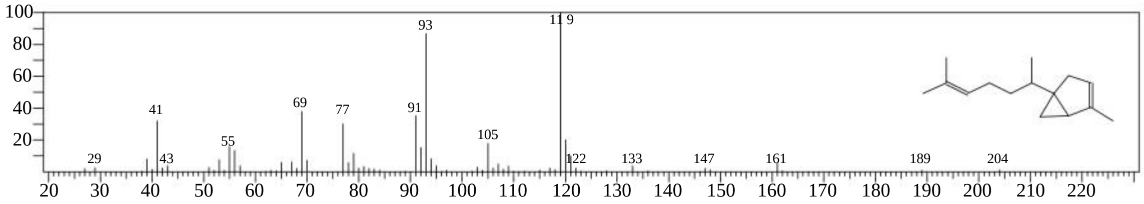
100 80 60 40 20
11 9 93 41 69 77 91 105 55 29 43 122 133 147 161 189 204
20 30 40 50 60 70 80 90 100 110 120 130 140 150 160 170 180 190 200 210 220

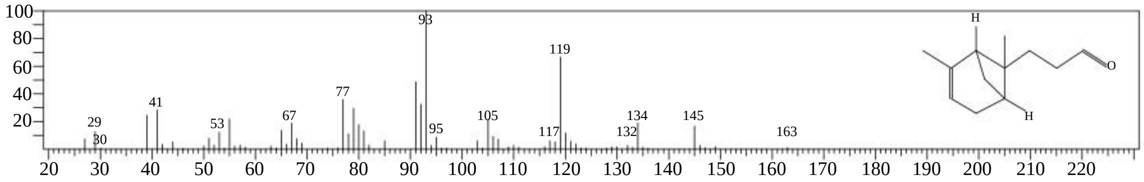
100 80 60 40 20
93 119 77 41 67 105 134 145 29 53 95 117 132 163 30
H
O
H
20 30 40 50 60 70 80 90 100 110 120 130 140 150 160 170 180 190 200 210 220

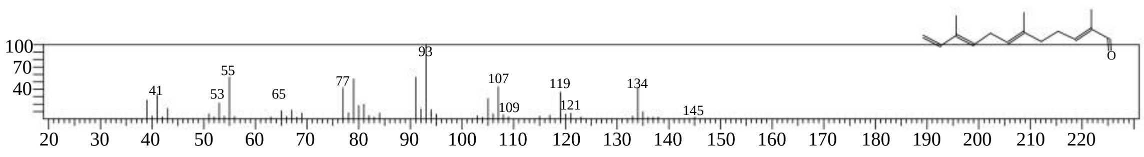
100 70 40
93 55 107 77 119 134 41 53 65 109 121 145
O
20 30 40 50 60 70 80 90 100 110 120 130 140 150 160 170 180 190 200 210 220

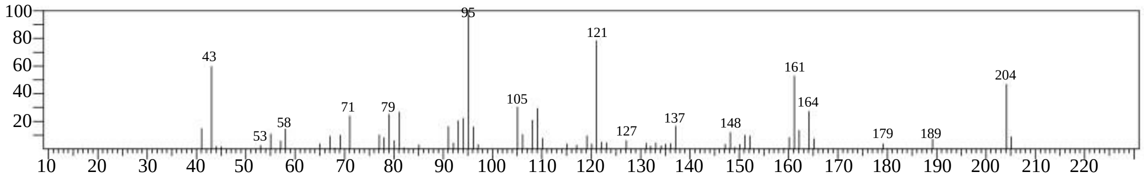

100
80
60
40
20
43
53
58
71
79
95
105
121
127
137
148
161
164
179
189
204
10 20 30 40 50 60 70 80 90 100 110 120 130 140 150 160 170 180 190 200 210 220

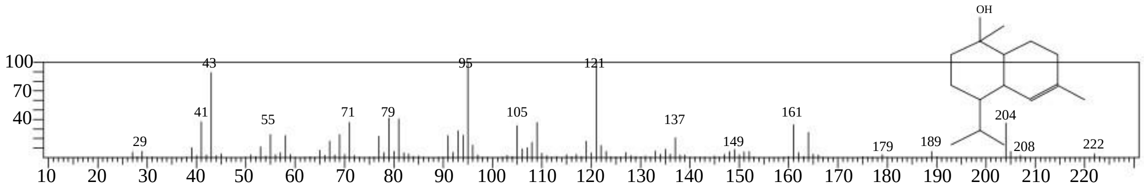

OH
100
70
40
29
41
43
55
71
79
95
105
121
137
149
161
179
189
204
208
222
10 20 30 40 50 60 70 80 90 100 110 120 130 140 150 160 170 180 190 200 210 220

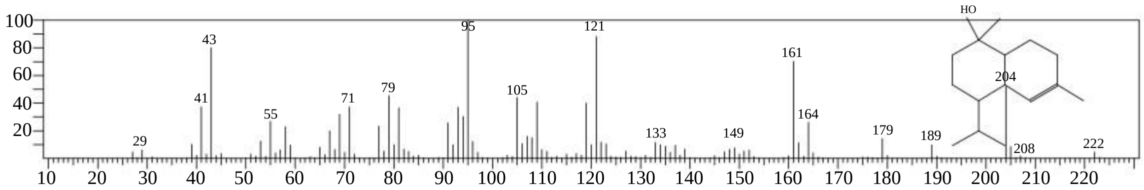

HO
100
80
60
40
20
29
41
43
55
71
79
95
105
121
133
149
161
164
179
189
204
208
222
10 20 30 40 50 60 70 80 90 100 110 120 130 140 150 160 170 180 190 200 210 220

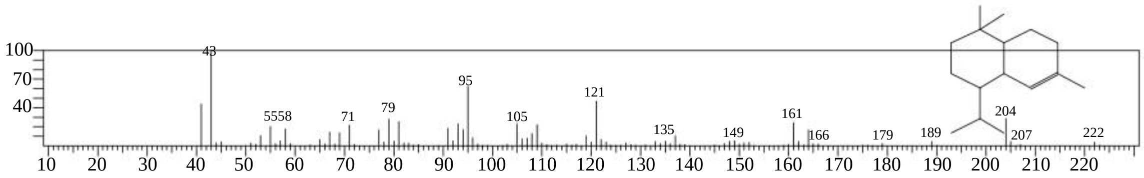

100
70
40
43
5558
71
79
95
105
121
135
149
161
166
179
189
204
207
222
10 20 30 40 50 60 70 80 90 100 110 120 130 140 150 160 170 180 190 200 210 220

OL

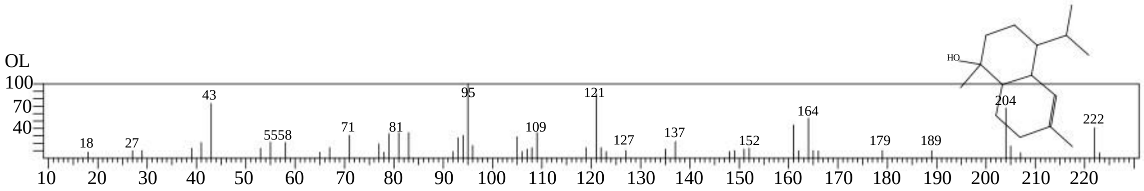

HO
100
70
40
18
27
43
5558
71
81
95
109
121
127
137
152
164
179
189
204
222
10 20 30 40 50 60 70 80 90 100 110 120 130 140 150 160 170 180 190 200 210 220

OL

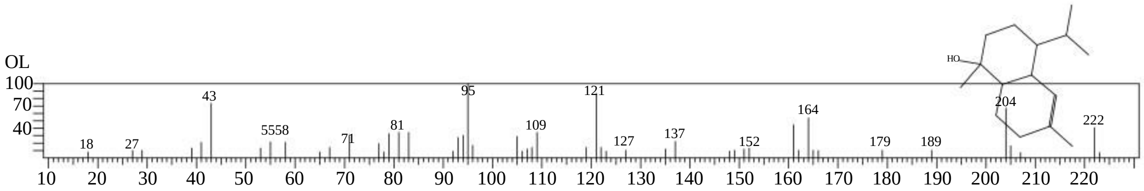

HO
100
70
40
18
27
43
5558
71
81
95
109
121
127
137
152
164
179
189
204
222
10 20 30 40 50 60 70 80 90 100 110 120 130 140 150 160 170 180 190 200 210 220

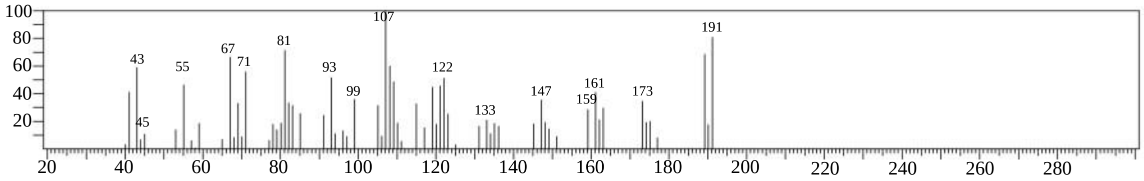
100
80
60
40
20
43
45
55
67
71
81
93
99
107
122
133
147
159
161
173
191
20
40
60
80
100
120
140
160
180
200
220
240
260
280

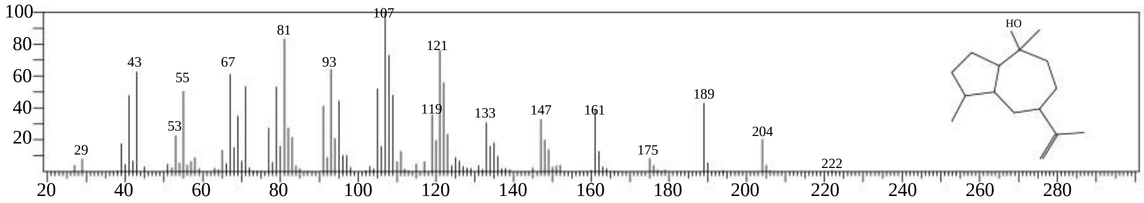
100
80
60
40
20
29
43
53
55
67
81
93
107
119
121
133
147
161
175
189
204
222
HO
20
40
60
80
100
120
140
160
180
200
220
240
260
280

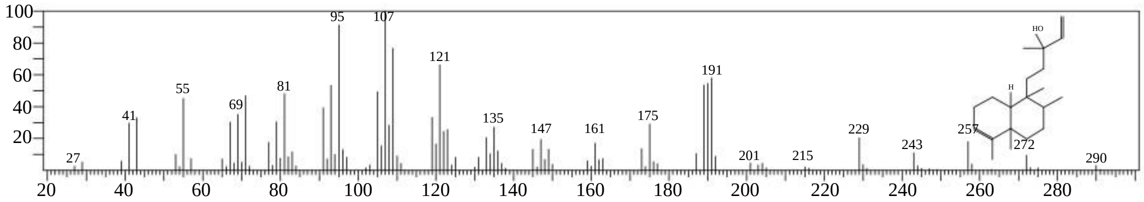
100
80
60
40
20
27
41
55
69
81
95
107
121
135
147
161
175
191
201
215
229
243
257
272
290
HO
H
20
40
60
80
100
120
140
160
180
200
220
240
260
280

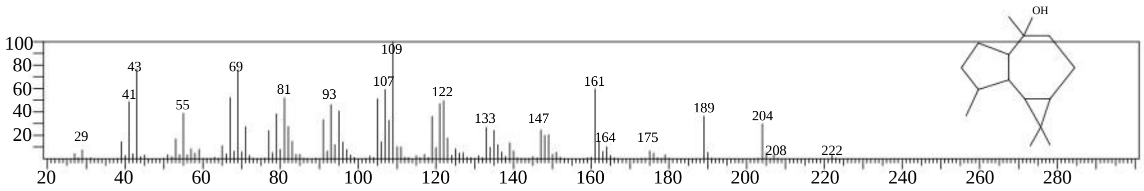
OH
100
80
60
40
20
29
41
43
55
69
81
93
107
109
122
133
147
161
164
175
189
204
208
222
20
40
60
80
100
120
140
160
180
200
220
240
260
280

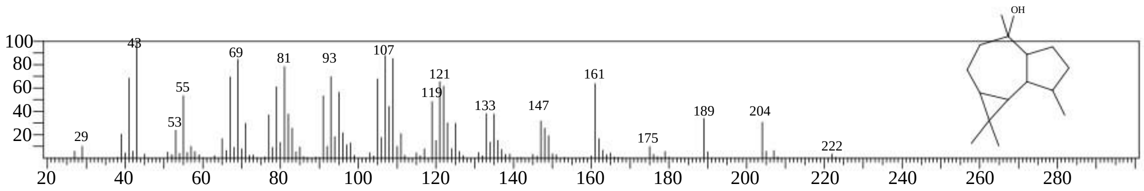
OH
100
80
60
40
20
29
43
53
55
69
81
93
107
119
121
133
147
161
175
189
204
222
20
40
60
80
100
120
140
160
180
200
220
240
260
280

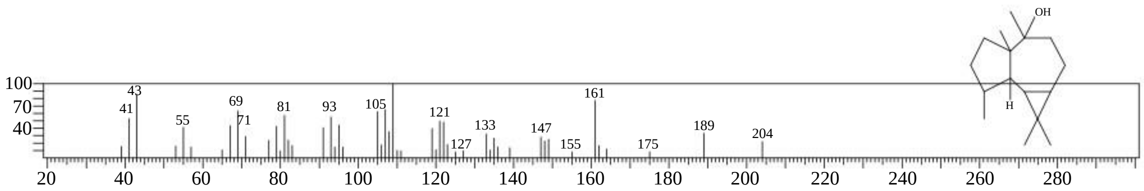
OH
H
100
70
40
41
43
55
69
71
81
93
105
121
127
133
147
155
161
175
189
204
20
40
60
80
100
120
140
160
180
200
220
240
260
280

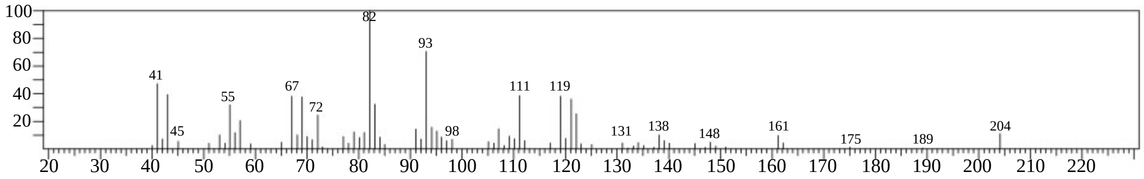
100
80
60
40
20
41
45
55
67
72
82
93
98
111
119
131
138
148
161
175
189
204
20 30 40 50 60 70 80 90 100 110 120 130 140 150 160 170 180 190 200 210 220

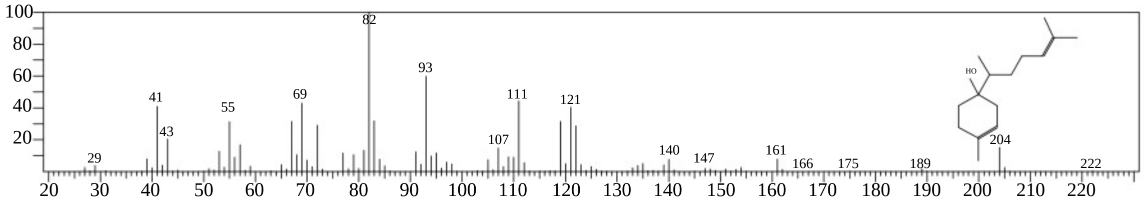
100
80
60
40
20
29
41
43
55
69
82
93
107
111
121
140
147
161
166
175
189
204
222
HO
20 30 40 50 60 70 80 90 100 110 120 130 140 150 160 170 180 190 200 210 220

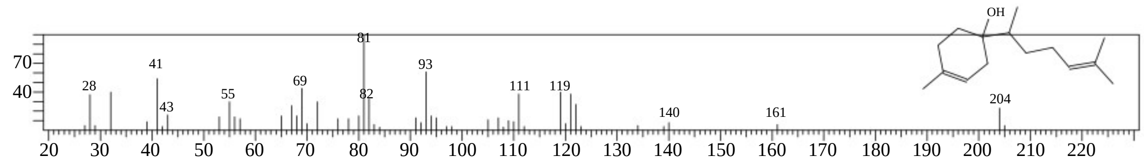
OH
70
40
28
41
43
55
69
81
82
93
111
119
140
161
204
20 30 40 50 60 70 80 90 100 110 120 130 140 150 160 170 180 190 200 210 220

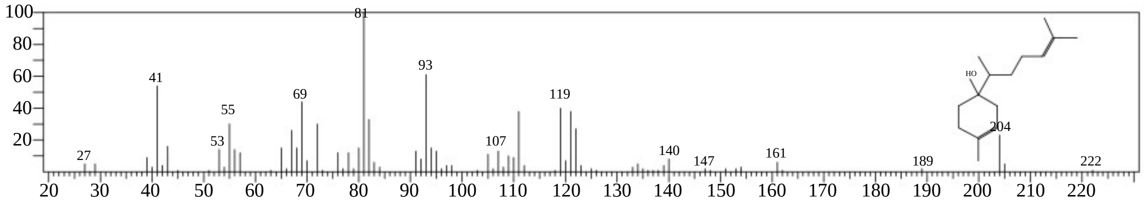
100
80
60
40
20
27
41
53
55
69
81
93
107
119
140
147
161
189
204
222
HO
20 30 40 50 60 70 80 90 100 110 120 130 140 150 160 170 180 190 200 210 220

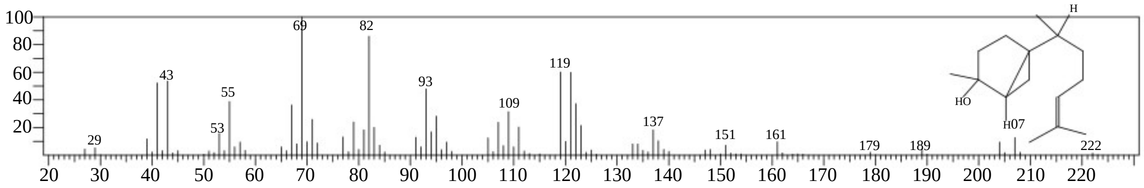
H
100
80
60
40
20
29
43
53
55
69
82
93
109
119
137
151
161
179
189
H07
222
HO
20 30 40 50 60 70 80 90 100 110 120 130 140 150 160 170 180 190 200 210 220

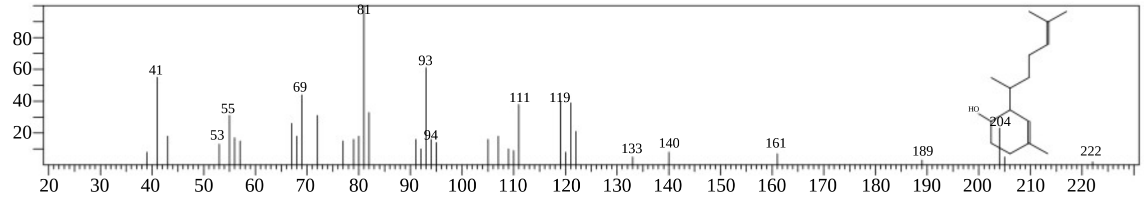
80
60
40
20
41
53
55
69
81
93
94
111
119
133
140
161
189
204
222
HO
20 30 40 50 60 70 80 90 100 110 120 130 140 150 160 170 180 190 200 210 220

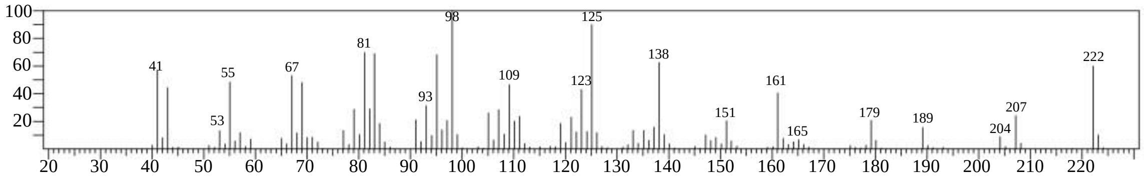
100
80
60
40
20
41
53
55
67
81
93
98
109
123
125
138
151
161
165
179
189
204
207
222
20 30 40 50 60 70 80 90 100 110 120 130 140 150 160 170 180 190 200 210 220

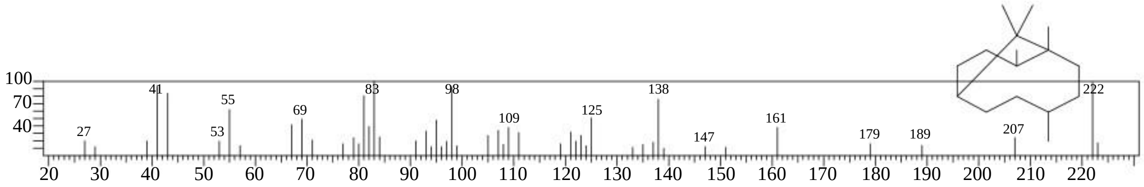
100
70
40
27
41
53
55
69
83
98
109
125
138
147
161
179
189
207
222
20 30 40 50 60 70 80 90 100 110 120 130 140 150 160 170 180 190 200 210 220

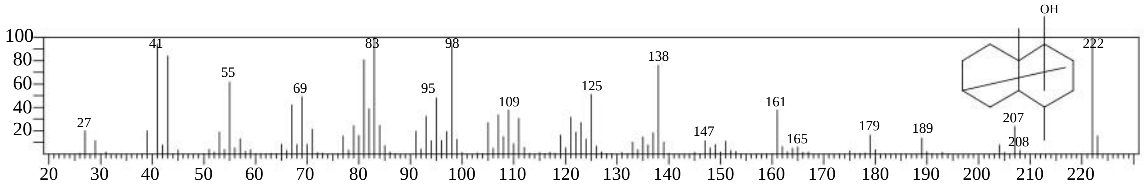
OH
100
80
60
40
20
27
41
55
69
83
95
98
109
125
138
147
161
165
179
189
207
208
222
20 30 40 50 60 70 80 90 100 110 120 130 140 150 160 170 180 190 200 210 220

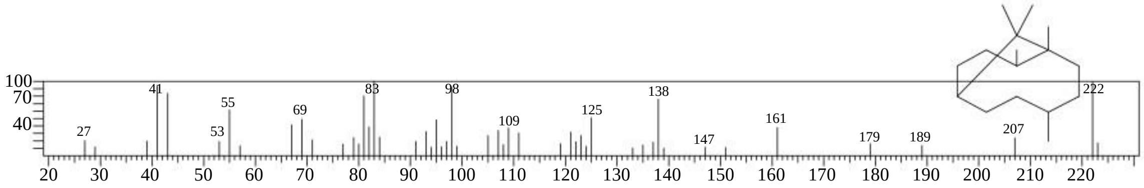
100
70
40
27
41
53
55
69
83
98
109
125
138
147
161
179
189
207
222
20 30 40 50 60 70 80 90 100 110 120 130 140 150 160 170 180 190 200 210 220

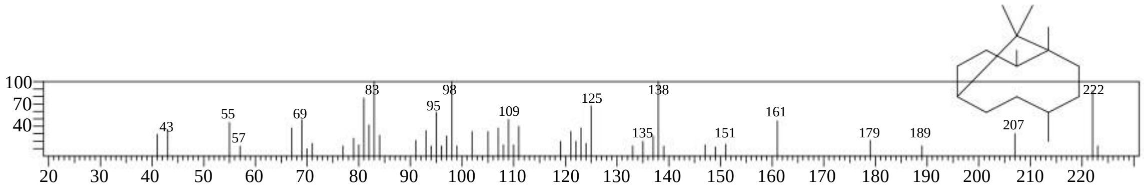
100
70
40
43
55
57
69
83
95
98
109
125
135
138
151
161
179
189
207
222
20 30 40 50 60 70 80 90 100 110 120 130 140 150 160 170 180 190 200 210 220

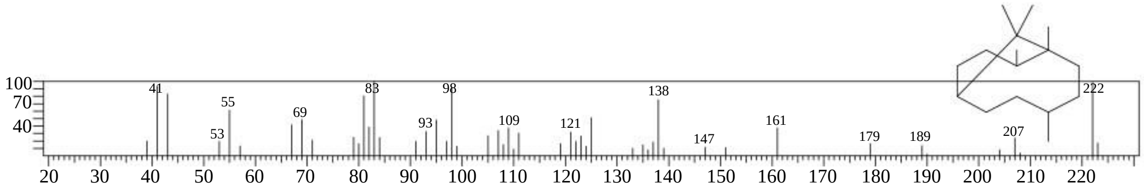
100
70
40
41
53
55
69
83
93
98
109
121
138
147
161
179
189
207
222
20 30 40 50 60 70 80 90 100 110 120 130 140 150 160 170 180 190 200 210 220

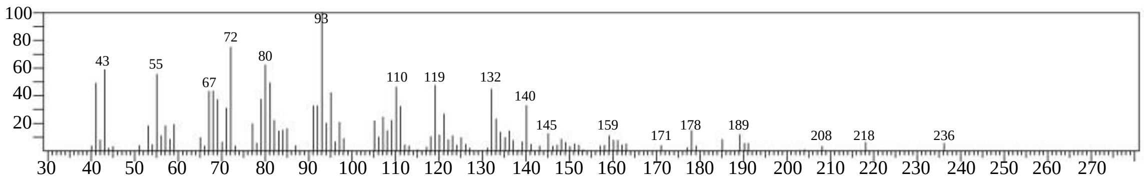

100 80 60 40 20
43 55 67 72 80 93 110 119 132 140 145 159 171 178 189 208 218 236
30 40 50 60 70 80 90 100 110 120 130 140 150 160 170 180 190 200 210 220 230 240 250 260 270

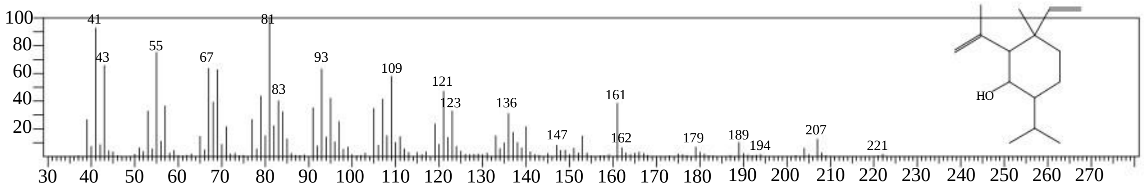

100 80 60 40 20
41 43 55 67 81 83 93 109 121 123 136 147 161 162 179 189 194 207 221
HO
30 40 50 60 70 80 90 100 110 120 130 140 150 160 170 180 190 200 210 220 230 240 250 260 270

:

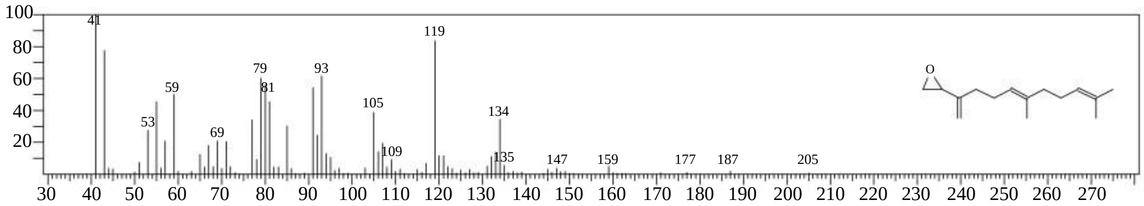

100 80 60 40 20
41 53 59 69 79 81 93 105 109 119 134 135 147 159 177 187 205
O
30 40 50 60 70 80 90 100 110 120 130 140 150 160 170 180 190 200 210 220 230 240 250 260 270

NE-3-OL $$ 3,7,11-T

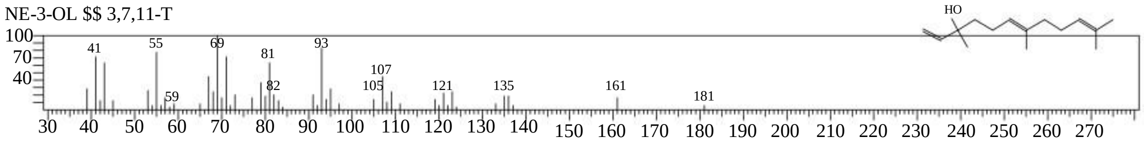

HO
100 70 40
41 55 59 69 81 82 93 105 107 121 135 161 181
30 40 50 60 70 80 90 100 110 120 130 140 150 160 170 180 190 200 210 220 230 240 250 260 270

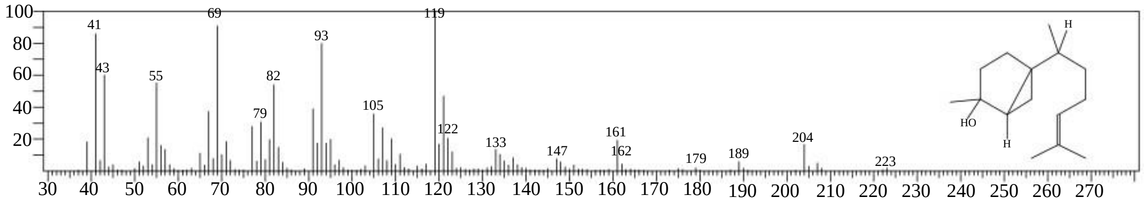

100 80 60 40 20
41 43 55 69 79 82 93 105 119 122 133 147 161 162 179 189 204 223
H HO H
30 40 50 60 70 80 90 100 110 120 130 140 150 160 170 180 190 200 210 220 230 240 250 260 270

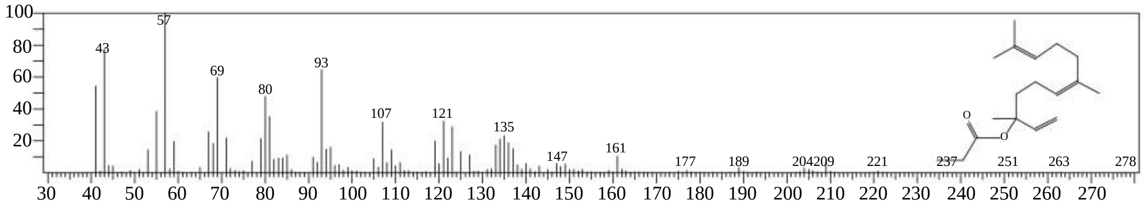

100 80 60 40 20
43 57 69 80 93 107 121 135 147 161 177 189 204209 221 237 251 263 278
O O
30 40 50 60 70 80 90 100 110 120 130 140 150 160 170 180 190 200 210 220 230 240 250 260 270

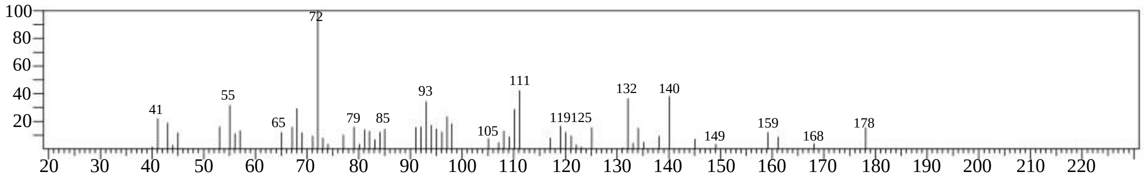
41 55 65 72 79 85 93 105 111 119 125 132 140 149 159 168 178

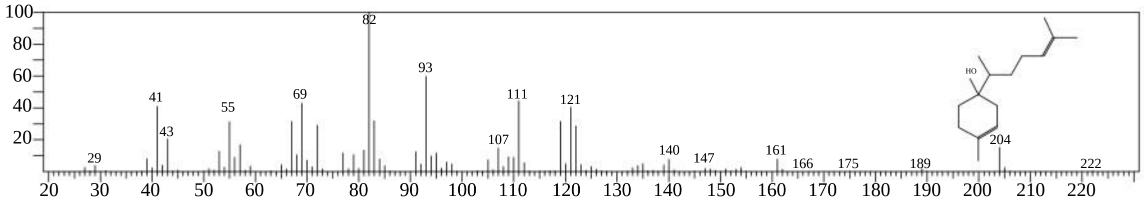
29 41 43 55 69 82 93 107 111 121 140 147 161 166 175 189 204 222
HO

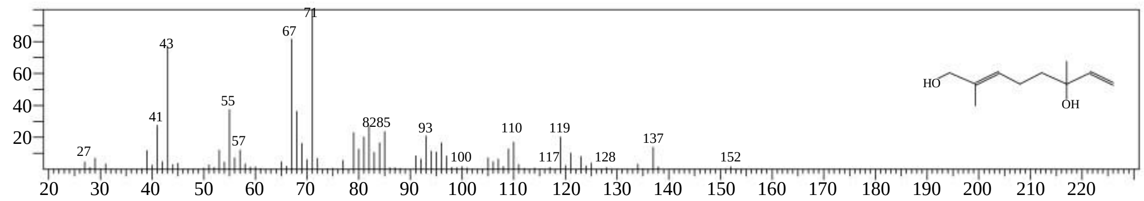
27 41 43 55 57 67 71 82 85 93 100 110 117 119 128 137 152
HO
OH

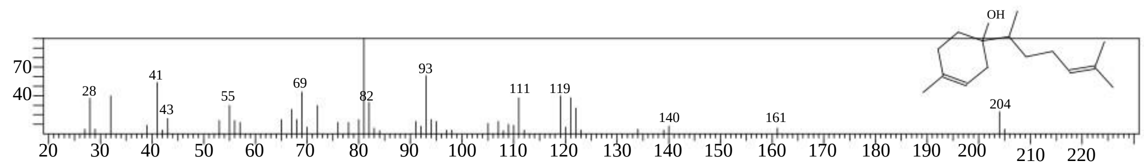
28 41 43 55 69 82 93 111 119 140 161 204
OH

:

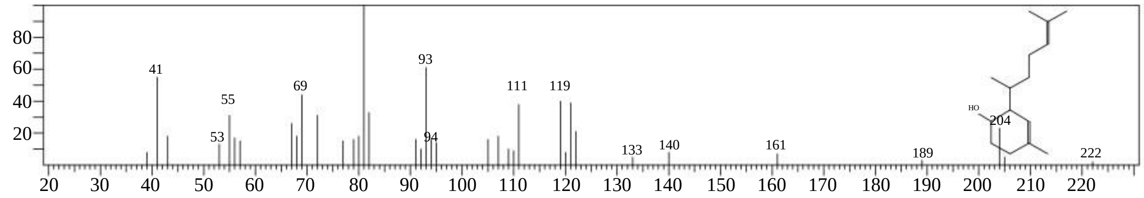
41 53 55 69 93 94 111 119 133 140 161 189 204 222
HO

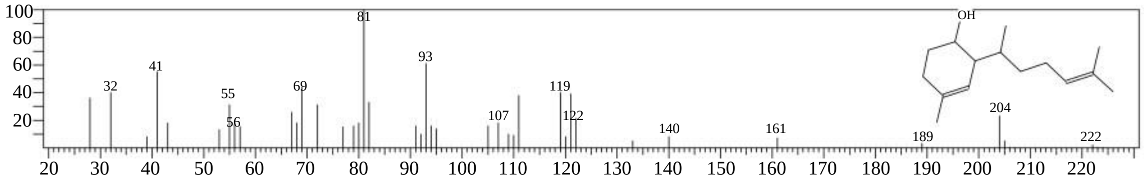
32 41 55 56 69 81 93 107 119 122 140 161 189 204 222
OH

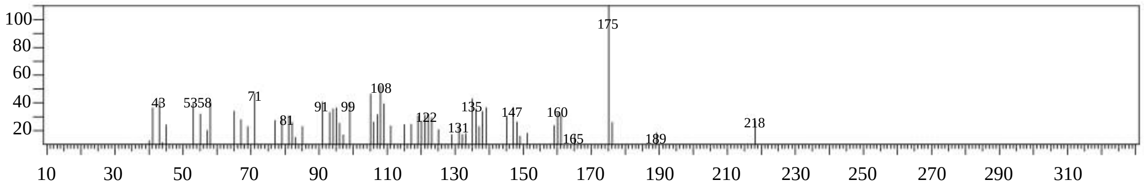
100
80
60
40
20
175
43
5358
71
81
91
99
108
122
131
135
147
160
165
189
218
10 30 50 70 90 110 130 150 170 190 210 230 250 270 290 310

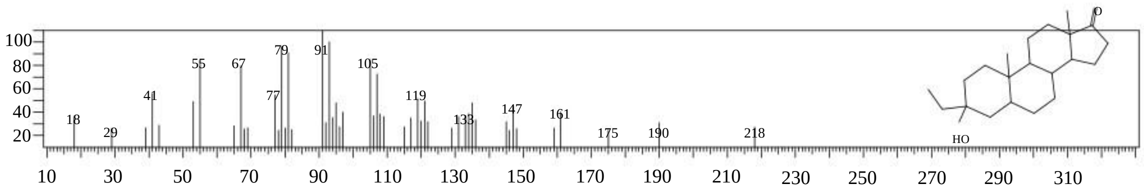
100
80
60
40
20
18
29
41
55
67
77
79
91
105
119
133
147
161
175
190
218
O
HO
10 30 50 70 90 110 130 150 170 190 210 230 250 270 290 310

:

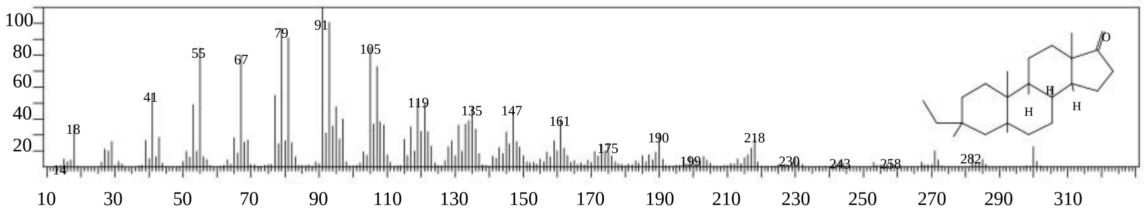
100
80
60
40
20
14
18
41
55
67
79
91
105
119
135
147
161
175
190
199
218
230
243
258
282
O
H
H
H
10 30 50 70 90 110 130 150 170 190 210 230 250 270 290 310

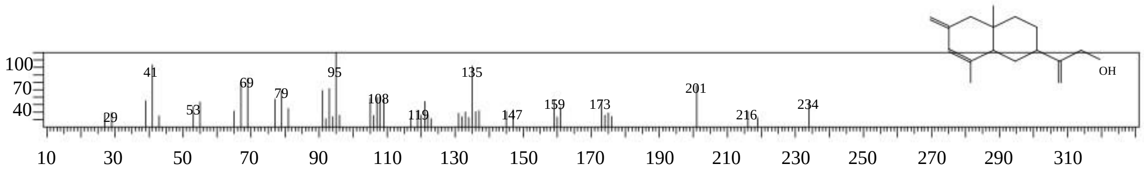
100
70
40
29
41
53
69
79
95
108
119
135
147
159
173
201
216
234
OH
10 30 50 70 90 110 130 150 170 190 210 230 250 270 290 310

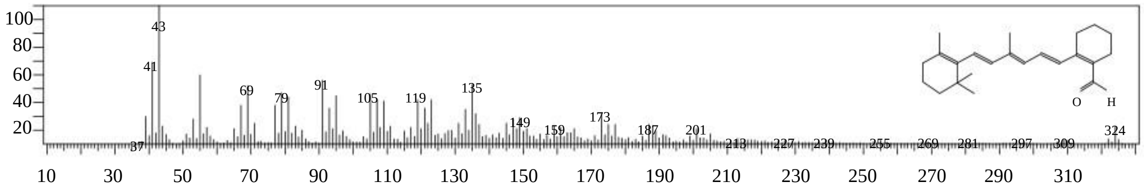
100
80
60
40
20
37
41
43
69
79
91
105
119
135
149
159
173
187
201
213
227
239
255
269
281
297
309
324
O
H
10 30 50 70 90 110 130 150 170 190 210 230 250 270 290 310

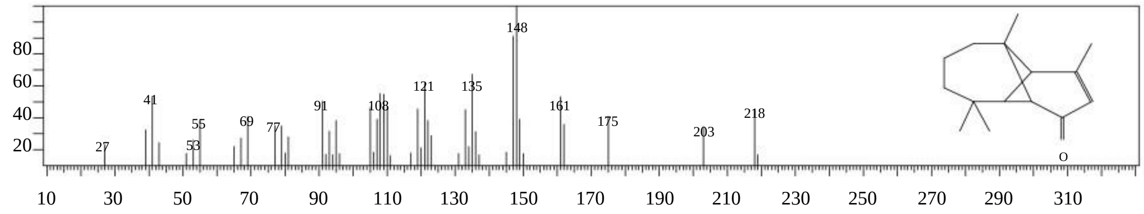
80
60
40
20
27
41
53
55
69
77
91
108
121
135
148
161
175
203
218
O
10 30 50 70 90 110 130 150 170 190 210 230 250 270 290 310

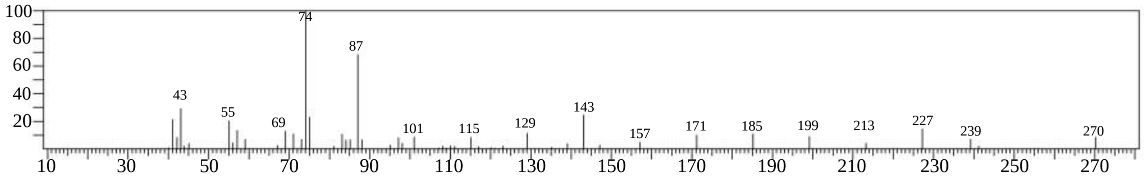

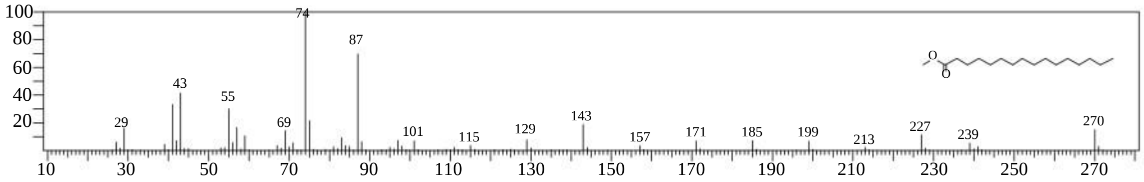

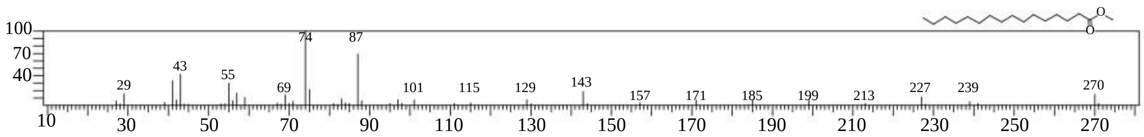

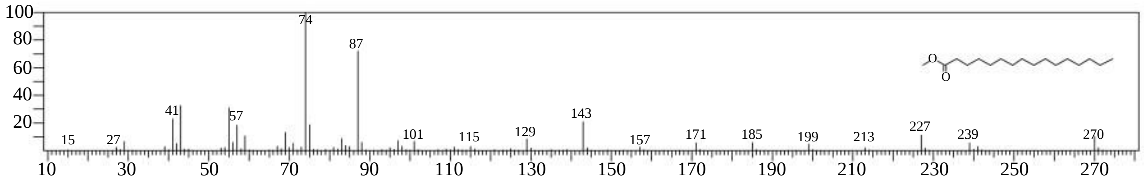

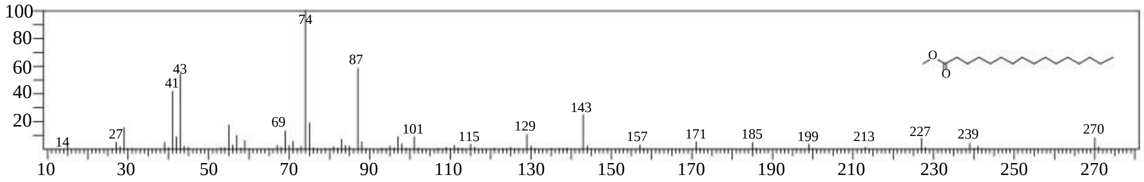

0 $$ UNIPHAT A60

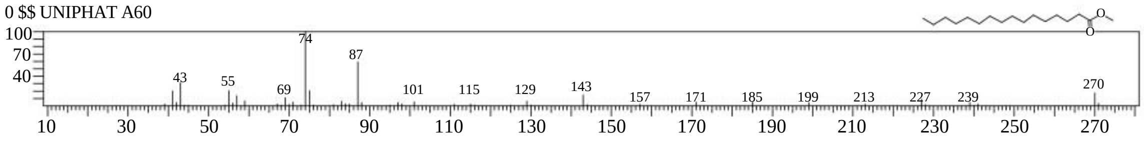

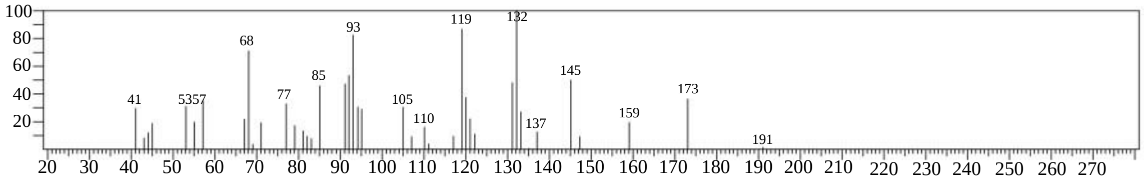
100
80
60
40
20
41
5357
68
77
85
93
105
110
119
132
137
145
159
173
191
20 30 40 50 60 70 80 90 100 110 120 130 140 150 160 170 180 190 200 210 220 230 240 250 260 270

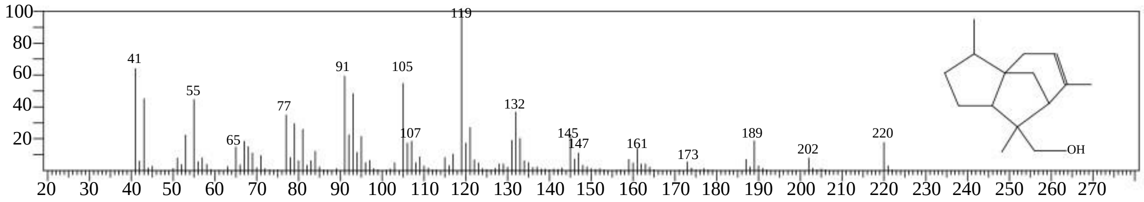
100
80
60
40
20
41
55
65
77
91
105
107
119
132
145
147
161
173
189
202
220
OH
20 30 40 50 60 70 80 90 100 110 120 130 140 150 160 170 180 190 200 210 220 230 240 250 260 270

:

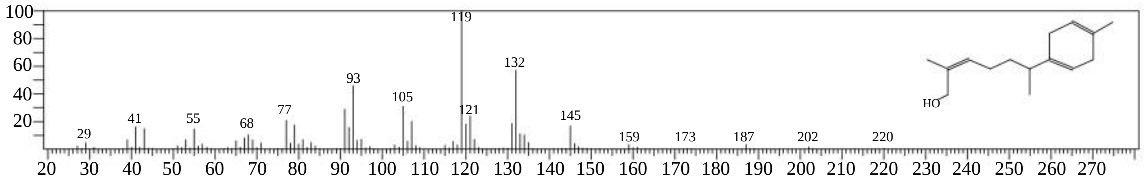
100
80
60
40
20
29
41
55
68
77
93
105
119
121
132
145
159
173
187
202
220
HO
20 30 40 50 60 70 80 90 100 110 120 130 140 150 160 170 180 190 200 210 220 230 240 250 260 270

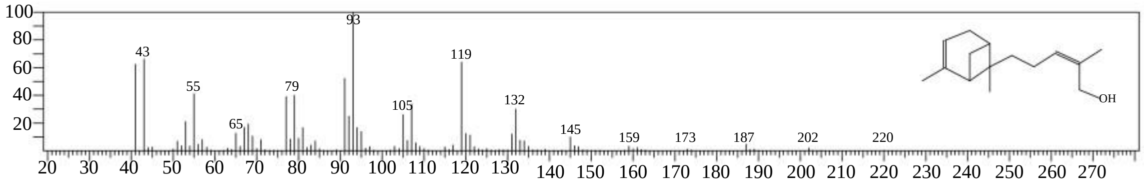
100
80
60
40
20
43
55
65
79
93
105
119
132
145
159
173
187
202
220
OH
20 30 40 50 60 70 80 90 100 110 120 130 140 150 160 170 180 190 200 210 220 230 240 250 260 270

:

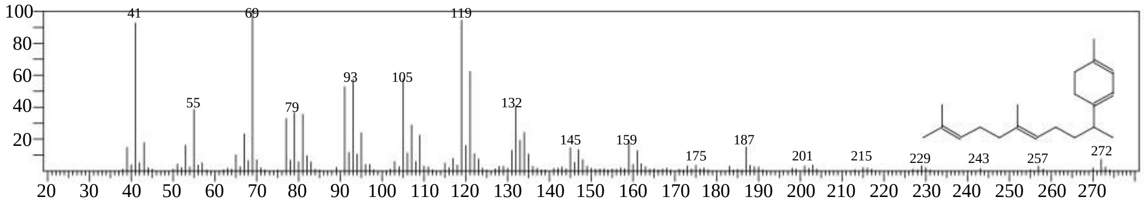
100
80
60
40
20
41
55
69
79
93
105
119
132
145
159
175
187
201
215
229
243
257
272
20 30 40 50 60 70 80 90 100 110 120 130 140 150 160 170 180 190 200 210 220 230 240 250 260 270

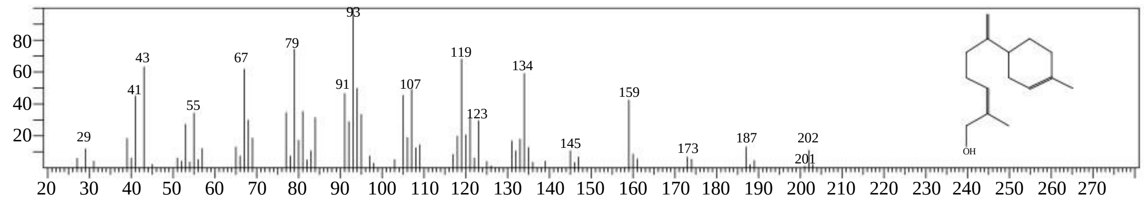
80
60
40
20
29
41
43
55
67
79
91
93
107
119
123
134
145
159
173
187
202
201
OH
20 30 40 50 60 70 80 90 100 110 120 130 140 150 160 170 180 190 200 210 220 230 240 250 260 270

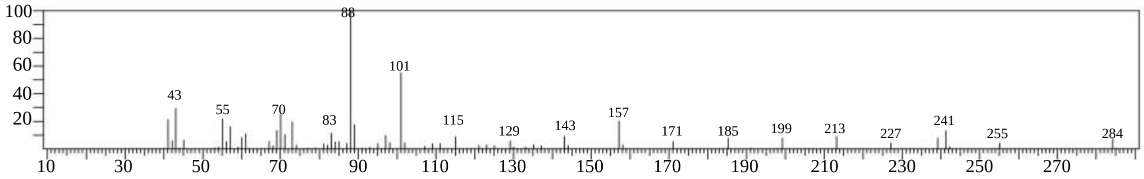
100
80
60
40
20
88
101
43
55
70
83
115
129
143
157
171
185
199
213
227
241
255
284
10 30 50 70 90 110 130 150 170 190 210 230 250 270

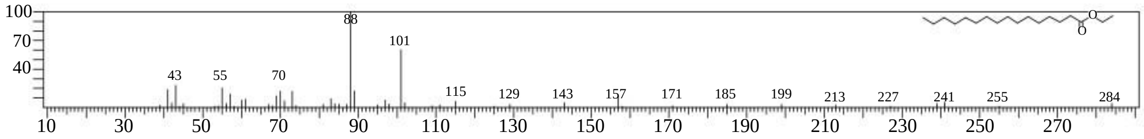
100
70
40
88
101
43
55
70
115
129
143
157
171
185
199
213
227
241
255
284
O
O
10 30 50 70 90 110 130 150 170 190 210 230 250 270

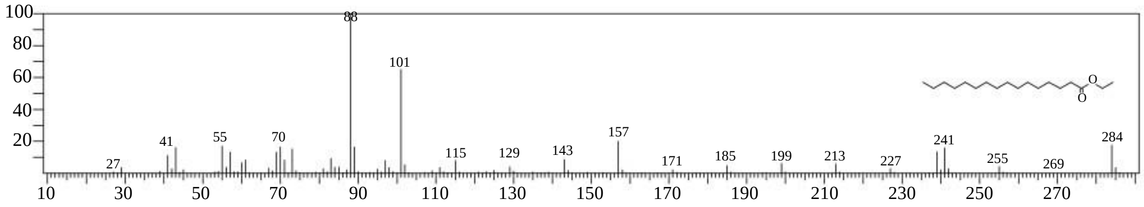
100
80
60
40
20
88
101
27
41
55
70
115
129
143
157
171
185
199
213
227
241
255
269
284
O
O
10 30 50 70 90 110 130 150 170 190 210 230 250 270

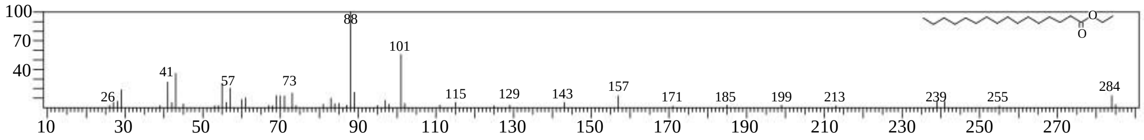
100
70
40
88
101
26
41
57
73
115
129
143
157
171
185
199
213
239
255
284
O
O
10 30 50 70 90 110 130 150 170 190 210 230 250 270

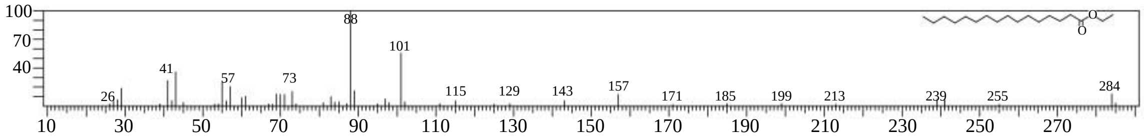
100
70
40
88
101
26
41
57
73
115
129
143
157
171
185
199
213
239
255
284
O
O
10 30 50 70 90 110 130 150 170 190 210 230 250 270

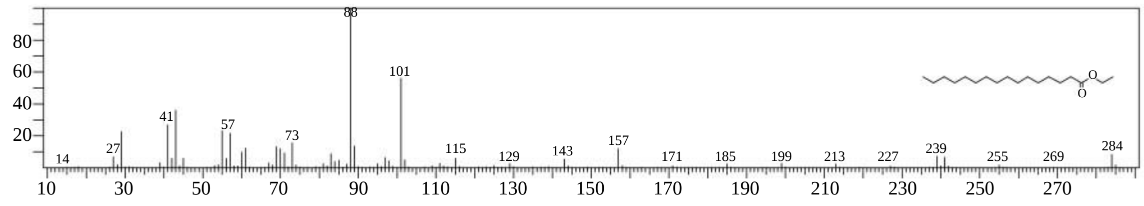
80
60
40
20
88
101
14
27
41
57
73
115
129
143
157
171
185
199
213
227
239
255
269
284
O
O
10 30 50 70 90 110 130 150 170 190 210 230 250 270

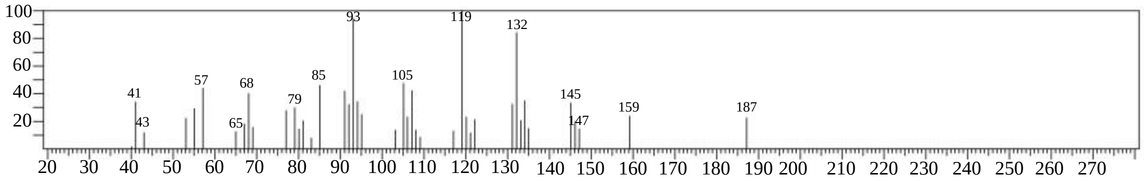
41
43
57
65
68
79
85
93
105
119
132
145
147
159
187

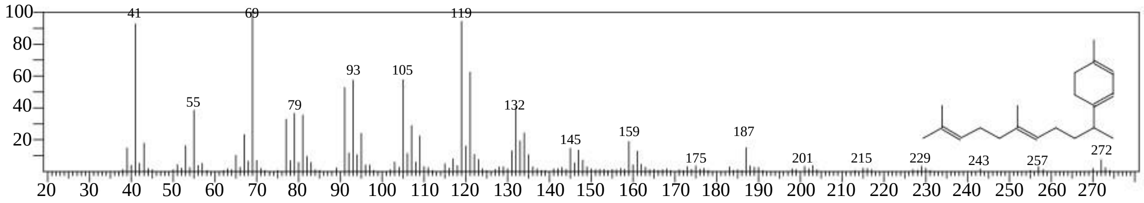
41
55
69
79
93
105
119
132
145
159
175
187
201
215
229
243
257
272

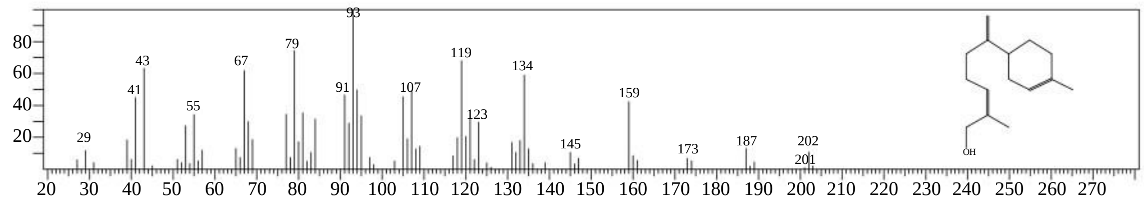
29
41
43
55
67
79
91
93
107
119
123
134
145
159
173
187
202
201
OH

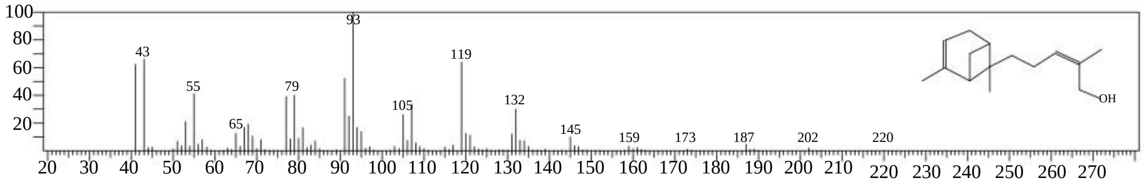
43
55
65
79
93
105
119
132
145
159
173
187
202
220
OH

:

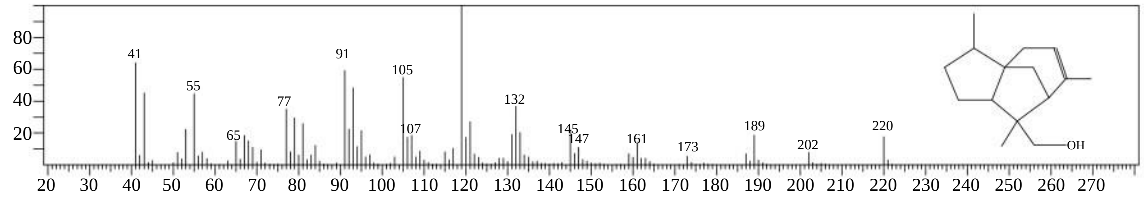
41
55
65
77
91
105
107
132
145
147
161
173
189
202
220
OH

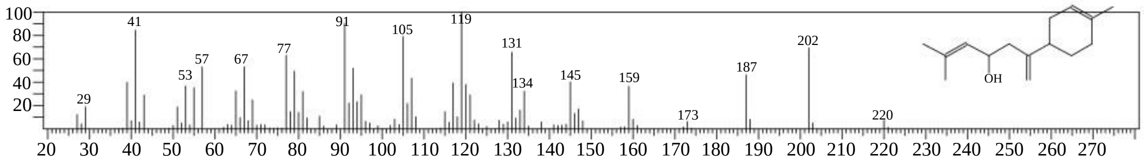
29
41
53
57
67
77
91
105
119
131
134
145
159
173
187
202
220
OH

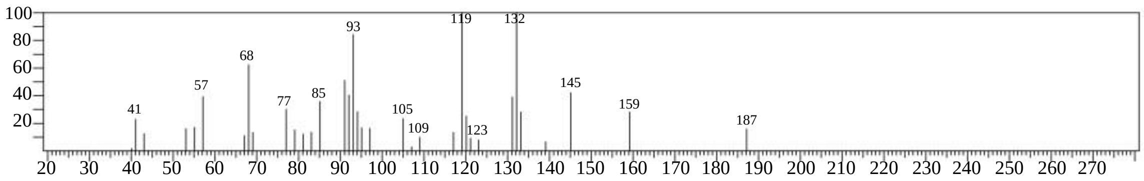
41
57
68
77
85
93
105
109
119
123
132
145
159
187

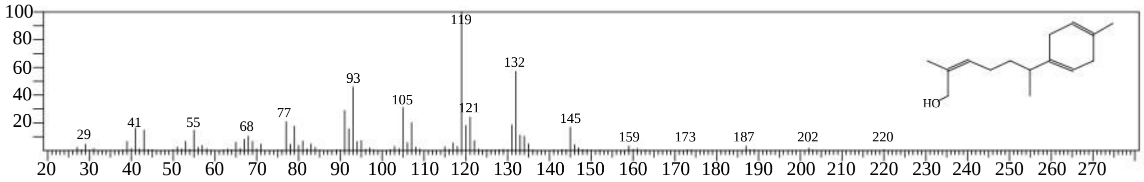
29
41
55
68
77
93
105
119
121
132
145
159
173
187
202
220
HO

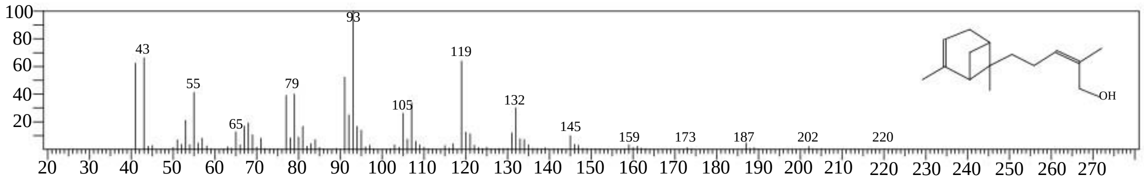
43
55
65
79
93
105
119
132
145
159
173
187
202
220
OH

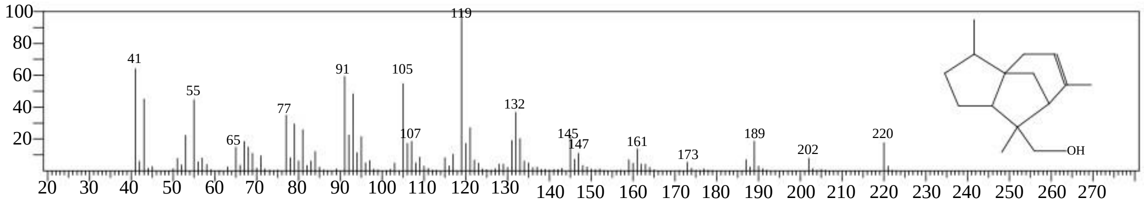
41
55
65
77
91
105
107
119
132
145
147
161
173
189
202
220
OH

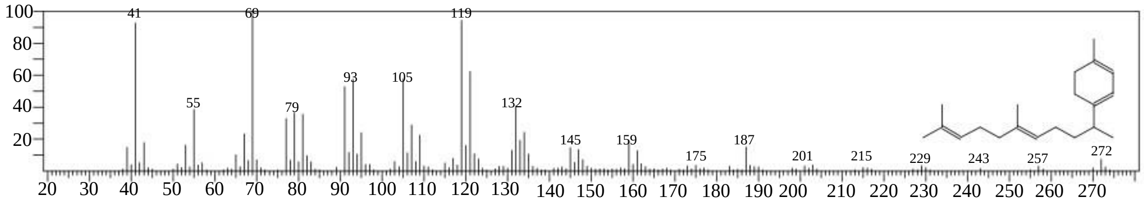
41
55
69
79
93
105
119
132
145
159
175
187
201
215
229
243
257
272

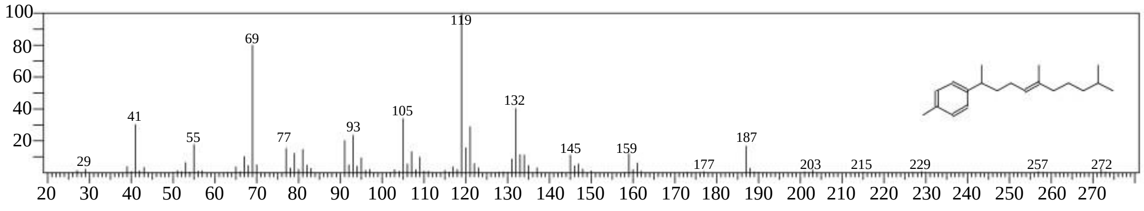
29
41
55
69
77
93
105
119
132
145
159
177
187
203
215
229
257
272

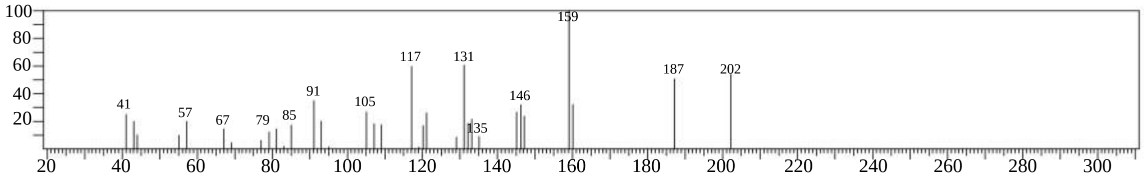
41 57 67 79 85 91 105 117 131 135 146 159 187 202

-ol ester

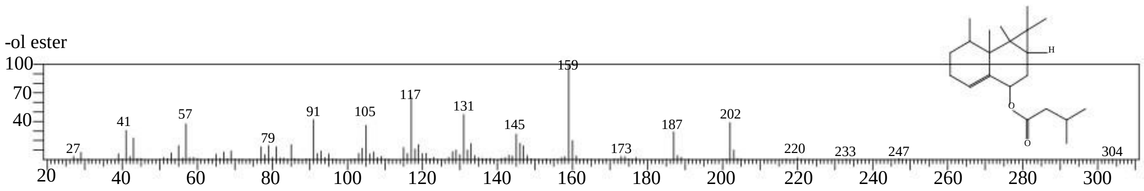
27 41 57 79 91 105 117 131 145 159 173 187 202 220 233 247 304
H
O
O

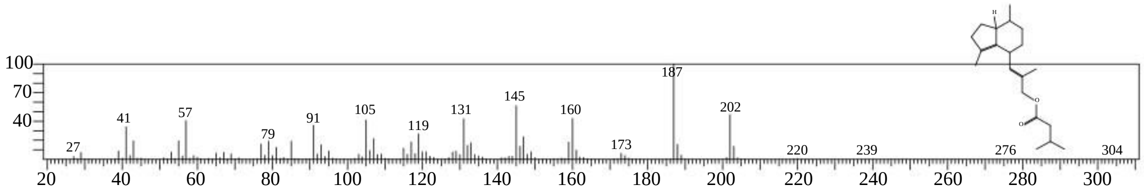
27 41 57 79 91 105 119 131 145 160 173 187 202 220 239 276 304
H

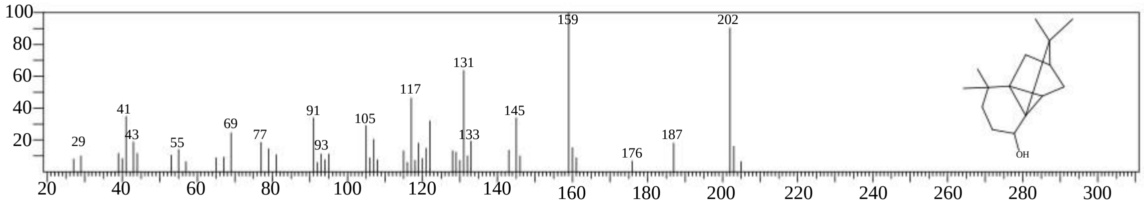
29 41 43 55 69 77 91 93 105 117 131 133 145 159 176 187 202
OH

:

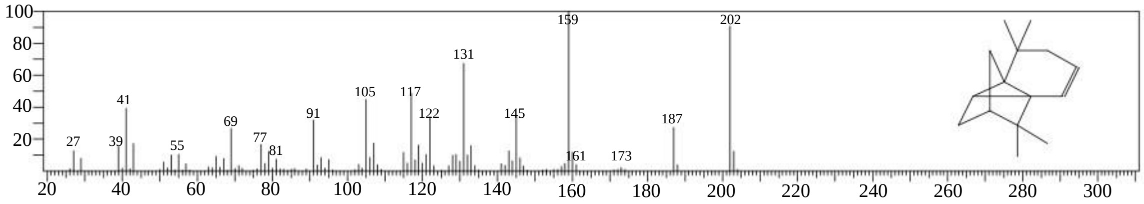
27 39 41 55 69 77 81 91 105 117 122 131 145 159 161 173 187 202

:

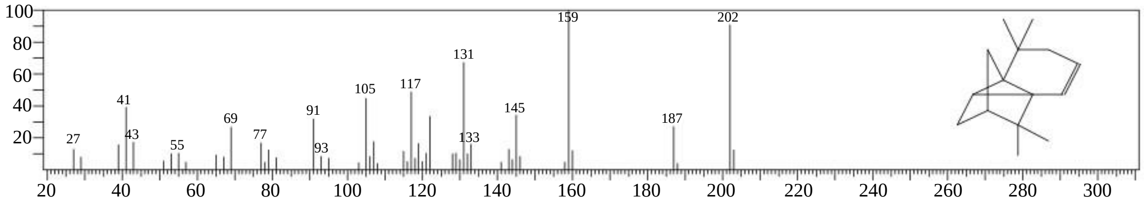
27 41 43 55 69 77 91 93 105 117 131 133 145 159 187 202

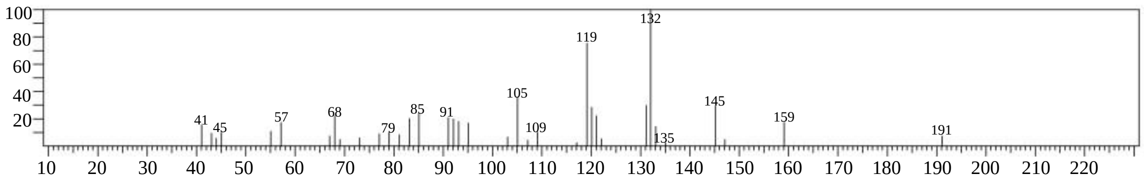
100
80
60
40
20
41 45 57 68 79 85 91 105 109 119 132 135 145 159 191
10 20 30 40 50 60 70 80 90 100 110 120 130 140 150 160 170 180 190 200 210 220

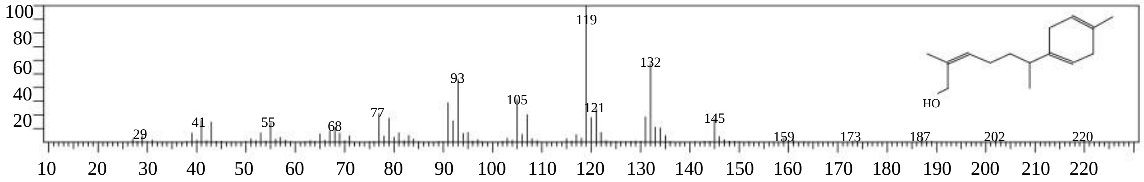
100
80
60
40
20
29 41 55 68 77 93 105 119 121 132 145 159 173 187 202 220
HO
10 20 30 40 50 60 70 80 90 100 110 120 130 140 150 160 170 180 190 200 210 220

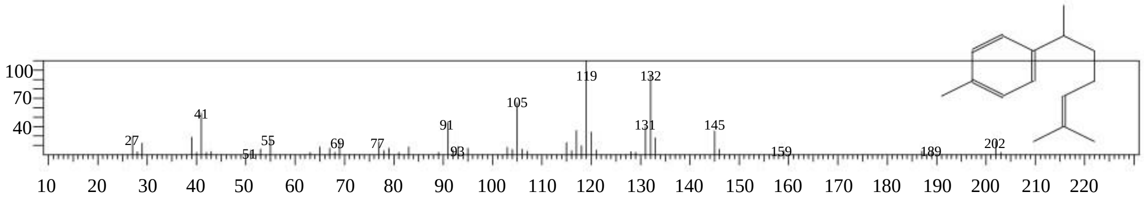
100
70
40
27 41 51 55 69 77 91 93 105 119 131 132 145 159 189 202
10 20 30 40 50 60 70 80 90 100 110 120 130 140 150 160 170 180 190 200 210 220

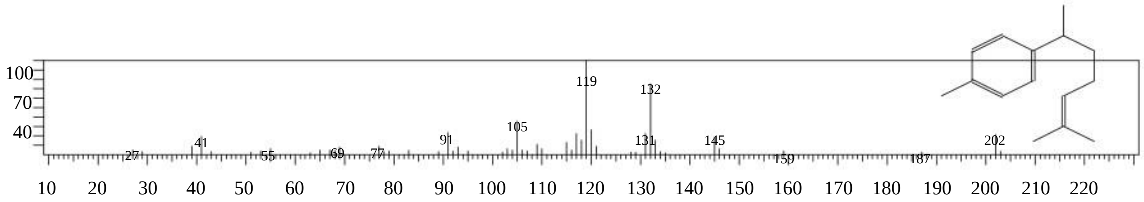
100
70
40
27 41 55 69 77 91 105 119 131 132 145 159 187 202
10 20 30 40 50 60 70 80 90 100 110 120 130 140 150 160 170 180 190 200 210 220

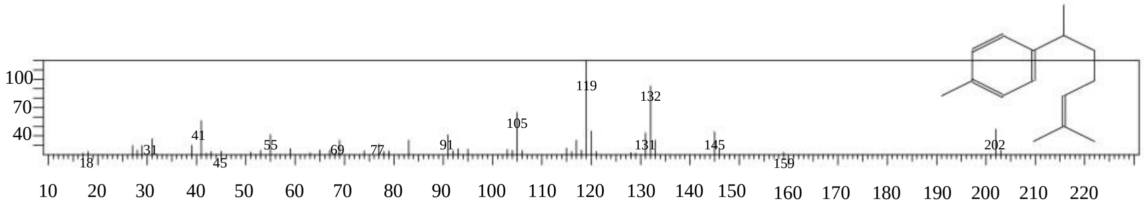
100
70
40
18 31 41 45 55 69 77 91 105 119 131 132 145 159 202
10 20 30 40 50 60 70 80 90 100 110 120 130 140 150 160 170 180 190 200 210 220

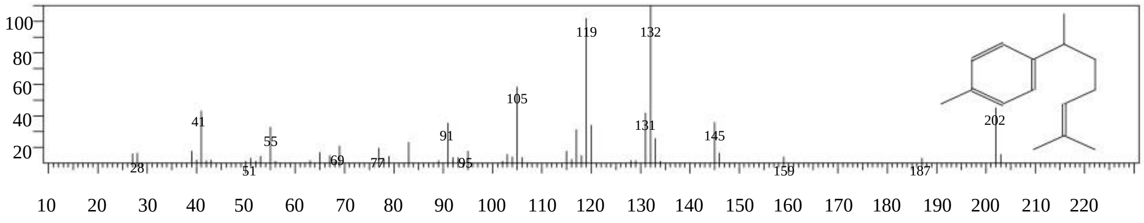
100
80
60
40
20
28 41 51 55 69 77 91 95 105 119 131 132 145 159 187 202
10 20 30 40 50 60 70 80 90 100 110 120 130 140 150 160 170 180 190 200 210 220

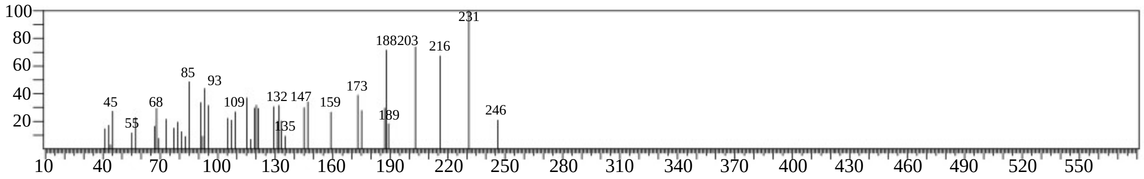

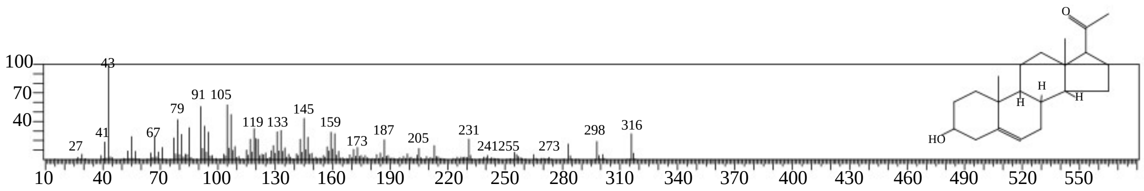

:2 Entry:148070 Library:NIST17M1.lib

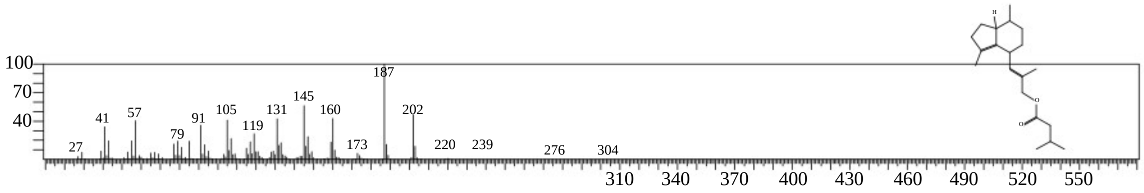

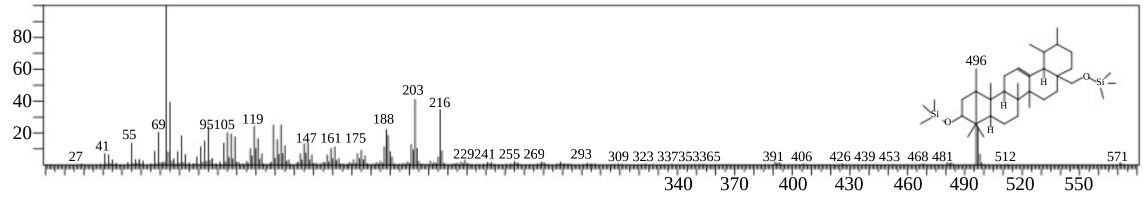

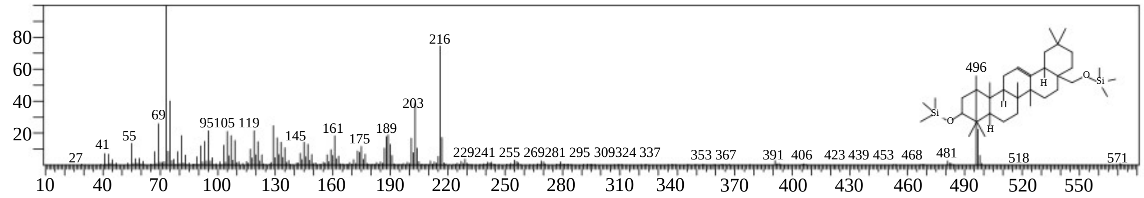

:

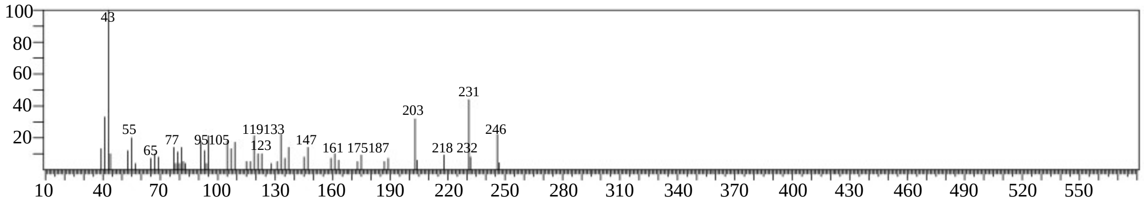

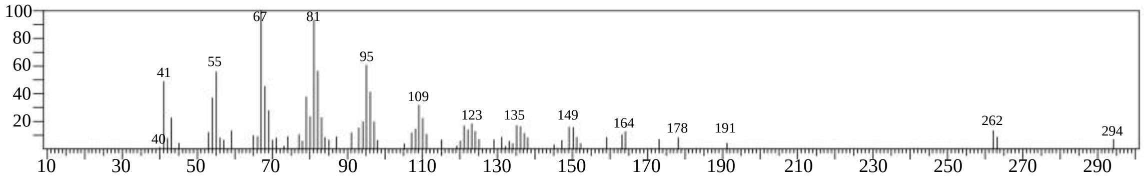

100
80
60
40
20
41 40 55 67 81 95 109 123 135 149 164 178 191 262 294
10 30 50 70 90 110 130 150 170 190 210 230 250 270 290

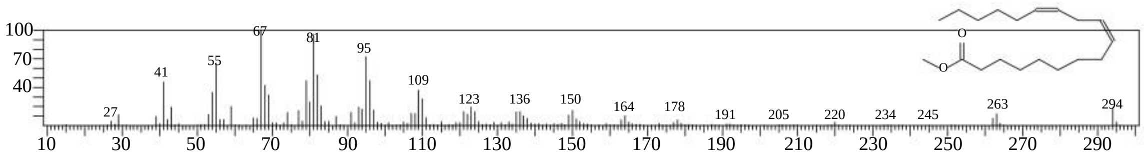

O
O
100
70
40
27 41 55 67 81 95 109 123 136 150 164 178 191 205 220 234 245 263 294
10 30 50 70 90 110 130 150 170 190 210 230 250 270 290

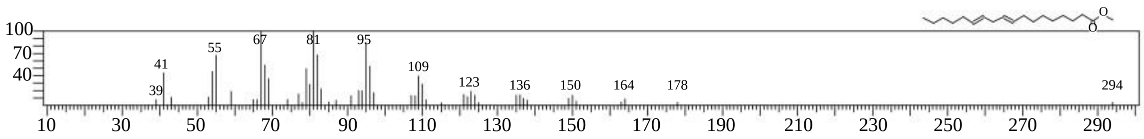

O
O
100
70
40
39 41 55 67 81 95 109 123 136 150 164 178 294
10 30 50 70 90 110 130 150 170 190 210 230 250 270 290

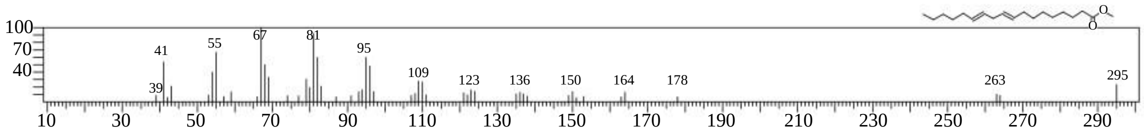

O
O
100
70
40
39 41 55 67 81 95 109 123 136 150 164 178 263 295
10 30 50 70 90 110 130 150 170 190 210 230 250 270 290

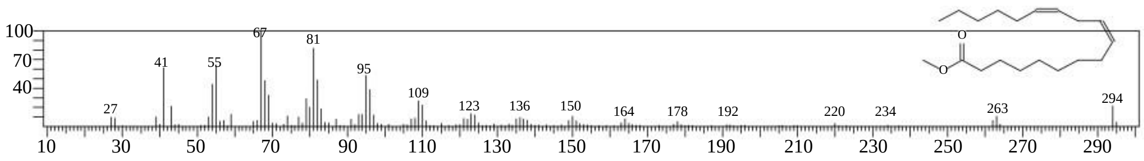

O
O
100
70
40
27 41 55 67 81 95 109 123 136 150 164 178 192 220 234 263 294
10 30 50 70 90 110 130 150 170 190 210 230 250 270 290

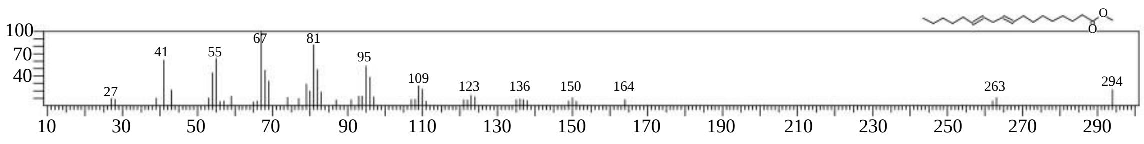

O
O
100
70
40
27 41 55 67 81 95 109 123 136 150 164 263 294
10 30 50 70 90 110 130 150 170 190 210 230 250 270 290

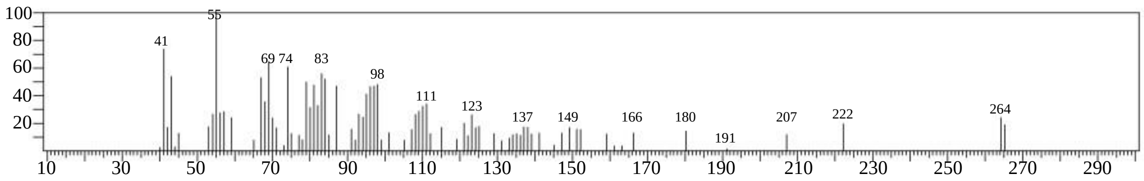
100
80
60
40
20
41 55 69 74 83 98 111 123 137 149 166 180 191 207 222 264
10 30 50 70 90 110 130 150 170 190 210 230 250 270 290

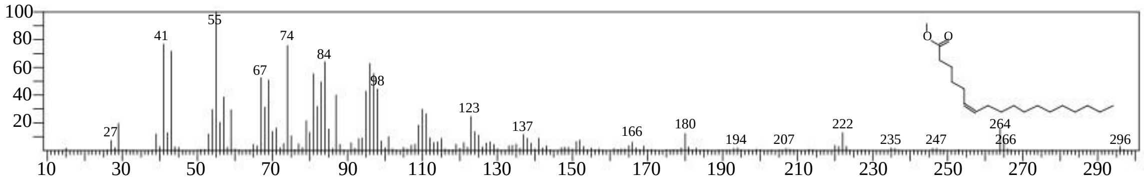
100
80
60
40
20
27 41 55 67 74 84 98 123 137 166 180 194 207 222 235 247 264 266 296
10 30 50 70 90 110 130 150 170 190 210 230 250 270 290

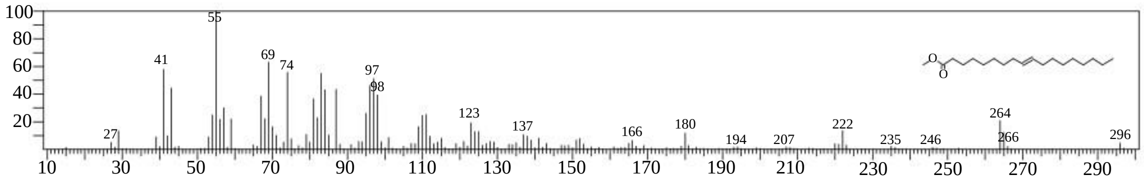
100
80
60
40
20
27 41 55 69 74 97 98 123 137 166 180 194 207 222 235 246 264 266 296
10 30 50 70 90 110 130 150 170 190 210 230 250 270 290

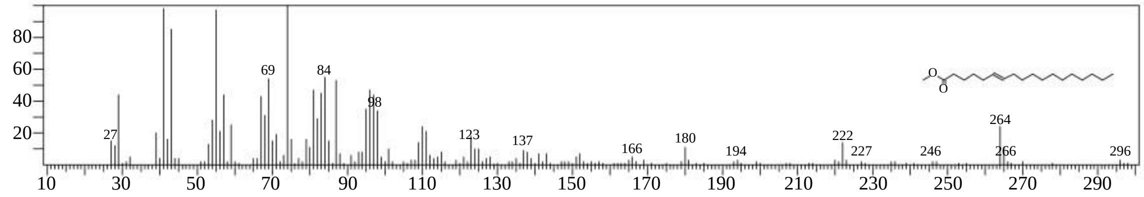
80
60
40
20
27 69 84 98 123 137 166 180 194 222 227 246 264 266 296
10 30 50 70 90 110 130 150 170 190 210 230 250 270 290

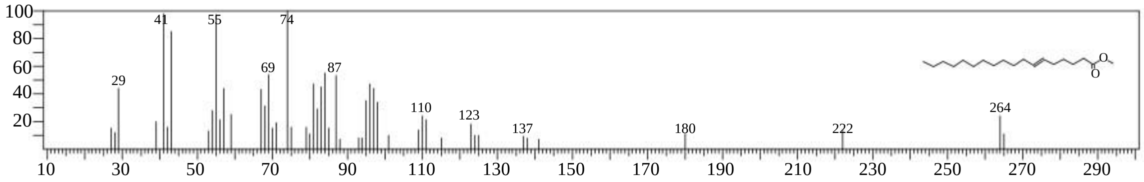
100
80
60
40
20
29 41 55 69 74 87 110 123 137 180 222 264
10 30 50 70 90 110 130 150 170 190 210 230 250 270 290

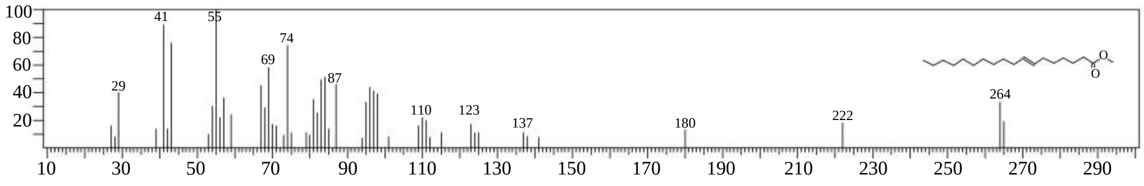
100
80
60
40
20
29 41 55 69 74 87 110 123 137 180 222 264
10 30 50 70 90 110 130 150 170 190 210 230 250 270 290

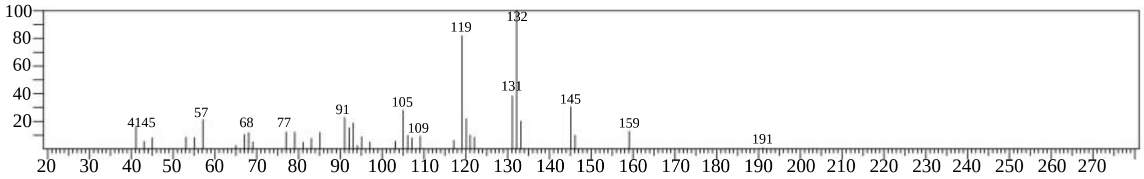

100
80
60
40
20
4145
57
68
77
91
105
109
119
131
132
145
159
191
20 30 40 50 60 70 80 90 100 110 120 130 140 150 160 170 180 190 200 210 220 230 240 250 260 270

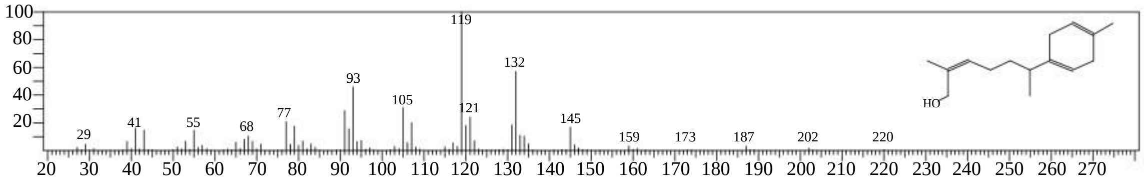

100
80
60
40
20
29
41
55
68
77
93
105
119
121
132
145
159
173
187
202
220
HO
20 30 40 50 60 70 80 90 100 110 120 130 140 150 160 170 180 190 200 210 220 230 240 250 260 270

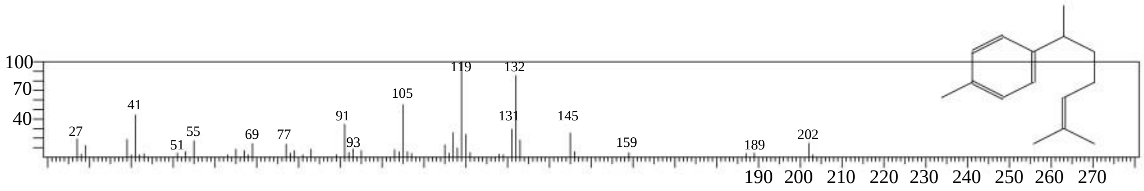

100
70
40
27
41
51
55
69
77
91
93
105
119
131
132
145
159
189
202
190 200 210 220 230 240 250 260 270

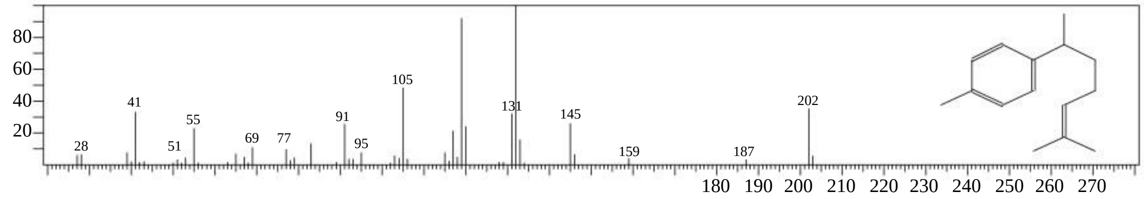

80
60
40
20
28
41
51
55
69
77
91
95
105
131
145
159
187
202
180 190 200 210 220 230 240 250 260 270

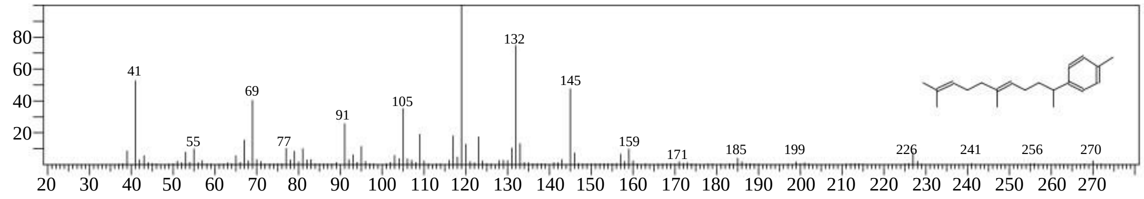

80
60
40
20
41
55
69
77
91
105
132
145
159
171
185
199
226
241
256
270
20 30 40 50 60 70 80 90 100 110 120 130 140 150 160 170 180 190 200 210 220 230 240 250 260 270

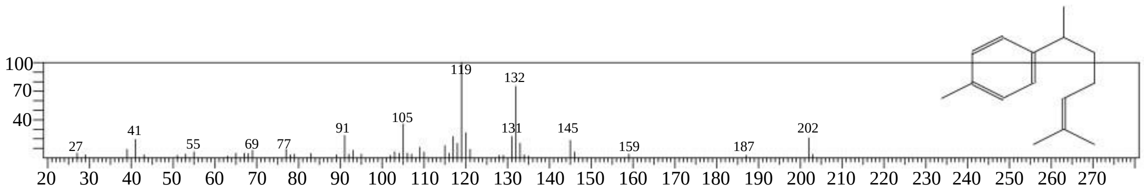

100
70
40
27
41
55
69
77
91
105
119
131
132
145
159
187
202
20 30 40 50 60 70 80 90 100 110 120 130 140 150 160 170 180 190 200 210 220 230 240 250 260 270

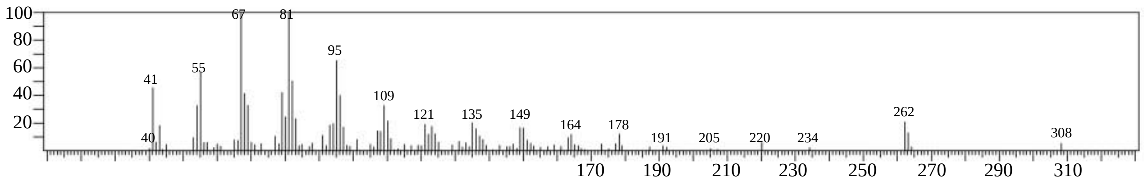
100
80
60
40
20
40 41 55 67 81 95 109 121 135 149 164 178 191 205 220 234 262 308
170 190 210 230 250 270 290 310

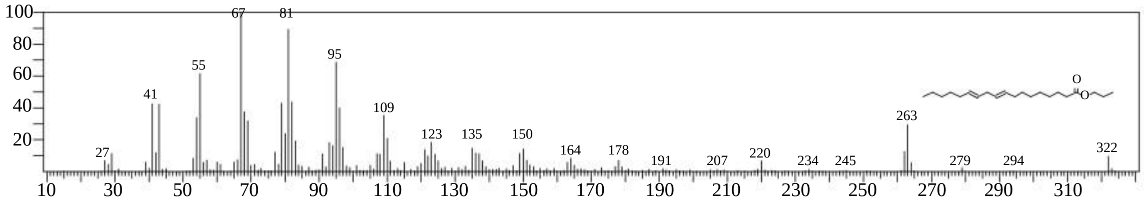
100
80
60
40
20
27 41 55 67 81 95 109 123 135 150 164 178 191 207 220 234 245 263 279 294 322
O
O
10 30 50 70 90 110 130 150 170 190 210 230 250 270 290 310

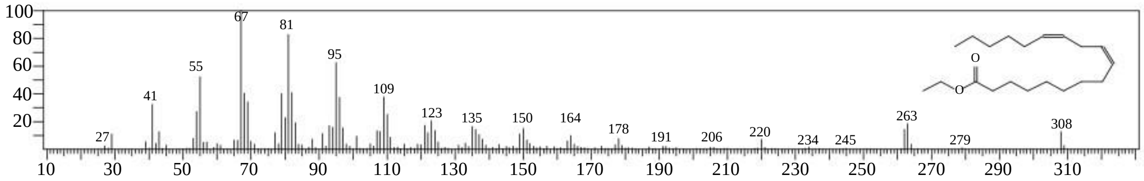
100
80
60
40
20
27 41 55 67 81 95 109 123 135 150 164 178 191 206 220 234 245 263 279 308
O
O
10 30 50 70 90 110 130 150 170 190 210 230 250 270 290 310

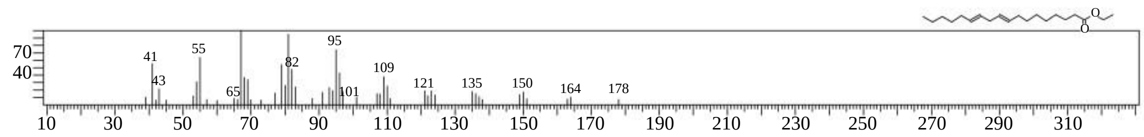
70
40
41 43 55 65 82 95 101 109 121 135 150 164 178
O
O
10 30 50 70 90 110 130 150 170 190 210 230 250 270 290 310

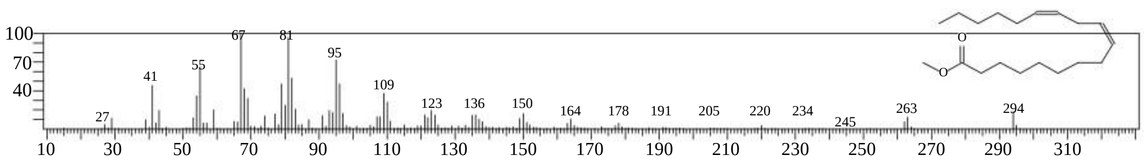
100
70
40
27 41 55 67 81 95 109 123 136 150 164 178 191 205 220 234 245 263 294
O
O
10 30 50 70 90 110 130 150 170 190 210 230 250 270 290 310

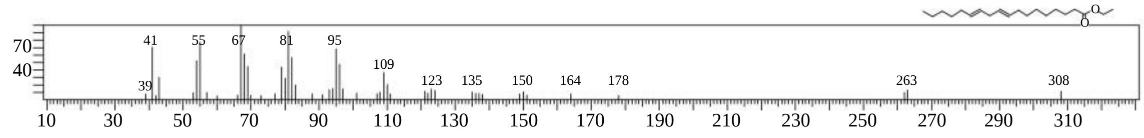
70
40
39 41 55 67 81 95 109 123 135 150 164 178 263 308
O
O
10 30 50 70 90 110 130 150 170 190 210 230 250 270 290 310

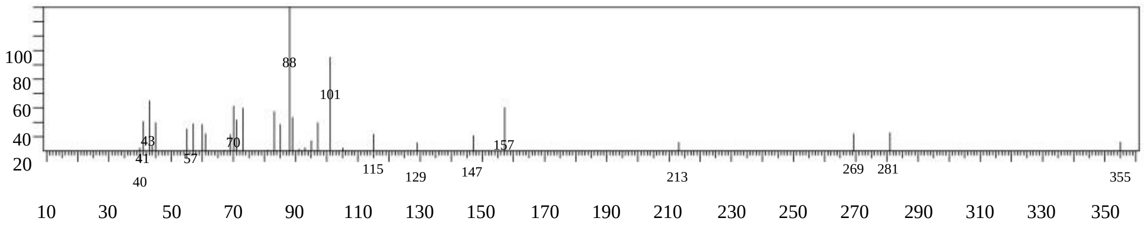

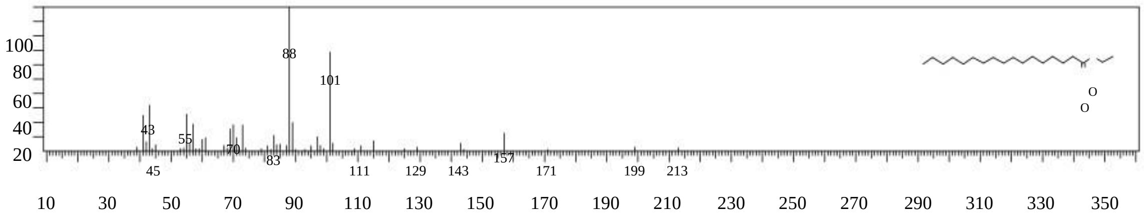

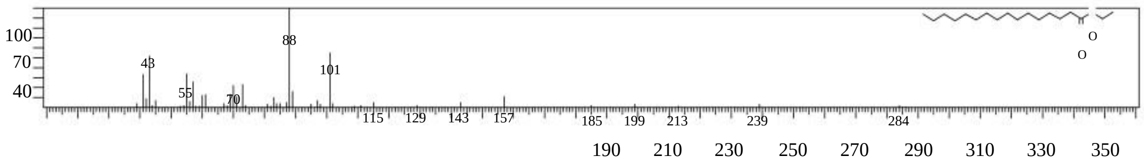

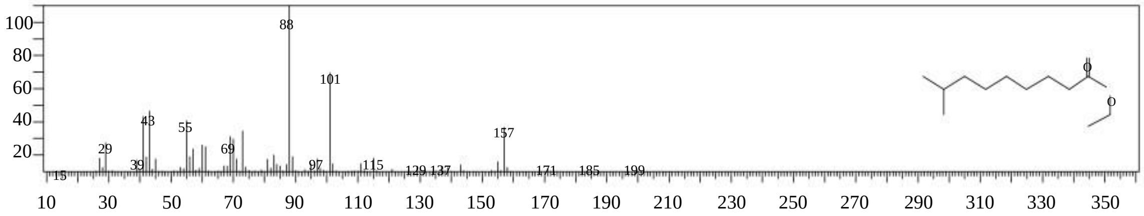

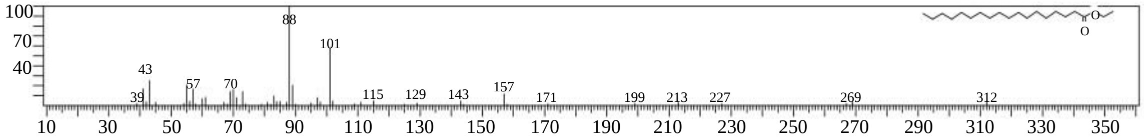

:5 Entry:201946 Library:WILEY8.LIB

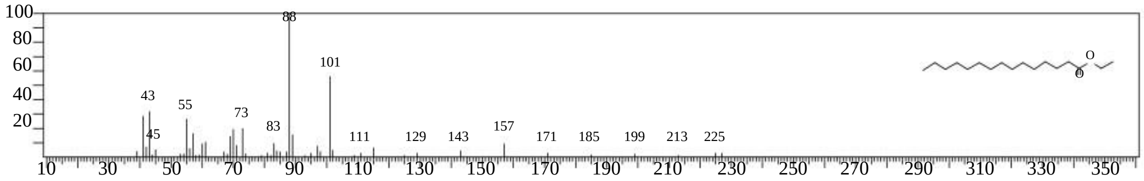

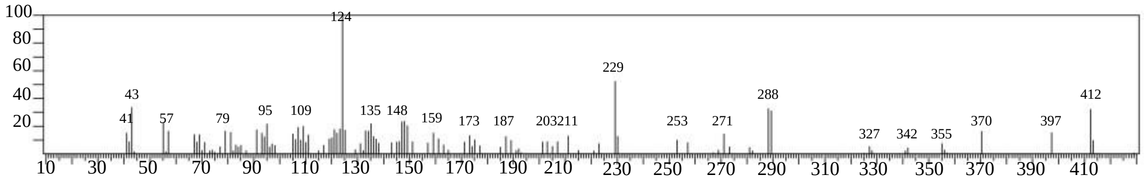
100
80
60
40
20
124
43
41
57
79
95
109
135
148
159
173
187
203211
229
253
271
288
327
342
355
370
397
412
10 30 50 70 90 110 130 150 170 190 210 230 250 270 290 310 330 350 370 390 410

:

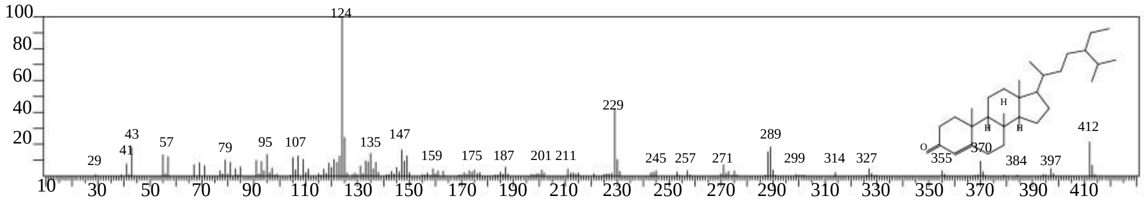
100
80
60
40
20
124
43
41
29
57
79
95
107
135
147
159
175
187
201 211
229
245
257
271
289
299
314
327
355
370
384
397
412
O
H
10 30 50 70 90 110 130 150 170 190 210 230 250 270 290 310 330 350 370 390 410

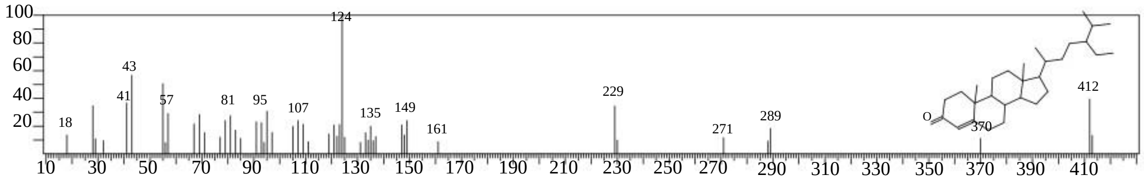
100
80
60
40
20
124
43
41
18
57
81
95
107
135
149
161
229
271
289
370
412
O
10 30 50 70 90 110 130 150 170 190 210 230 250 270 290 310 330 350 370 390 410

:

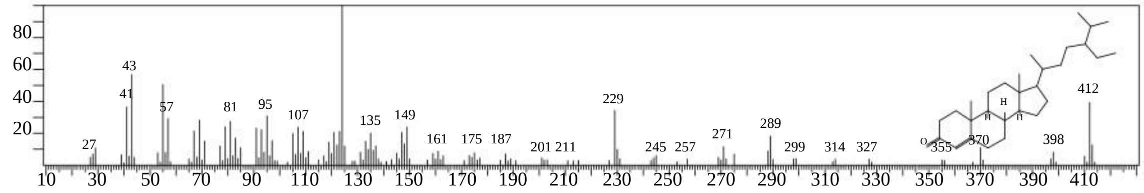
80
60
40
20
43
41
27
57
81
95
107
135
149
161
175
187
201 211
229
245
257
271
289
299
314
327
355
370
398
412
O
H
10 30 50 70 90 110 130 150 170 190 210 230 250 270 290 310 330 350 370 390 410

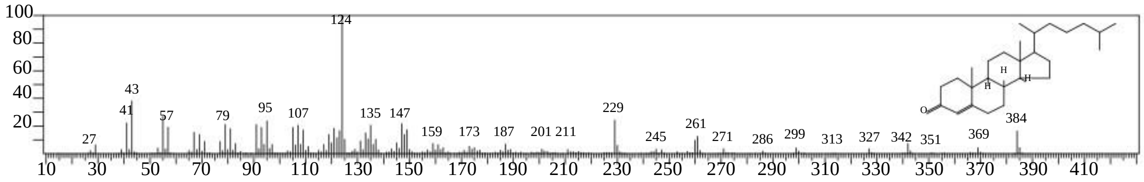
100
80
60
40
20
124
43
41
27
57
79
95
107
135
147
159
173
187
201 211
229
245
261
271
286
299
313
327
342
351
369
384
O
H
10 30 50 70 90 110 130 150 170 190 210 230 250 270 290 310 330 350 370 390 410

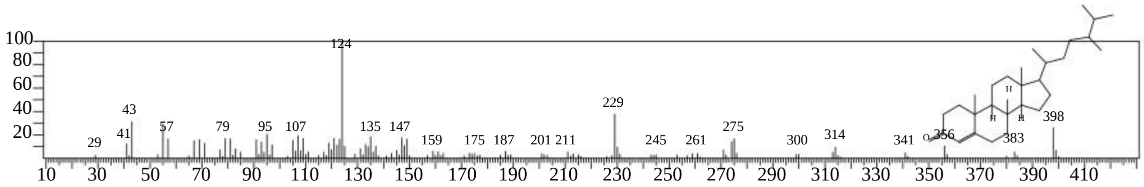
100
80
60
40
20
124
43
41
29
57
79
95
107
135
147
159
175
187
201 211
229
245
261
275
300
314
341
356
383
398
O
H
10 30 50 70 90 110 130 150 170 190 210 230 250 270 290 310 330 350 370 390 410
